# Magnetic Coupling Control in Triangulene Dimers

**DOI:** 10.1021/jacs.3c05178

**Published:** 2023-08-23

**Authors:** Hongde Yu, Thomas Heine

**Affiliations:** † Faculty of Chemistry and Food Chemistry, Technische Universität Dresden, Bergstraße 66c, 01062 Dresden, Germany; ‡ Institute of Resource Ecology, Helmholtz Zentrum Dresden-Rossendorf, Permoserstraße 15, 04318 Leipzig, Germany; § Department of Chemistry, Yonsei University, Seodaemun-gu, Seoul 120-749, Republic of Korea

## Abstract

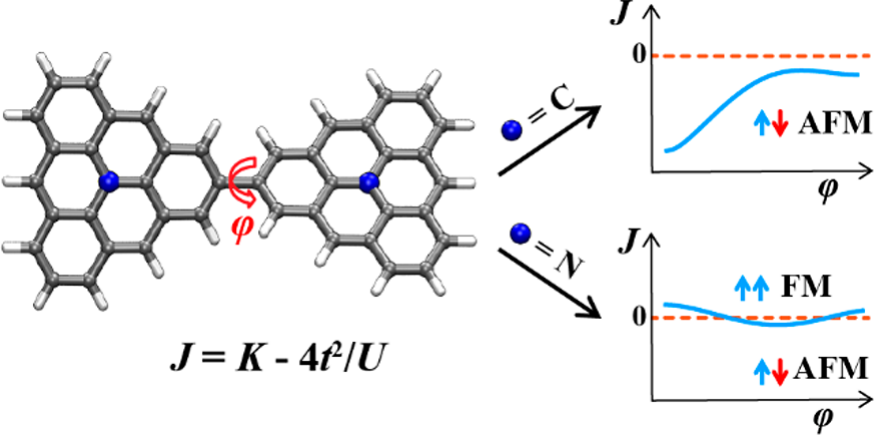

Metal-free magnetism remains an enigmatic field, offering prospects for unconventional magnetic and electronic devices. In the pursuit of such magnetism, triangulenes, endowed with inherent spin polarization, are promising candidates to serve as monomers to construct extended structures. However, controlling and enhancing the magnetic interactions between the monomers persist as a significant challenge in molecular spintronics, as so far only weak antiferromagnetic coupling through the linkage has been realized, hindering their room temperature utilization. Herein, we investigate 24 triangulene dimers using first-principles calculations and demonstrate their tunable magnetic coupling (*J*), achieving unprecedented strong *J* values of up to −144 meV in a non-Kekulé dimer. We further establish a positive correlation between bandgap, electronic coupling, and antiferromagnetic interaction, thereby providing molecular-level insights into enhancing magnetic interactions. By twisting the molecular fragments, we demonstrate an effective and feasible approach to control both the sign and strength of *J* by tuning the balance between potential and kinetic exchanges. We discover that *J* can be substantially boosted at planar configurations up to −198 meV. We realize ferromagnetic coupling in nitrogen-doped triangulene dimers at both planar and largely twisted configurations, representing the first example of ferromagnetic triangulene dimers that cannot be predicted by the Ovchinnikov rule. This work thus provides a practical strategy for augmenting magnetic coupling and open up new avenues for metal-free ferromagnetism.

## Introduction

Polycyclic aromatic hydrocarbons (PAHs) have emerged as a promising class of organic molecules with the potential for nontraditional electronics and spintronics.^1−5^ Among them, triangulene is particularly noteworthy, as it features the smallest nanographene with a triplet ground state, which has sparked great research interest since its first synthesis.^6−8^ Its intriguing metal-free magnetism and captivating non-Kekulé structure, where all of the 22 π-electrons are impossible to concomitantly pair into a closed-shell structure, offer immense prospects for the development of next-generation molecular spintronic materials, such as spin filters and qubits for quantum computing. Triangulene has been proposed as a prototype for studying other PAHs, which can be doped, substituted, or incorporated into more complex architectures.^7,9−11^ Recent developments in the synthesis of extended structures made of triangulenes have led to the observation of many exotic phenomena, such as Dirac cones,^12^ collective spin excitation,^7,10,13^ and Haldane phase.^11^ Despite the successful realization of triangulene monomers,^6^ dimers,^10^ nanostars,^13^ quantum rings,^14^ and 1D polymers,^11^ as well as its larger homologues such as [4]-, [5]-, and [6]-triangulene,^6,15−17^ the chemical versatility of triangulene and its derivatives remains understudied, especially for those with heteroatoms.^9,18−22^ For hydrocarbons with a bipartite honeycomb lattice, the well-established Ovchinnikov rule,^23^ i.e. Lieb’s theorem,^24^ is commonly applied to predict the spin-polarized ground states of the non-Kekulé molecules. The total spin of the system emerges from half-filling of the degenerate highest occupied molecular orbitals (HOMOs) according to Hund’s rule, and the multiplicity is given by *S* = (*N*_A_ – *N*_B_)/2, where *N*_A_ and *N*_B_ are the numbers of sites belonging to the two honeycomb sublattices. Therefore, a different number of sites in the sublattices is crucial to achieve molecules with high-spin ground states.^25,26^ One promising strategy involves the utilization of meta-connected phenyl rings.^10,27−29^ However, introducing heteroatoms, such as O, N, and B, can break the condition of Lieb’s theorem and thus go beyond the predictive capabilities of Ovchinnikov’s rule. This may open doors for realizing unconventional metal-free magnetism. For example, nitrogen-doped triangulene, or aza-triangulene, has been observed to undergo Jahn–Teller distortion,^30^ lowering the symmetry from the intuitive *D*_3*h*_ to *C*_2*v*_.^9^

Despite triangulene homologues, achieving a high-spin ground state and ferromagnetic (FM) interactions in nanographenes remains a significant challenge,^17^ although two-dimensional (2D) FM networks have been reported by Anindya and Rochefort arising from their kagome lattices.^31^ While single-bond linked PAHs can be easily constructed via the Yamamoto or Ullmann coupling reactions and show great potential for building extended periodic structures,^10,11,32,33^ covalently bonded triangulene derivatives, such as triangulene dimers,^10^ nanostars,^13^ and linear polymers,^11^ currently only exhibit antiferromagnetic (AFM) interactions and open-shell singlet (OSS) ground states. Among them, triangulene dimers, also referred to as *n* = 2 oligomers,^11^ usually serve as a gauge for assessing the magnetism of more extended 1D and 2D polymers. However, to date, only weak magnetic coupling has been achieved, such as −14 meV for directly linked dimers and −2 meV for phenyl-bridged dimers.^10^ This limits their practical application in computation and data storage, as they fall below the Landauer limit (i.e., the threshold for minimum energy dissipation in a state change during computation) at room temperature, which is 18 meV.^34,35^ Therefore, it is essential to develop strategies to enhance the magnetic coupling in triangulene dimers for both a fundamental understanding and practical applications. Furthermore, while only antiferromagnetism has been observed in dimers,^11,36−39^ the search for robust ferromagnetism in organic magnets is a long-standing research goal in metal-free magnetism.^40,41^ The Goodenough–Kanamori rule is a general principle to predict and understand the strength and type of magnetic coupling by the angle and type of spin-polarized orbitals. According to this rule, the magnetic interactions are governed by both FM potential exchange *K* and AFM kinetic exchange, i.e. superexchange, −4*t*^2^/*U*, where *t* and *U* represent the electronic coupling and Coulomb repulsion, respectively.^42,43^ This superexchange, arising from the virtual hopping of antiparalleled spins at adjacent sites, usually dominates the spin configuration in organic magnets, owing to the strong electronic coupling in π-conjugated systems.^44,45^ However, it is promising to manipulate the balance between these two exchange sources and realize metal-free FM nanographenes with high-spin states. Consequently, it is of significance to explore and engineer the spin interactions in triangulene dimers, which will provide profound insights into the magnetic properties of extended systems.

**Figure 1:**
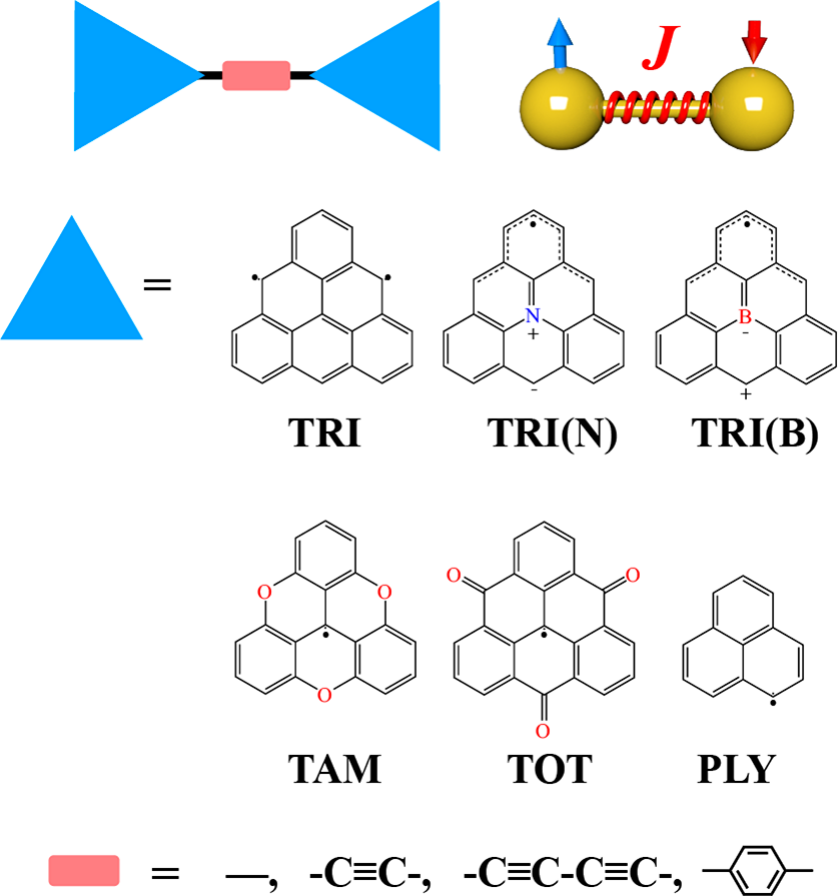
Structure illustration of triangulene dimers.

In this study, we investigate the metal-free magnetism in 24 triangulene dimers as well as their derivatives with various bridging groups, including −C≡C–, −C≡C–C≡C–, and phenyl linkages (Figure 1). By using broken-symmetry (BS-) density functional theory (DFT) calculations, we demonstrate that these non-Kekulé dimers exhibit a broad spectrum of spin couplings from −0.07 to −144 meV, including many exceeding the Landauer limit at room temperature, such as triarylmethyl (TAM), trioxotriangulene (TOT) dimers. Our analysis of their spin-polarized molecular orbitals sheds light on the origin of the magnetic interaction, where the kinetic exchange dominates due to strong electronic coupling. Additionally, we establish a positive correlation between bandgap, electronic coupling, and AFM magnetic couplings, which serves as a promising strategy for enhancing magnetic interactions. Most importantly, we demonstrate that by twisting the dihedral angles between the monomer fragments FM coupling of 1.2 meV can be achieved in nitrogen-doped triangulene dimers through a delicate balance of potential and kinetic exchange. This is confirmed by complete active space self-consistent field (CASSCF)/n-electron valence state perturbation theory (NEVPT2) calculations, giving a significant *J* value of 23.6 meV. Our results deepen the understanding of the metal-free magnetism of triangulene dimers, and provide new avenues for the design of molecular-based magnetic materials as well as the regulation of their magnetic interactions.

## Results and Discussion

### 1 Triangulene Analogues from Monomers to Dimers

Triangulene (TRI) is the smallest PAH diradical with a triplet ground state.^46^ Analogously, monoradicals such as nitrogen-doped triangulene (TRI(N)), boron-doped triangulene (TRI(B)), TAM, TOT, and phenalenyl (PLY) have also been synthesized either in solution or on the surface (Figure 1).^6,9,18,20,46−49^ Among them, TRI, TAM, TOT, and PLY have *D*_3*h*_ symmetry,^6^ while TRI(N) and TRI(B) exhibit lower symmetry of *C*_2*v*_ due to Jahn–Teller distortion.^9^ The *C*_2*v*_ configuration is more energetically favorable than the *D*_3*h*_ configuration by 5.63 and 5.58 kJ/mol, resulting in the distorted symmetry for TRI(N) and TRI(B) (Table S1). Despite the *D*_3*h*_ symmetry of TRI’s chemical structure and consequently of its spin density distribution, it is noted that the two degenerate singly occupied molecular orbitals (SOMO), ψ_1_ and ψ_2_, only exhibit *C*_2*v*_ symmetry, while their overall density, |ψ_1_|^2^ + |ψ_2_|^2^, has *D*_3*h*_ symmetry (Figure S1).^6^ In comparison, both TRI(N) and TRI(B) monomers show Jahn–Teller-distorted *C*_2*v*_ symmetry. This structural symmetry break is reflected in the spin density distributions of the same symmetry (Figures S2 and S3).^9,48^ In contrast, the SOMOs of TOT, TAM, and PLY show *D*_3*h*_ symmetry, consistent with the symmetry of their chemical structures and spin density distributions (Figures S4–S6).

**Figure 2:**
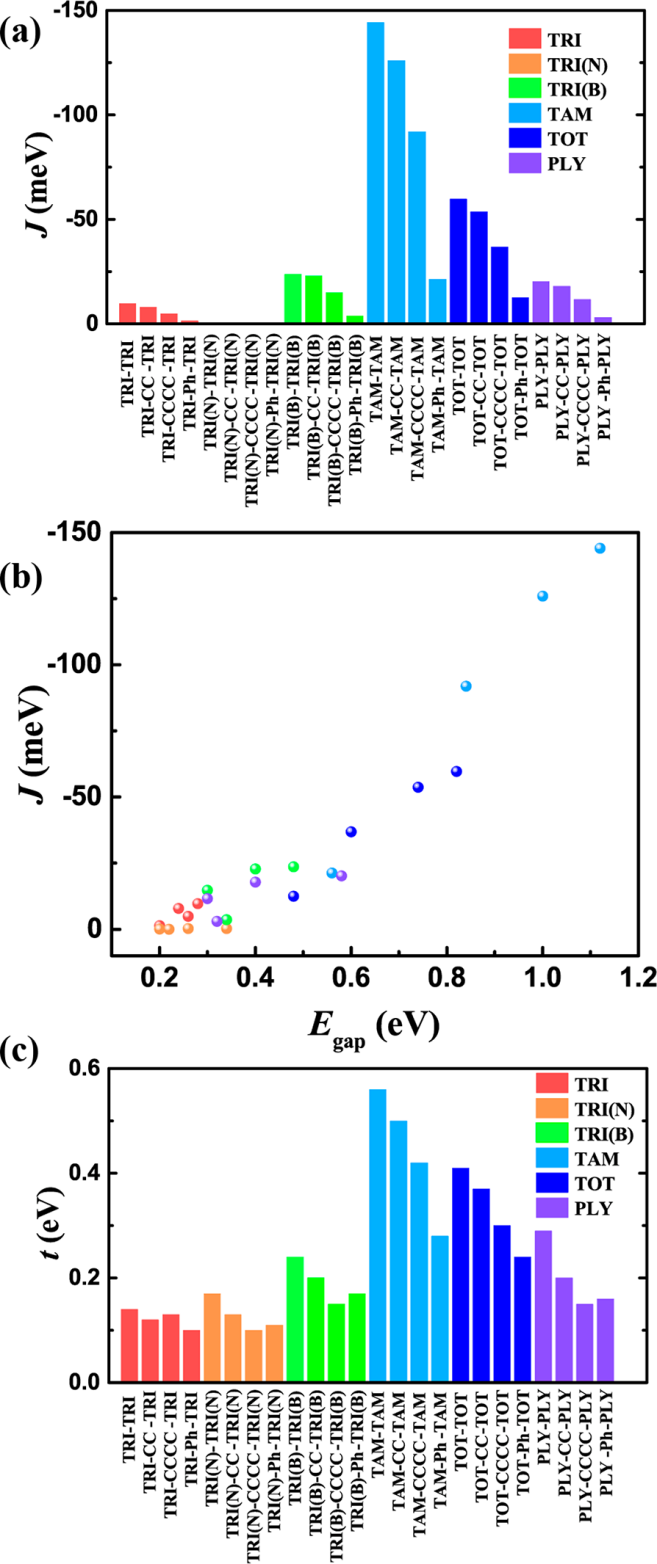
Distribution of magnetic coupling (*J*) (a) and electronic coupling (*t*) (c) in triangulene dimers. (b) Positive correlation between *J* and the HOMO–LUMO gap (*E*_gap_).

Using these monomers as building blocks, we constructed 24 dimers that are connected either directly or bridged by −C≡C– (CC), −C≡C–C≡C– (CCCC), and phenyl (Ph) linkages (Figure 1). These molecules are fully conjugated non-Kekulé radicals. Although TAM-TAM and TOT-TOT have Kekulé resonance structures, the open-shell non-Kekulé diradicals are more stable than the closed-shell Kekulé structure by 8.69 and 32.81 kJ/mol, respectively, due to the recovery of the benzene ring and energy gain of forming aromatic π-sextets as per Clar’s rule (Table 1 and Figure S7).^50^ The molecules of the TRI-series (including TRI-TRI, TRI-CC-TRI, TRI-CCCC-TRI, and TRI-Ph-TRI) have four unpaired electrons, while the other dimers constructed by monoradicals have two spins. We optimized the structures of these radicals as free molecules and calculated their magnetic properties at the PBE0/def2-TZVP level with the Gaussian 16 package^51,52^ (see computational details for more information; for validation see ref (53)). We find that all of them have OSS ground states and thus AFM interactions between the monomers (Table 1 and Figure 2a). As expected, all directly connected dimers are twisted along the connecting single bond as free molecules due to the steric hindrance of hydrogen atoms (Figure S8). The spin densities in the dimers are mostly localized on the monomer building units, where they maintain the local symmetry as in the monomers (Figures S9–S18). To evaluate the strength of magnetic interactions, we calculate the magnetic coupling *J* as defined from the Heisenberg–Dirac–van Vleck (HDVV) Hamiltonian
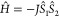
where *Ŝ*_1_ and *Ŝ*_2_ are the spin angular momentum operators on sites 1 and 2, respectively.^54^*J* is calculated by the normalized energy difference between the low-spin and high-spin states, where *J* > 0 implies FM and *J* < 0 AFM interaction (see computational details in Supporting Information).

**Table 1:** Magnetic Properties of the TRI, TRI(N), TRI(B), TOT, TAM, and PLY Dimers As Free Molecules, Including Magnetic Coupling (*J*), Electronic Coupling (|*t*|), Coulomb Repulsion (*U*), Kinetic (−4*t*^2^/*U*), and Potential (*K*) Exchange^a^

	*J* (meV)	|*t*| (eV)	*U* (eV)	–4*t*^2^/*U* (eV)	*K* (eV)
TRI-TRI	–9.73	0.14	1.99	–0.04	0.03
TRI-CC-TRI	–7.91	0.12	2.16	–0.03	0.02
TRI-CCCC-TRI	–4.94	0.13	2.18	–0.03	0.03
TRI-Ph-TRI	–1.45	0.10	2.20	–0.02	0.02
TRI(N)-TRI(N)	–0.33	0.17	0.92	–0.12	0.12
TRI(N)-CC-TRI(N)	–0.33	0.13	1.01	–0.07	0.07
TRI(N)-CCCC-TRI(N)	–0.13	0.10	1.03	–0.04	0.04
TRI(N)-Ph-TRI(N)	–0.07	0.11	1.02	–0.05	0.05
TRI(B)-TRI(B)	–23.71	0.24	0.91	–0.25	0.23
TRI(B)-CC-TRI(B)	–22.86	0.20	1.01	–0.16	0.14
TRI(B)-CCCC-TRI(B)	–14.83	0.15	1.07	–0.08	0.07
TRI(B)-Ph-TRI(B)	–3.67	0.17	1.06	–0.10	0.10
TAM-TAM	–144.15	0.56	0.09	–13.84	13.69
TAM-CC-TAM	–126.00	0.50	0.14	–7.18	7.06
TAM-CCCC-TAM	–91.97	0.42	0.24	–3.00	2.91
TAM-Ph-TAM	–21.35	0.28	0.56	–0.56	0.54
TOT-TOT	–59.69	0.41	0.34	–1.98	1.92
TOT-CC-TOT	–53.71	0.37	0.37	–1.45	1.39
TOT-CCCC-TOT	–36.86	0.30	0.46	–0.79	0.75
TOT-Ph-TOT	–12.53	0.24	0.60	–0.38	0.37
PLY-PLY	–20.23	0.29	0.85	–0.40	0.38
PLY-CC-PLY	–17.93	0.20	0.97	–0.16	0.14
PLY-CCCC-PLY	–11.63	0.15	1.02	–0.09	0.08
PLY-Ph-PLY	–3.03	0.16	1.00	–0.11	0.10

a *K* is derived from the Goodenough–Kanamori rule.

As shown in Figure 2a, the dimers exhibit a wide range of magnetic couplings, spanning from −0.07 to −144 meV. This diversity of spin interactions can provide a valuable toolkit for designing novel metal-free magnetic materials and offers fundamental insights into the magnetic behavior of 1D and 2D polymers. It is worth noting that the value of *J* for TRI-TRI as a distorted free molecule is −9.7 meV, whereas it is −20 meV for the planar configuration (Table 2). This is in agreement with the previously reported experimental observation of −14 meV for the triangulene dimer on the Au(111) surface.^10^ TRI-Ph-TRI also has an experimentally known singlet–triplet energy gap of 2 meV, which matches our calculation (Table 1). Among these molecules, TAM-TAM shows a remarkably strong magnetic interaction of −144 meV, which is more than 10 times larger than that in TRI-TRI, indicating a robust antiparallel spin ordering at high temperatures, but also other dimers show strong couplings. For example, TOT-TOT, TOT-CC-TOT, and PLY-PLY have *J* values of −60, −54, and −20 meV, respectively, and exceed the Landauer limit of minimum energy dissipation of 18 meV.^7,34^ This makes them potential candidates for spin logic operations at ambient temperature (Table 1 and Figure 2a).

**Table 2:** Magnetic Properties Calculated at the PBE0/def2-TZVP Level for the TRI-TRI, TRI(N)-TRI(N), TRI(B)-TRI(B), TOT-TOT, TAM-TAM, and PLY-PLY in Planar Configurations and TRI(N)CH_3_-TRI(N)CH_3_, Including Magnetic Coupling (*J*), Electronic Coupling (|*t*|), Overlap Integral in Planar (*S*), Coulomb Repulsion (*U*), Kinetic (−4*t*^2^/*U*), and Potential (*K*) Exchange^a^

	*J* (meV)	|*t*| (eV)	*S*	*S*_free_	*U* (eV)	–4*t*^2^/*U* (eV)	*K* (eV)
TRI-TRI	–20.22	0.143	0.032	0.025	1.768	–0.047	0.026
TRI(N)-TRI(N)	1.23	0.230	0.544	0.137	0.733	–0.289	0.291
TRI(B)-TRI(B)	–53.70	0.298	0.153	0.050	0.650	–0.546	0.493
TAM-TAM	–198.44	0.616	0.526	0.448	0.181	–8.380	8.182
TOT-TOT	–79.47	0.444	0.389	0.344	0.268	–2.945	2.865
PLY-PLY	–44.62	0.389	0.131	0.048	0.650	–0.931	0.886
TRI(N)CH_3_-TRI(N)CH_3_	0.30	0.240		0.460	0.718	–0.321	0.321

a The overlap integral of non-planar configurations corresponding to optimized geometries of free molecules (*S*_free_) are shown for comparison.

## 2 Molecular Orbital Perspective of Magnetic Interactions

**Figure 3:**
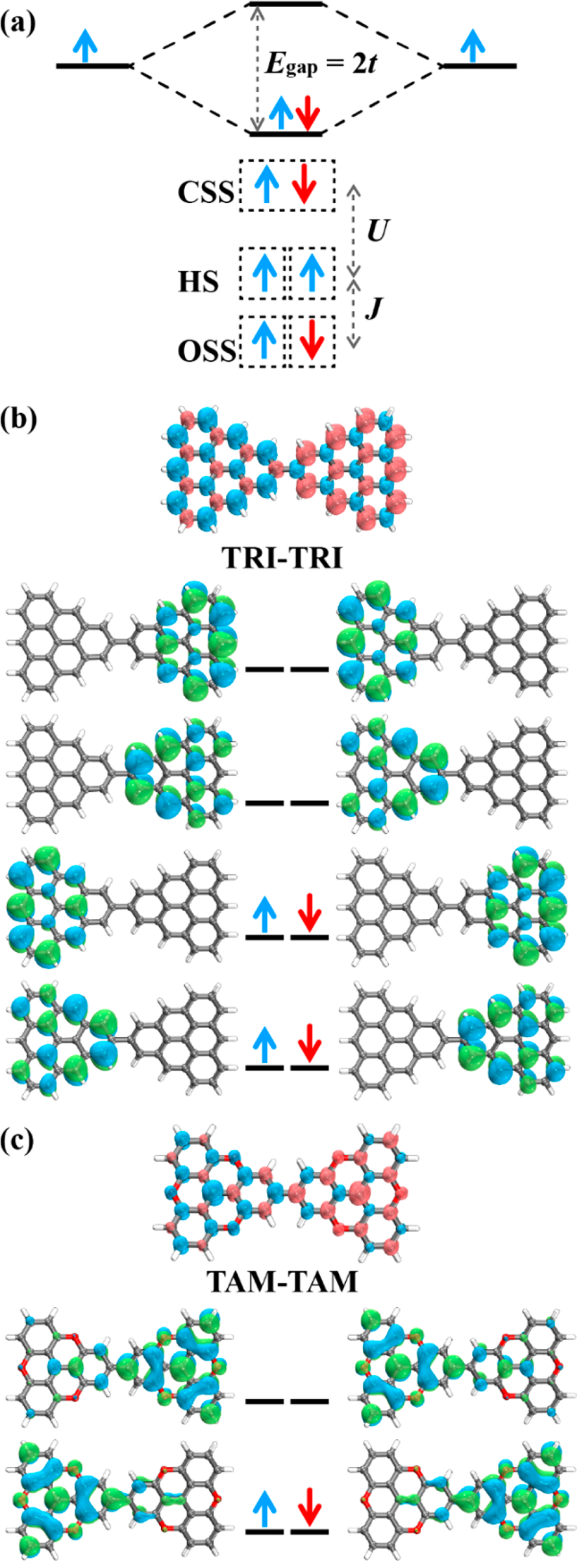
(a) Energy level diagram of molecular orbitals of monomer and dimer, and the closed-shell singlet (CSS), open-shell singlet (OSS) and high-spin state (HS). HOMO–LUMO gap *E*_gap_, hopping integral *t*, magnetic coupling *J* and on-site Coulomb repulsion *U* are also depicted. Spin density and frontier molecular orbital distribution of TRI-TRI (b) and TAM-TAM (c) for the BS states.

Metal-free magnetic systems are often characterized by delocalized molecular orbitals, especially for the HOMOs and the lowest unoccupied molecular orbitals (LUMOs), in contrast to inorganic complexes where atomic orbitals, i.e. d- or f-orbitals of transition metal elements, dominate the magnetic interactions.^6,44,55^ To elucidate the magnetic properties of these systems, we performed a comprehensive analysis of the frontier molecular orbitals of the dimers (Figure 3 and Table 1). They are essentially the superposition of the frontier orbitals of their respective monomers. The electronic coupling strength between the fragment orbitals in the dimers can be quantified by the hopping or transfer integral (Table 1). This can be approximated by the HOMO–LUMO gap of the dimer (Figure 3a):^56,57^
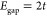
In addition to the OSS ground state, the high-spin and closed-shell states are also required, as their energy difference indicates the strength of the electronic correlation in spin-dimer systems, which is related to the on-site Coulomb repulsion term, *U*, in the Hubbard model (Figure 3a).^42^ In general, according to the Goodenough–Kanamori rule,^42,58^ the magnetic coupling *J* is dominated by both potential exchange and kinetic exchange, as formulated by
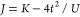


The potential exchange *K* arises from the direct exchange between parallel spins and promotes stable high-spin states and FM interactions, in accordance with Hund’s rule.^54^ Conversely, the kinetic exchange, −4*t*^2^/*U*, stems from the virtual hopping between neighboring sites with antiparallel spins and favors AFM interactions. Therefore, FM potential exchange is fundamentally short-ranged, whereas AFM potential exchange is a long-range interaction mediated by electronic coupling.^59^ Owing to the strong electronic coupling between adjacent orbitals, the kinetic exchange typically predominates the magnetic coupling, resulting in AFM interactions in organic systems and metal-oxide complexes.^36,37,40,60^ This poses a substantial challenge in achieving stable metal-free ferromagnetism.

To enhance our comprehension of the magnetic coupling in these dimers, we examined the interactions between the degenerate spin-polarized orbitals. As illustrated in Figure 3b and 3c, the SOMOs of TRI-TRI and TAM-TAM are derived from their monomer orbitals. The up and down spins are situated on neighboring fragments, signifying the prevailing influence of the kinetic exchange and AFM interactions. The two SOMOs of the TRI monomer are energetically degenerate. In the dimer, the SOMOs remain restricted within the monomers. However, the energetic degeneracy is lifted, and four single-occupied orbitals are present (Figure 3b). On the contrary, a stronger molecular overlap of the fragment orbitals gives extended SOMOs in TAM-TAM (Figure 3c). This is quantified by the much larger overlap integral of 0.448 and hopping integral of 0.56 eV in TAM-TAM, compared to 0.025 and 0.14 eV, respectively, in TRI-TRI. Consequently, the promoted electronic coupling in TAM-TAM enhances the kinetic exchange, yielding a stronger AFM coupling of −144 meV, ∼15 times higher than that of −9.7 meV in TRI-TRI. Furthermore, HOMO–LUMO gaps larger than 0.5 eV show a positive correlation with the antiferromagnetic couplings in these spin dimer systems, as the kinetic exchange is enhanced by a larger hopping integral (Figure 2b).^61−63^ Hence, promoting the overlap of SOMOs and thus the electronic coupling between the fragments offers a prospective approach to enhancing the magnetic interaction. This offers a rational design concept that focuses on non-Kekulé multiradicals with a large bandgap and high symmetry. We note that SOMOs with *D*_3*h*_ symmetry (TAM, TOT, and PLY) have larger hopping integrals than those with *C*_2*v*_ symmetry (TRI and TRI(N)) (Table 1 and Figure 2c). This means that the SOMOs with *D*_3*h*_ symmetry are more dispersive in the dimers, resulting in enhanced AFM couplings. The high-symmetry TAM-TAM, TOT-TOT, and PLY-PLY have AFM couplings of −144, −60, and −20 meV, respectively, with corresponding hopping integrals of 0.56, 0.41, and 0.29 eV. In contrast, TRI-TRI and TRI(N)-TRI(N) exhibit significantly weaker magnetic couplings of −9.7 and −0.3 meV with hopping integrals of 0.14 and 0.17 eV (Table 1 and Figure 2c). The electronic and magnetic couplings, *t* and *J*, decrease with the length of the spacer in the dimer from direct linkage toward −CC–, −CCCC–, and −Ph– spacers as the overlap between SOMOs weakens. It is worth noting that TRI(N) and TRI(B) dimers possess three unique electromers (electronic isomers), each with distinct spin configurations (Figures S14 and S15).^64^ These electromers have comparable energy levels (about 1 kcal/mol difference), each representing a distinct potential energy minimum (Tables S2 and S3). Based on their spin density distributions, they are categorized as head–head, head–tail, and tail–tail configurations, where the magnitude of the magnetic coupling follows the trend of head–head > head–tail > tail–tail due to the increasing distance between spin density centers (Tables S2 and S3). While the emergence of electromers is contingent upon the monomer geometry and external condition being used, tail–tail configurations demonstrate slightly enhanced stability for TRI(N) dimers, whereas head–head configurations are more stable for TRI(B) dimers (Tables S2 and S3). This is indicative of a distinctive class of isomerism, which is prevalent in transition metal complexes,^65,66^ but rarely observed in metal-free systems.^64,67,68^

The on-site Coulomb repulsion, *U*, also influences the overall magnetic interaction, especially for dimers with weak coupling (Figure 2b). It is primarily determined by the elementary monomer, as systems sharing the same building units have similar *U* values (Table 1). For instance, the *U* value of the TRI-series of molecules is around 2 eV, whereas that of the TRI(N)-series is approximately 1 eV, as the TRI monomer has two unpaired electrons, compared to only one spin in the TRI(N) monomer. Consequently, searching for non-Kekulé building blocks with unpaired π-electrons, such as Clar’s goblet^7^ and other triangulene systems,^13^ offers great promise for achieving metal-free spin-dimers and polymers with strong electron correlation. It is worth mentioning that, unlike the typical Hubbard model and inorganic metal complexes where the spins are fully localized on a single site,^42^ in metal-free systems, SOMOs are delocalized, and therefore, the *U* value is also affected by the conjugation, particularly for those with a large SOMO overlap and strong electronic coupling. This dispersive spin site is observed, for example, in TAM-TAM, yielding a small *U* value of 0.09 eV (Figure 3c and Table 1).

## 3 Manipulation of Magnetic Coupling from AFM to FM

**Figure 4:**
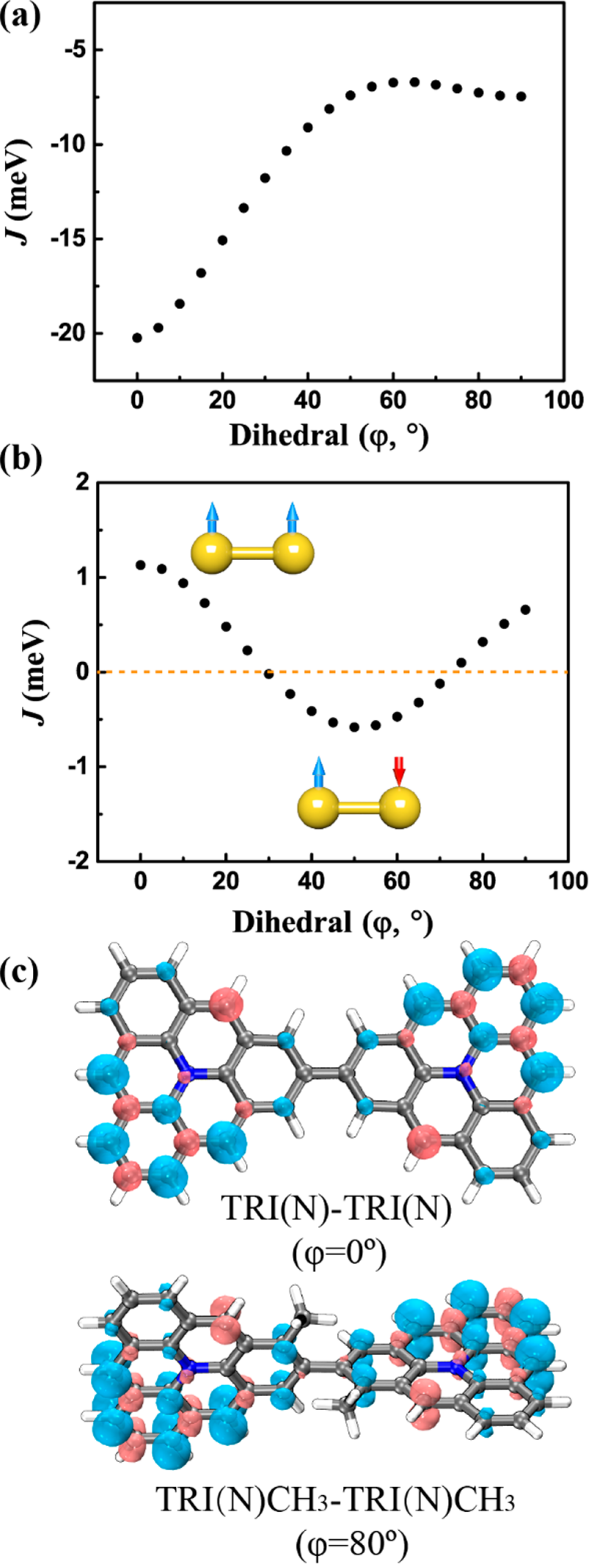
Relationship between magnetic coupling (*J*) and the dihedral angle (φ) of TRI-TRI (a) and TRI(N)-TRI(N) (b). The yellow dashed line represents *J* = 0. FM and AFM configurations are also shown in part b. (c) FM spin density distribution of TRI(N)-TRI(N) in a planar configuration and TRI(N)CH_3_-TRI(N)CH_3_.

The magnetic couplings in all of these dimers are primarily determined by the kinetic exchange, resulting in an AFM interaction and antiparallel spin alignment, as demonstrated earlier. However, the dihedral angle between the monomers can modify the overlap of SOMOs as well as the balance between kinetic and potential exchange. Although the monomers are planar, the directly connected dimers are distorted as free molecules. For example, TRI-TRI and TRI(N)-TRI(N) have dihedral angles of 37° and 38° in the free state due to the steric hindrance between neighboring H atoms (Figure S8). Controlling the dihedral angle between the monomers is another means to further regulate the magnetic interactions, as it can be planarized by surface deposition, but also fine-tuned by steric control units (SCUs).^69,70^ Our findings, as depicted in Figure 4a, demonstrate that a decrease in the dihedral angle φ results in an increase in the *J* value of TRI-TRI, which becomes the largest in the coplanar configuration. Specifically, planar TRI-TRI has a value of *J* = −20 meV, twice as large as that for the free dimer (*J* = −9.7 meV). This is attributed to the enhanced overlap integral of the SOMO by 28% between the building units (Table 2).^71^ This *J* value in the planar configuration corresponds to the on-surface structure, and is consistent with the experimental singlet–triplet gap of 14 meV for TRI-TRI on an Au(111) surface.^10^ Other directly linked dimers also enhance their magnetic couplings in their planar configurations, as shown in Table 2 and Figures S19–S22 for TOT-TOT, TAM-TAM, TRI(B)-TRI(B), and PLY-PLY. The largest *J* value of −198 meV, 54 meV higher than in the distorted configuration, is possessed by the planar TAM-TAM. This increase is, again, attributed to the promoted conjugation and correspondingly stronger overlap of SOMOs in the planar structure: the overlap integral rises from 0.448 to 0.526, and the electronic coupling from 0.56 to 0.62 eV (Table 2). In comparison, both the magnetic coupling and the overlap integral are suppressed for the perpendicular configuration (Table S4). Besides the Au(111) surface that shows strong electronic coupling with the adsorbates, many insulating substrates have been developed to synthesize nanographenes, such as TiO_2_(011) and Al_2_O_3_,^72,73^ which have less impact on their electronic and magnetic properties. It is important to note that attaining strong AFM coupling plays a crucial role in organic spintronics and quantum computing, which hold potential for data storage and processing applications with low energy consumption as well as facilitating long-range entanglement and robust quantum states.^7,74^

The magnetic behavior of TRI(N)-TRI(N) is particularly interesting, as we have observed that the sign of the magnetic coupling can be changed by varying the dihedral angle. Specifically, both FM and AFM interactions have been observed depending on the value of φ. For angles between 30° and ∼70°, the AFM interaction dominates and *J* < 0, whereas, for other angles, we predict FM interaction with *J* > 0. As a free molecule with a dihedral angle of 38°, TRI(N)-TRI(N) is an OSS with a *J* value of −0.3 meV. However, when attached to the substrate and transformed into a planar configuration (φ = 0°), the high-spin state becomes more stable with a *J* value of 1.2 meV (Table 2 and Figure 4b and 4c). In this case, the balance between potential (*K*) and kinetic exchange (−4*t*^2^/*U*) is crucial. As a free molecule, the value of −4*t*^2^/*U* is −124.3 meV, which slightly exceeds the *K* value of 124.0 meV, resulting in AFM interactions. As the value of φ decreases, both *K* and *t* increase due to the enhanced overlap of the SOMOs. Eventually, the potential exchange begins to surpass the kinetic exchange, leading to the FM interaction. This transformation from AFM to FM is a distinct characteristic, as in other systems investigated here, structural planarity promotes the AFM coupling. This phenomenon may be associated with the *C*_2*v*_ symmetry of the TRI(N) monomer and its SOMO, which creates a subtle balance between potential and kinetic exchanges that is also not observed in TRI(B)-TRI(B). The planar TRI(N)-TRI(N) has *C*_2*h*_ symmetry, where it is 5.98 kJ/mol more stable than in the *D*_2*h*_ configuration. We confirmed the prediction of FM coupling using CASSCF/NEVPT2, which yielded a *J* value of 23.6 meV. Various density-functionals, including B3LYP,^75^ MN15,^76^ M06-2X,^77^ and ωB97XD,^78^ significantly underestimate the *J* values with 1.1, 1.4, 0.8, and 2.2 meV, respectively (Table S5). Similarly, as the value of φ increases, *K* and *t* decrease and positive *J* values are observed when φ exceeds 70°. For a perpendicular configuration (φ = 90°), *J* = 0.7 meV. One of the simplest SCU for this system is the −CH_3_ group (denoted as TRI(N)CH_3_-TRI(N)CH_3_), which fixes the dihedral angle to φ = 80° and yields FM interaction of *J* = 0.3 meV (Table 2 and Figure 4c). These results demonstrate the first example of triangulene dimers with FM coupling and hold great potential to regulate the magnetic interactions in metal-free spin dimers and subsequent oligomers and polymers. This tunable magnetic interaction in TRI(N) dimer highlights the unconventional magnetism defying Ovchinnikov’s rule, and poses a significant challenge to the fundamental understanding of the metal-free magnetism in doped PAHs that do not satisfy Lieb’s theorem’s half-filling prerequisite, thereby necessitating further investigation of the underlying mechanisms to extend beyond the predictive capability of Ovchinnikov’s rule.

## Conclusions

In summary, we investigated the magnetic properties of 24 triangulene dimers by using first-principles calculations. Our results show that these dimers possess tunable magnetic interactions, spanning from −0.07 to −144 meV, with several dimers surpassing the Landauer limit at room temperature, such as TOT-TOT, TAM-TAM, TOT-CC-TOT, and PLY-PLY. Among them, TAM-TAM has the strongest *J* of −144 meV, making it a promising candidate for practical applications in organic spintronics. We found that the magnetic couplings in these dimers were primarily governed by kinetic exchange, resulting in antiparallel spin alignments. Additionally, we unveiled a positive correlation between bandgap, electronic coupling, and magnetic interaction, and propose a prospective approach to explore non-Kekulé radicals with a large bandgap and robust conjugation. We found that the dihedral angle between the monomers can subtly modify the balance between the kinetic and potential exchange, leading to either enhanced AFM coupling or the emergence of FM coupling. In most cases, the AFM coupling was significantly enhanced in planar structures due to the increase of SOMO overlap, corresponding to surface-adsorbed molecules being common, for example, in STM experiments, for which the calculated *J* value agrees well with the experimental singlet–triplet energy gap. The largest coupling for planar dimers was found for TAM-TAM with *J* = −198 meV. We discovered a transition from AFM to FM interaction in TRI(N)-TRI(N) with the variation of the dihedral angle, with a *J* value of 1.2 meV (confirmed by CASSCF/NEVPT2 calculations, with a value of 23.6 meV) observed in the planar structure and investigated the impact of steric control units to regulate the magnetic coupling. This approach offers a new avenue to regulate both the strength and the type of magnetic interactions and creates opportunities to design metal-free spin dimers and polymers with desirable magnetic properties for organic spintronics and quantum computing technologies. In summary, our study demonstrates the potential for controlling the magnetic behavior in triangulene dimers and paves the way for the development of new organic materials with tailored magnetic interactions.

## Supporting Information Available

Additional computational details, molecular structures, spin density distributions and frontier molecular orbitals of TRI, TRI(N), TRI(B), TAM, TOT, PLY monomers and dimers, relationship between magnetic coupling and dihedral angels for all above dimers (PDF)
