# Supplementary material for: Magnetic Coupling Control in Triangulene Dimers
Source: J Am Chem Soc. 2023 Aug 23;145(35):19303–11. doi: 10.1021/jacs.3c05178 (PMC10485925; doi:10.1021/jacs.3c05178)
Supplement: Supplementary file 1 — ja3c05178_si_001.pdf [file ja3c05178_si_001.pdf]

Supporting Information for

Magnetic Coupling Control in Triangulene Dimers

Hongde Yu† and Thomas Heine*†‡§

† Faculty of Chemistry and Food Chemistry, Technische Universität Dresden, Bergstraße 66c, 01062 Dresden, Germany.

‡ Institute of Resource Ecology, Helmholtz Zentrum Dresden-Rossendorf, Permoserstraße 15, 04318 Leipzig, Germany.

§ Department of Chemistry, Yonsei University, Seodaemun-gu, Seoul 120-749, Republic of Korea.

*Corresponding author. Email: thomas.heine@tu-dresden.de.

This PDF file includes:

Computational details

Figure S1. Molecular structure, spin density distribution and frontier molecular orbital of TRI monomer.

Figure S2. Molecular structure, spin density distribution and frontier molecular orbital of TRI(N) monomer.

Figure S3. Molecular structure, spin density distribution and frontier molecular orbital of TRI(B) monomer.

Figure S4. Molecular structure, spin density distribution and frontier molecular orbital of TAM monomer.

Figure S5. Molecular structure, spin density distribution and frontier molecular orbital of TOT monomer.

Figure S6. Molecular structure, spin density distribution and frontier molecular orbital of PLY monomer.

Figure S7. Comparasion of Kekulé and non-Kekulé structures of TAM-TAM and TOT-TOT.

Figure S8. Dihedral angles in the directly-linked dimers.

Figure S9. Spin density distribution and frontier molecular orbital of TRI(N)-TRI(N).

Figure S10. Spin density distribution and frontier molecular orbital of TRI(B)-TRI(B).

Figure S11. Spin density distribution and frontier molecular orbital of TOT-TOT.

Figure S12. Spin density distribution and frontier molecular orbital of PLY-PLY.

Figure S13. Spin density distributions of TRI-CC-TRI, TRI-CCCC-TRI and TRI -Ph-TRI.

Figure S14. Spin density distributions of TRI(N)-TRI(N), TRI(N)-CC-TRI(N), TRI(N)-CCCC-TRI(N) and TRI(N)-Ph-TRI(N).
Figure S15. Spin density distributions of TRI(B)-TRI(B), TRI(B)-CC-TRI(B), TRI(B)-CCCC-TRI(B) and TRI(B)-Ph-TRI(B).
Figure S16. Spin density distributions of TAM-CC-TAM, TAM-CCCC-TAM and TAM-Ph-TAM.
Figure S17. Spin density distributions of TOT-CC-TOT, TOT -CCCC-TOT and TOT-Ph-TOT.
Figure S18. Spin density distributions of PLY-CC-PLY, PLY-CCCC-PLY and PLY-Ph-PLY.
Figure S19. Relationship between magnetic coupling (*J*) and dihedral angle (φ) in PLY-PLY.
Figure S20. Relationship between magnetic coupling (*J*) and dihedral angle (φ) in TRI(B)-TRI(B).
Figure S21. Relationship between magnetic coupling (*J*) and dihedral angle (φ) in TAM-TAM.
Figure S22. Relationship between magnetic coupling (*J*) and dihedral angle (φ) in TOT-TOT.
Figure S23. Top and side view of the optimized geometry of Tr3N and Tr3B for their monomers and dimers.
Table S1. Comparison of TRI(N) and TRI(B) monomer.
Table S2. Electromers of TRI(N) dimers with different spin density distributions including head-head, head-tail and tail-tail.
Table S3. Electromers of TRI(B) dimers with different spin density distributions including head-head, head-tail and tail-tail.
Table S4. The magnetic coupling and overlap integral for TAM-TAM and TOT-TOT.
Table S5. Magnetic couplings of planar TRI(N)-TRI(N) calculated by different functionals.
Appendix: Optimized geometry at the PBE0/def2-TZVP level.

Computational details

The geometries of all the systems have been optimized at the PBE0/def2-TZVP level as implemented in the Gaussian16 (G16) program according to their ground states. The electronic structure calculations have been performed on these optimized geometries. We have used the default convergence criteria in G16 program for optimization and SCF, i.e. 0.000450 and 10^{-8}, respectively. As demonstrated in our previous study,[1] DFT calculations employing the PBE0 functional correctly predict the magnetic ground state of a series of triangular derivatives, and the magnetic coupling constants are calculated with a mean absolute error (MAE) of 12 meV with respect to CASSCF/NEVPT2 calculations. The optimized geometries have been shown in the appendix and ref[2]. For closed-shell singlet, we have performed restricted DFT calculations, while using unrestricted DFT calculations for the triplet and quintet. For OSS, the broken-symmetry (BS)-DFT approach has been employed. The stability of the DFT wave function has been optimized and checked with keyword "stable=opt". The magnetic couplings, J, have been extracted from BS-DFT calculations using Ruiz's approach[3] as defined by $J = -2(E_{\mathrm{HS}} - E_{\mathrm{OSS}})/(S_{max}(S_{max}+1))$, E_{HS} and E_{OSS} are the energies of high-spin and open-shell singlet states and S_{max} is the total spin of the high-spin state. The HS state is quintet for the TRI-series molecules, while it is triplet for other diradicals. Discussions and comparison about mapping approaches can be found in ref 1. As shown in Figure S23, other possible substitutions of triangulene monomers and dimers are also studied at PBE0/def2-TZVP level.[4] The emergence of FM coupling in planar TRI(N)-TRI(N) has also been validated by the CASSCF(6e,4o)/NEVPT2 calculations at def2-TZVP level as implemented in PySCF program giving a J value of 23.6 meV.

In order to evaluate the strength of the electronic correlation, the on-site Coulomb repulsion U has been estimated by the spin-polarization energy as defined by $U = E_{\mathrm{CSS}} - E_{\mathrm{HS}}$, where E_{HS} and E_{CSS} represent the energy of high-spin and closed-shell singlet states, according to the exact solution of the Hubbard model for the spin dimer system.[5] Although the restricted closed-shell wave function of the CSS state is not stable compared to OSS ground state due to the inherent electron correlation, it can be used

to estimate the strength of Coulomb repulsion and benefit the analysis of magnetic properties. To correctly determine the relative energy of the closed-shell singlet and high-spin state in planar TAM dimer, we have utilized the domain-based local pair natural orbital coupled-cluster theory (DLPNO-CCSD(T))[6] with ORCA 5.0 program.[7] This approach is necessary because the electron correlation is very weak in planar TAM dimer and the PBE0 calculations could not provide sufficient accuracy in this specific system. To expedite the SCF process, we have employed the "RIJCOSX" method, the auxiliary basis set def2-TZVP/C, and Coulomb fitting set def2/J for the correlated calculations.[8] Additionally, the "TightSCF" option has been applied to tighten the convergence settings. For the TRI-series molecules, we have considered both the closed-shell singlet and quintet states, while for other diradical systems, we have considered the closed-shell singlet and triplet states. The hopping integral t has been estimated from the HOMO-LUMO gap, E_{gap}, as defined by $t = E_{gap}/2$.[9,10] The potential exchange K has been calculated by $K = J + 4t^2 / U$ according to the Goodenough-Kanamori rule. To perform a magnetic coupling profile scan of dihedrals, we have applied the "opt=(modredundant, tight)" keyword, which allows to optimize the molecular geometry while constraining only the dihedral angle. The MultiWFN program has been used for the analysis of the overlap integral of SOMO and it defines the overlap integral S_{ij} of two orbitals as $S_{ij} = \int |\varphi_i(\mathbf{r})||\varphi_j(\mathbf{r})|d\mathbf{r}$, where $\varphi_i(\mathbf{r})$ and $\varphi_i(\mathbf{r})$ are the wavefunctions of SOMOs.[11]

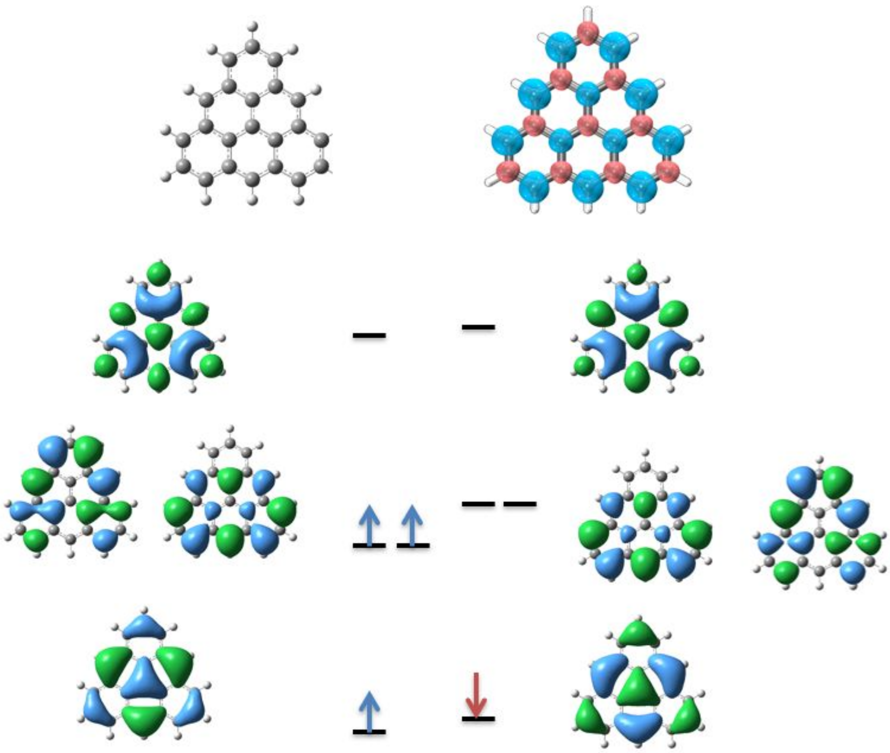

Figure S1. Molecular structure, spin density distribution and frontier molecular orbital of TRI monomer.

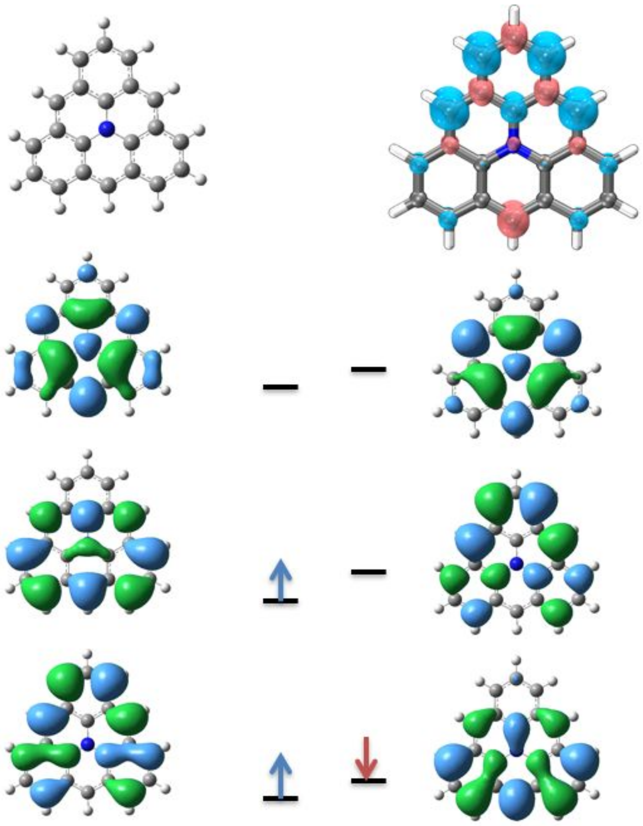

Figure S2. Molecular structure, spin density distribution and frontier molecular orbital of TRI(N) monomer.

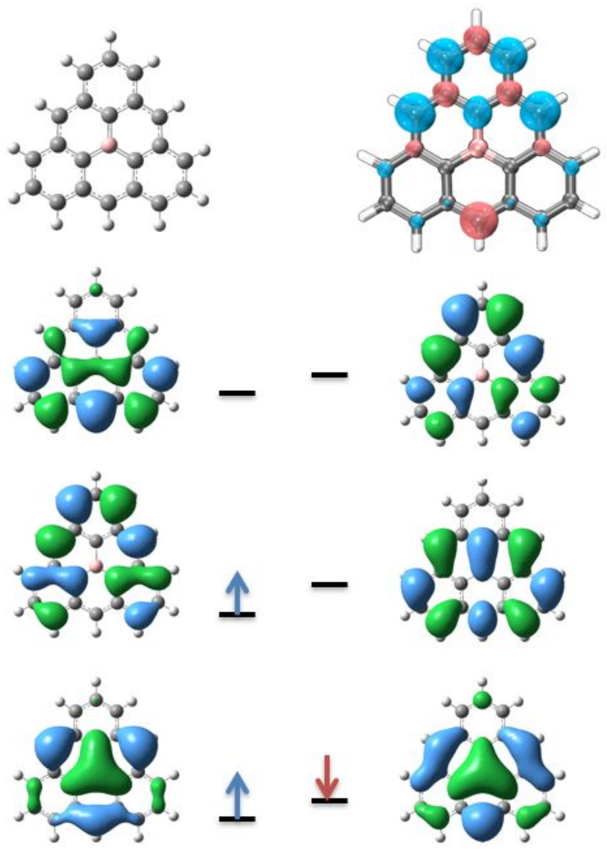

Figure S3. Molecular structure, spin density distribution and frontier molecular orbital of TRI(B) monomer.

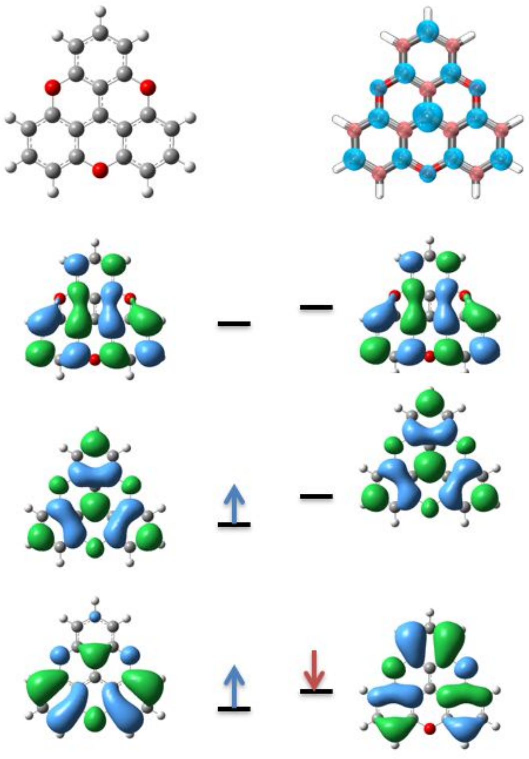

Figure S4. Molecular structure, spin density distribution and frontier molecular orbital of TAM monomer.

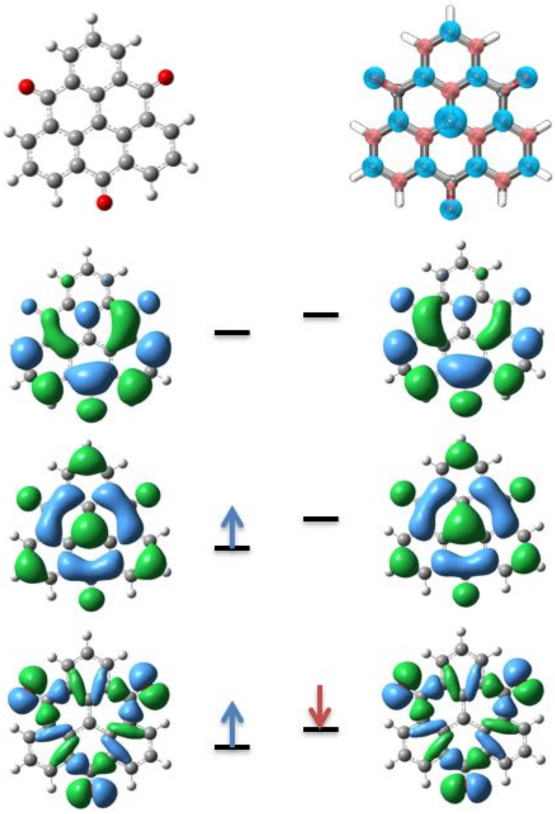

Figure S5. Molecular structure, spin density distribution and frontier molecular orbital of TOT monomer.

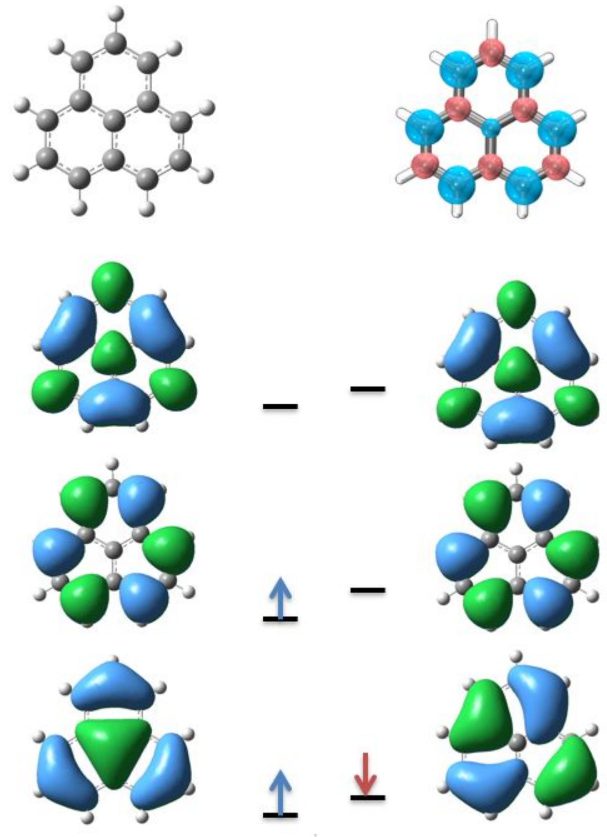

Figure S6. Molecular structure, spin density distribution and frontier molecular orbital of PLY monomer.

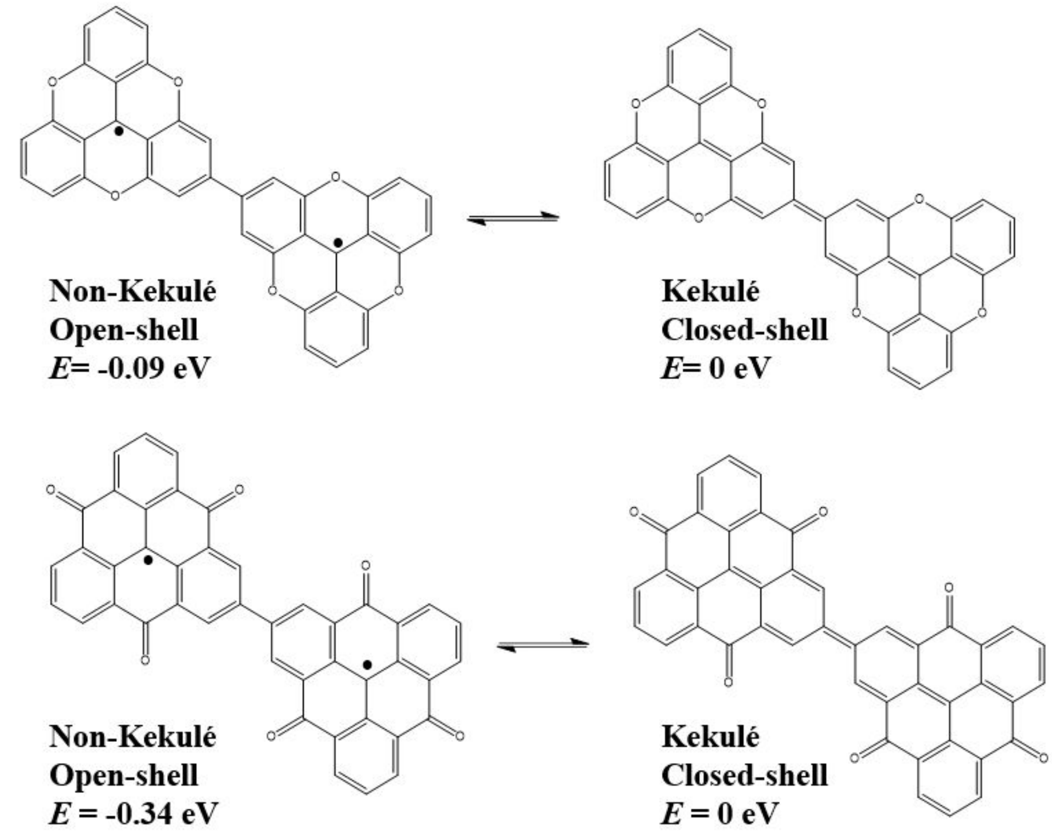

Figure S7. Comparasion of Kekulé and non-Kekulé structures of TAM and TOT dimers.

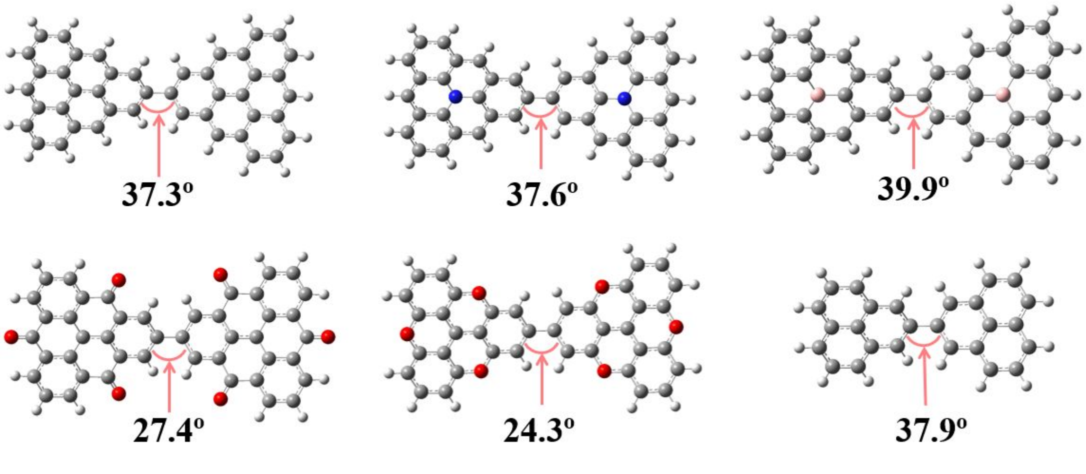

Figure S8. Dihedral angles in the dimers.

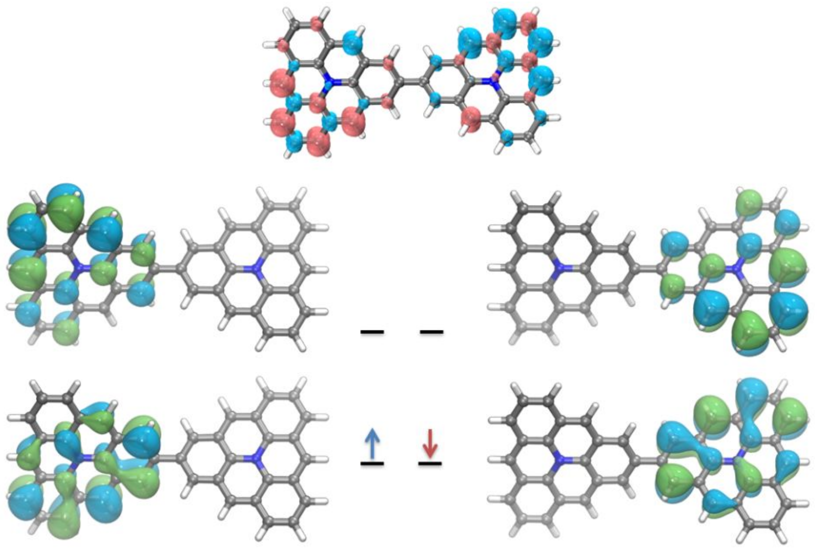

Figure S9. Spin density distribution and frontier molecular orbital of TRI(N) dimer for the BS states.

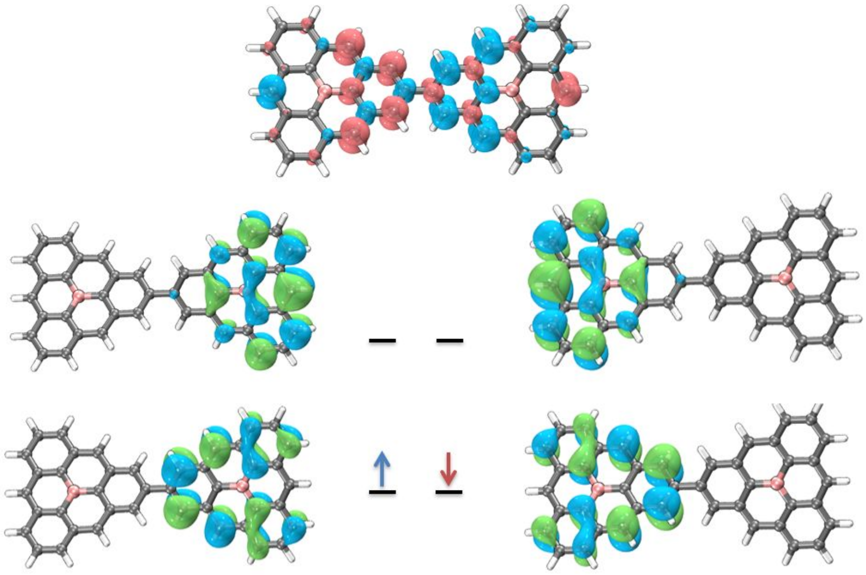

Figure S10. Spin density distribution and frontier molecular orbital of TRI(B) dimer for the BS states.

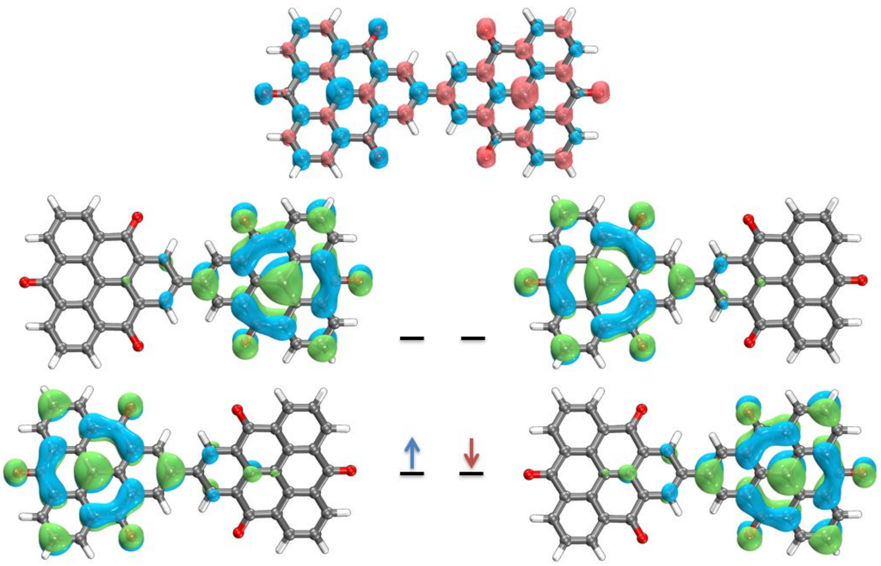

Figure S11. Spin density distribution and frontier molecular orbital of TOT dimer for the BS states.

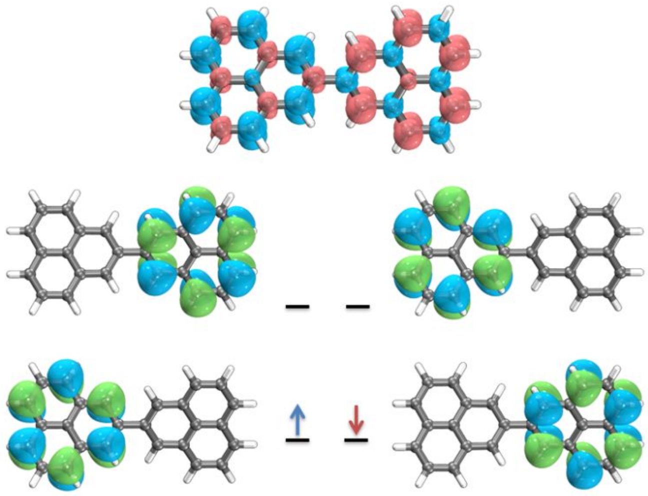

Figure S12. Spin density distribution and frontier molecular orbital of PLY dimer for the BS states.

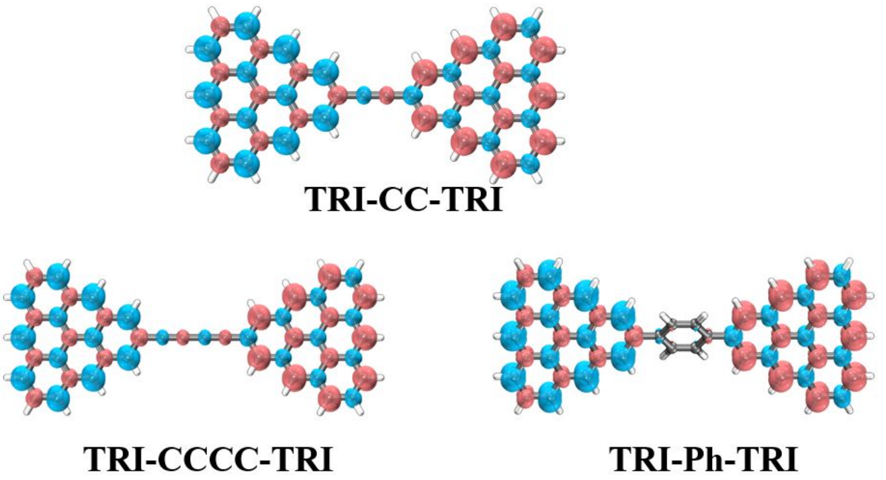

Figure S13 . Spin density distribution of TRI-CC-TRI, TRI -CCCC-TRI and TRI -Ph-TRI for the BS states.

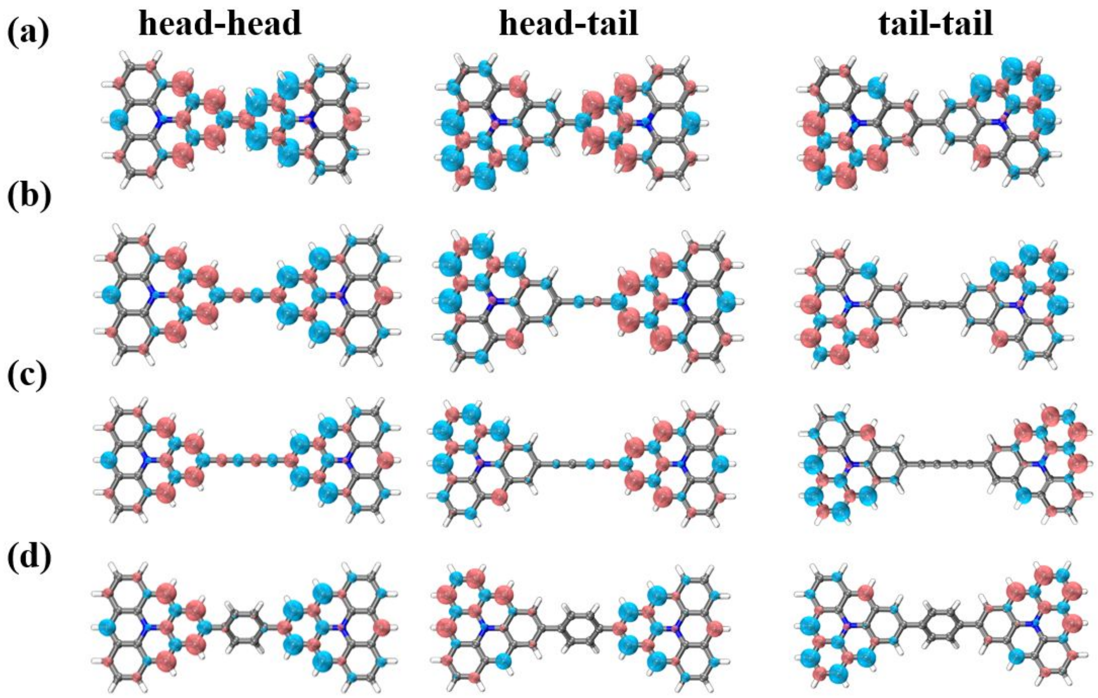

Figure S14. Spin density distributions of TRI(N)-TRI(N) (a), TRI(N)-CC-TRI(N) (b), TRI(N)-CCCC-TRI(N) (c) and TRI(N)-Ph-TRI(N) (d) for the BS states.

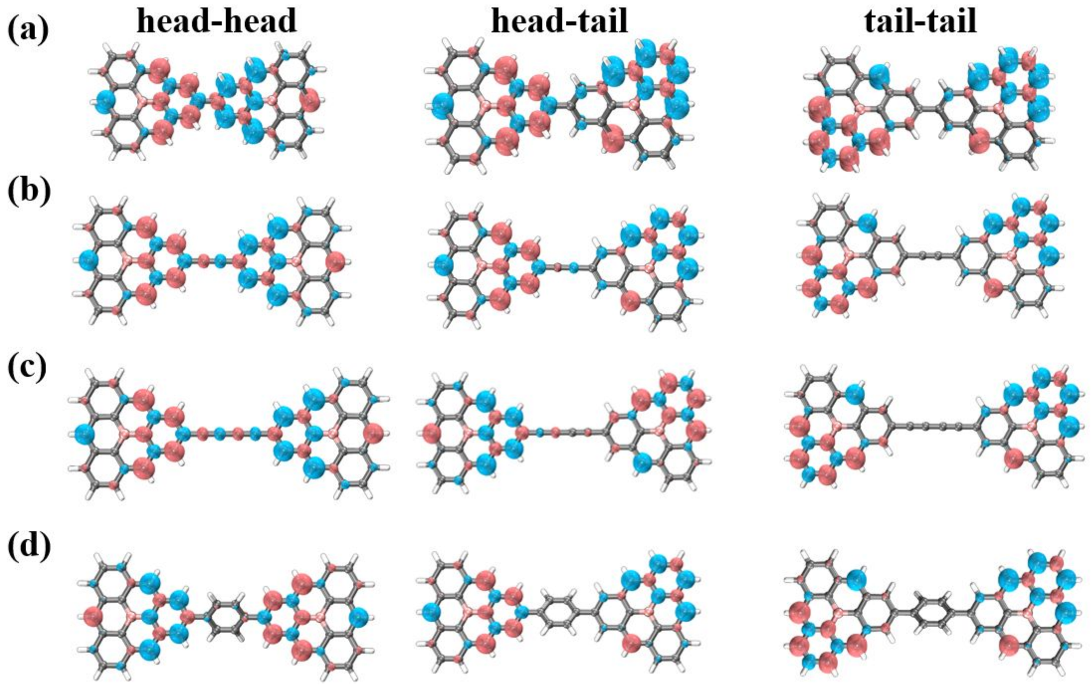

Figure S15. Spin density distributions of TRI(B)-TRI(B) (a), TRI(B)-CC-TRI(B) (b), TRI(B)-CCCC-TRI(B) (c) and TRI(B)-Ph-TRI(B) (d) for the BS states.

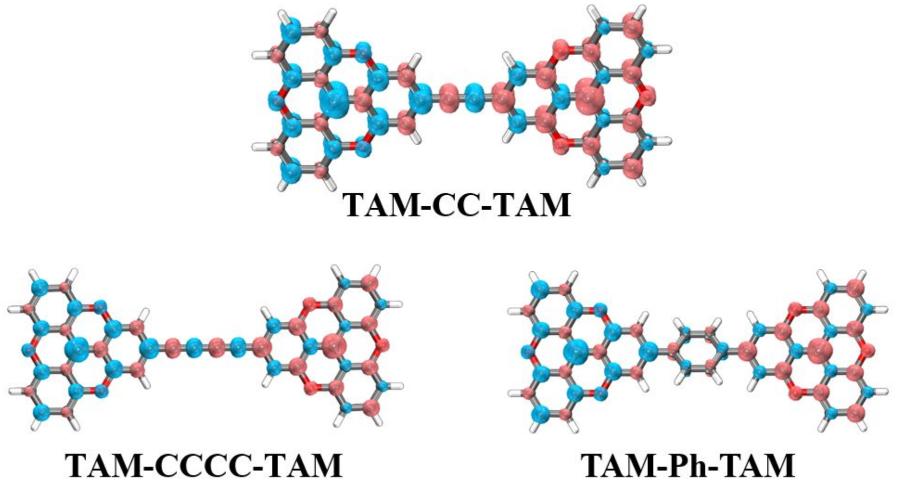

Figure S16. Spin density distributions of TAM-CC-TAM, TAM-CCCC-TAM and TAM-Ph-TAM for the BS states.

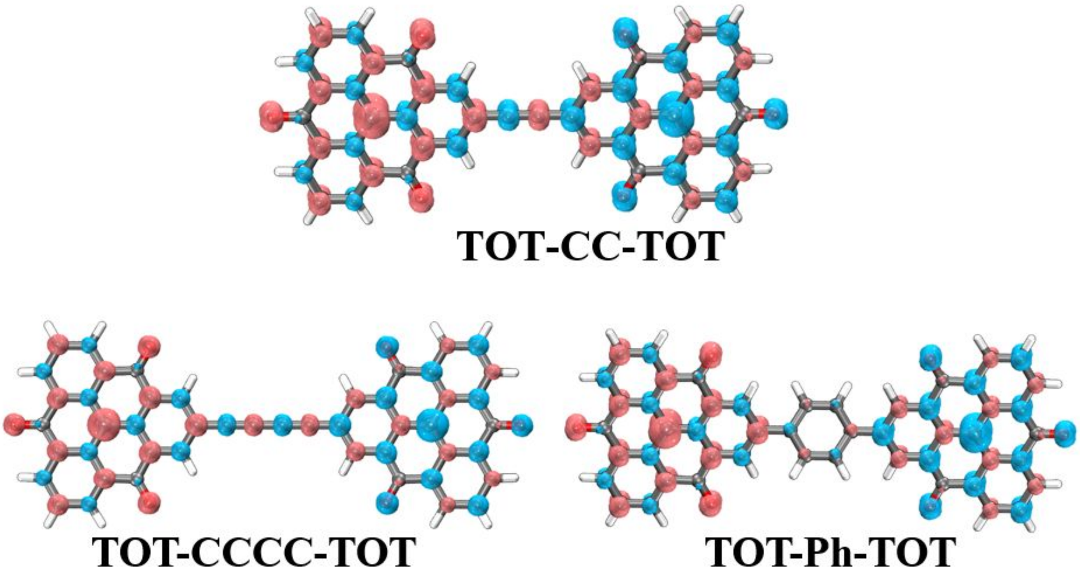

Figure S17. Spin density distributions of TOT-CC-TOT, TOT -CCCC-TOT and TOT-Ph-TOT for the BS states.

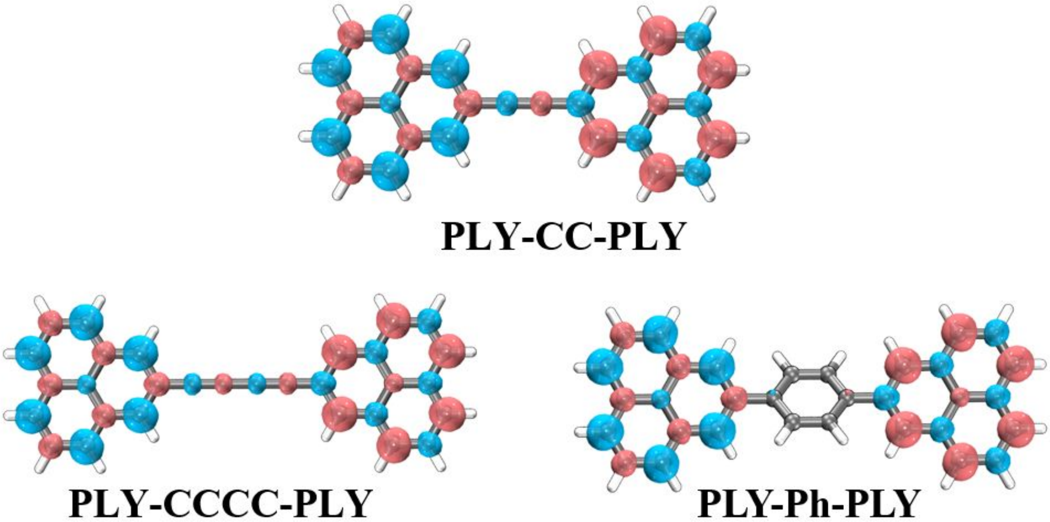

Figure S18. Spin density distributions of PLY-CC-PLY, PLY-CCCC-PLY and PLY-Ph-PLY for the BS states.

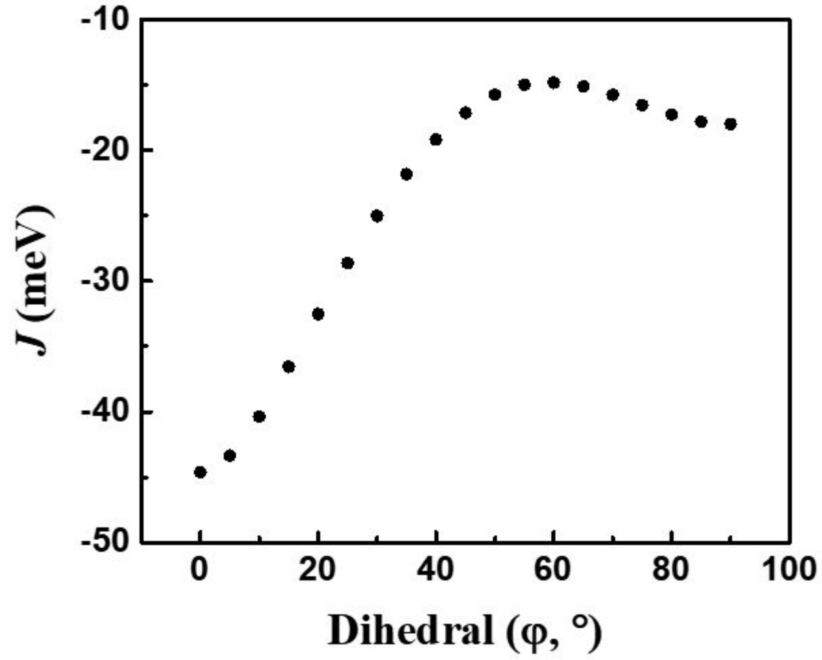

Figure S19. Relationship between magnetic coupling (*J*) and dihedral angle (φ) in PLY dimer.

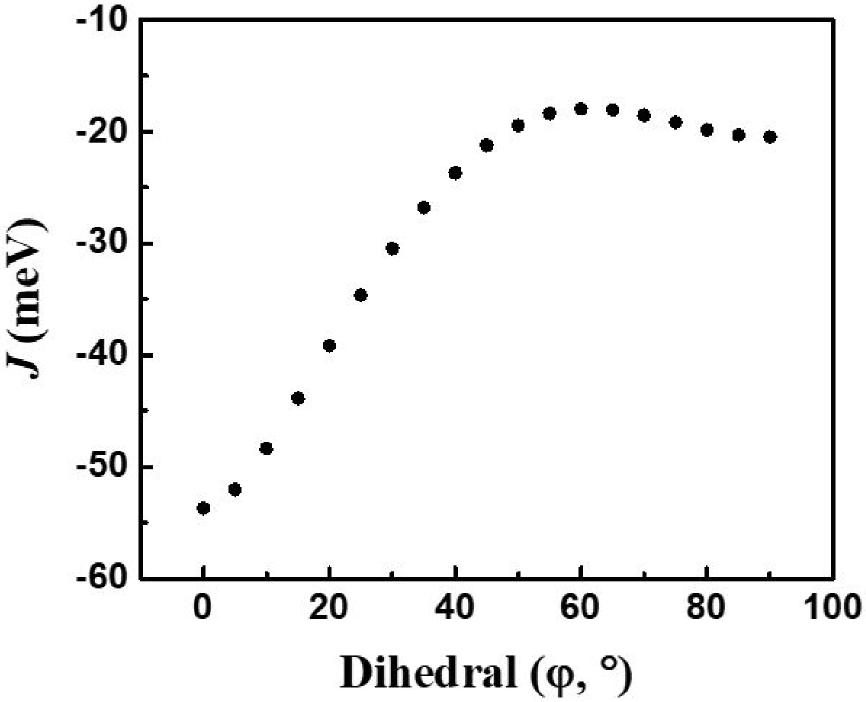

Figure S20. Relationship between magnetic coupling (*J*) and dihedral angle (φ) in TRI(B) dimer.

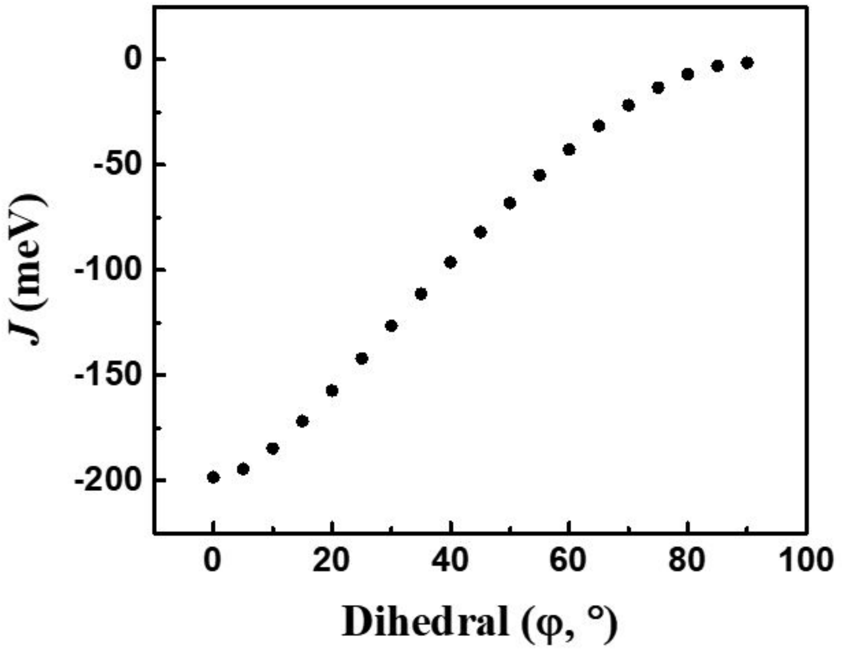

Figure S21. Relationship between magnetic coupling (*J*) and dihedral angle (φ) in TAM dimer.

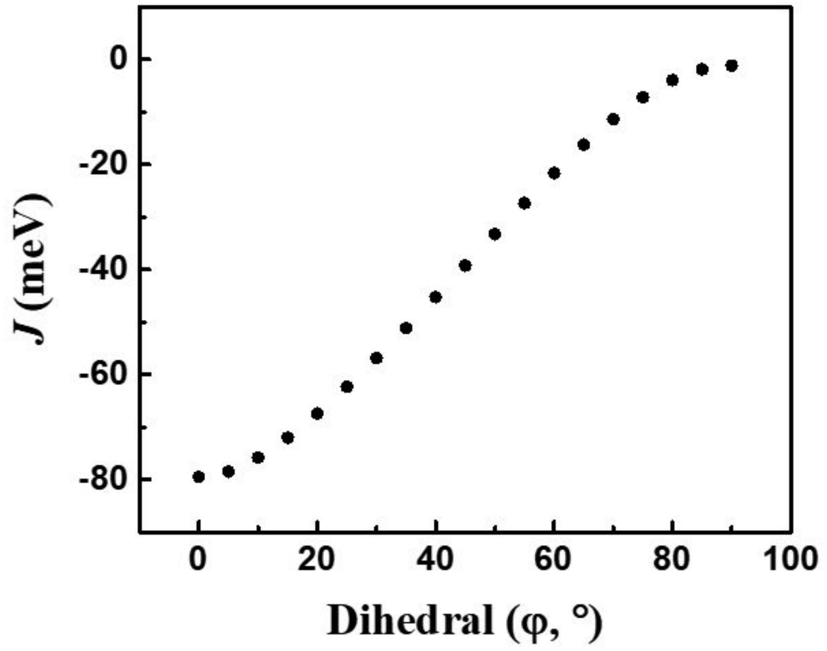

Figure S22. Relationship between magnetic coupling (*J*) and dihedral angle (φ) in TOT dimer.

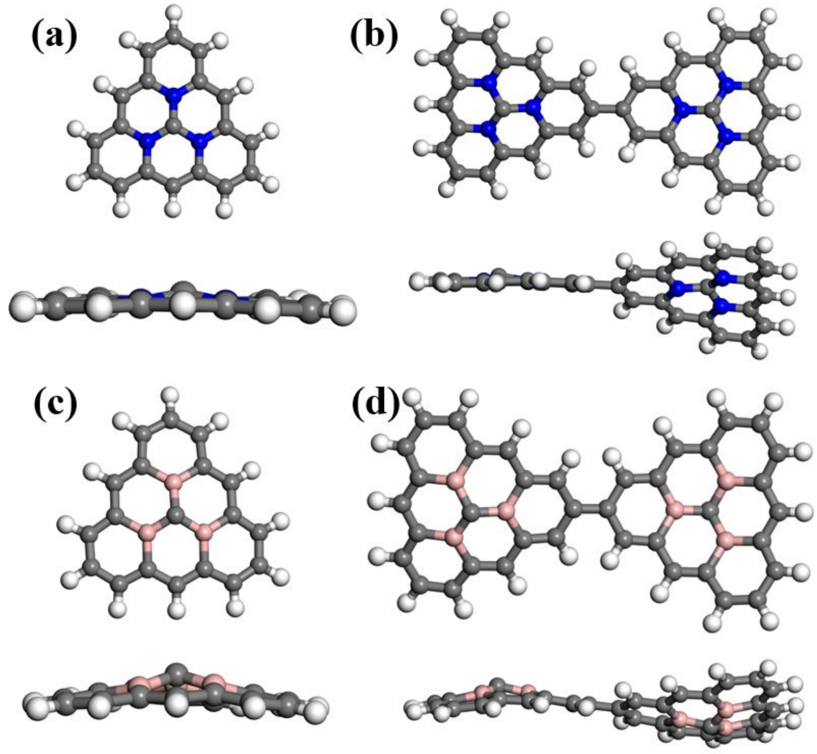

Figure S23. Top and side view of the optimized geometry of Tr3N (a, b) and Tr3B (c, d) for their monomer (a, c) and dimer (b, d).

Table S1. Comparison of TRI(N) and TRI(B) monomer with C2v and D3h symmetry. These energies are calculated at the PBE0/def2-TZVP level.

		E (Hartree)
TRI(N)	C2v	-861.497347902
	D3h	-861.495203128
TRI(B)	C2v	-831.608771830
	D3h	-831.606646245

Table S2. Electromers of TRI(N) dimers with different spin density distributions including head-head, head-tail and tail-tail.

Systems	Electromers	E_{BS}/Hartree (<S^2>)	E_{HS}/Hartree (<S^2>)	*J*/meV
TRI(N)-TRI(N)	head-head	-1721.809965 (1.44)	-1721.808770 (2.40)	-27.04
	head-tail	-1721.810677 (1.39)	-1721.810552 (2.39)	-2.85
	tail-tail	-1721.810669 (1.40)	-1721.810654 (2.43)	-0.33
TRI(N)-CC-TRI(N)	head-head	-1797.897710 (1.47)	-1797.896720 (2.43)	-22.15
	head-tail	-1797.898512 (1.42)	-1797.898414 (2.42)	-2.21
	tail-tail	-1797.899058 (1.41)	-1797.899044 (2.41)	-0.33
TRI(N)-CCCC-TRI(N)	head-head	-1873.988748 (1.49)	-1873.988091 (2.45)	-14.57
	head-tail	-1873.989933 (1.43)	-1873.989877 (2.43)	-1.24
	tail-tail	-1873.990894 (1.41)	-1873.990889 (2.41)	-0.13
TRI(N)-Ph-TRI(N)	head-head	-1952.670539 (1.42)	-1952.670367 (2.42)	-3.89
	head-tail	-1952.671326 (1.40)	-1952.671306 (2.40)	-0.45
	tail-tail	-1952.671669 (1.40)	-1952.671666 (2.40)	-0.07

Table S3. Electromers of TRI(B) dimers with different spin density distributions including head-head, head-tail and tail-tail.

Systems	Electromers	E_{BS}/Hartree (<S^2>)	E_{HS}/Hartree (<S^2>)	J/meV
TRI(B)-	head-head	-1662.032461 (1.78)	-1662.031268 (2.74)	-23.71
TRI(B)	head-tail	-1662.032001 (1.75)	-1662.031964 (2.76)	-0.74
	tail-tail	-1662.031421 (1.78)	-1662.031413 (2.78)	-0.15
TRI(B)-CC-	head-head	-1738.122272 (1.79)	-1738.121120 (2.74)	-22.86
TRI(B)	head-tail	-1738.121398 (1.76)	-1738.121375 (2.77)	-0.45
	tail-tail	-1738.120649 (1.78)	-1738.120646 (2.78)	-0.06
TRI(B)-	head-head	-1814.214234 (1.78)	-1814.213487 (2.74)	-14.83
CCCC-	head-tail	-1814.213202 (1.76)	-1814.213184 (2.77)	-0.36
TRI(B)	tail-tail	-1814.212241 (1.78)	-1814.212239 (2.78)	-0.03
TRI(B)-Ph-	head-head	-1892.892902 (1.76)	-1892.892716 (2.75)	-3.67
TRI(B)	head-tail	-1892.892712 (1.76)	-1892.892708 (2.76)	-0.08
	tail-tail	-1892.892328 (1.77)	-1892.892327 (2.77)	-0.01

Table S4. The magnetic coupling (*J*) and overlap integral (*S*) for TAM-TAM and TOT-TOT at planar (φ = 0°), free molecule (φ = 24° for TAM-TAM and φ = 27° for TOT-TOT), and perpendicular (φ = 90°) configurations

φ (°)		TAM-TAM	TOT-TOT
0	J (meV)	-198.44	-79.47
	S	0.526	0.389
24/27	J (meV)	-144.15	-59.69
	S	0.448	0.344
90	J (meV)	-1.47	-1.12
	S	0.048	0.061

Table S5. Magnetic couplings of planar TRI(N) dimer calculated by PBE0, B3LYP, MN15, M06-2X and ωB97XD functionals. "-sp" indicate single point energy at PBE0 optimized geometry and "-opt" means the geometry is also re-optimized by corresponding functionals.

	J (meV)	$\|t\|$ (eV)	U (eV)	$-4t^2/U$ (eV)	K (eV)
PBE0	1.23	0.230	0.733	-0.289	0.291
B3LYP-sp	1.09	0.159	0.553	-0.183	0.184
B3LYP-opt	1.01	0.159	0.551	-0.184	0.185
MN15-sp	1.36	0.508	0.884	-1.168	1.169
MN15-opt	1.50	0.503	0.905	-1.118	1.120
M06-2X-sp	0.76	0.648	0.907	-1.852	1.853
M06-2X-opt	0.75	0.639	0.933	-1.751	1.751
ωB97XD-sp	2.19	1.245	1.446	-4.288	4.290
ωB97XD-opt	2.39	1.231	1.495	-4.054	4.057

References

(1) Yu, H.; Sun, J.; Heine, T. Predicting Magnetic Coupling and Spin-Polarization Energy in Triangulene Analogues. *J. Chem. Theory Comput.* **2023**, *19* (12), 3486–3497.

(2) Yu, H.; Heine, T. *Optimized Strucures of Triangulene Dimers*, 2023. https://doi.org/10.5281/zenodo.7915258.

(3) Ruiz, E.; Alvarez, S.; Cano, J.; Polo, V. About the calculation of exchange coupling constants using density-functional theory: The role of the self-interaction error. *J. Chem. Phys.* **2005**, *123* (16), 164110.

(4) Anindya, K. N.; Rochefort, A. Controlling the magnetic properties of two-dimensional carbon-based Kagome polymers. *Carbon Trends* **2022**, *7*, 100170.

(5) Mori, T. *Electronic properties of organic conductors*; Springer, 2016.

(6) Guo, Y.; Riplinger, C.; Becker, U.; Liakos, D. G.; Minenkov, Y.; Cavallo, L.; Neese, F. Communication: An improved linear scaling perturbative triples correction for the domain based local pair-natural orbital based singles and doubles coupled cluster method [DLPNO-CCSD (T)]. *J. Chem. Phys.* **2018**, *148* (1), 11101.

(7) Neese, F. The ORCA program system. *Wiley Interdiscip. Rev. Comput. Mol. Sci.* **2012**, *2* (1), 73–78.

(8) Weigend, F. Accurate Coulomb-fitting basis sets for H to Rn. *Phys. Chem. Chem. Phys.* **2006**, *8* (9), 1057–1065.

(9) Ni, X.; Huang, H.; Brédas, J.-L. Organic Higher-Order Topological Insulators: Heterotriangulene-Based Covalent Organic Frameworks. *J. Am. Chem. Soc.* **2022**, *144* (49), 22778–22786.

(10) Valeev, E. F.; Coropceanu, V.; Da Silva Filho, D. A.; Salman, S.; Brédas, J.-L. Effect of electronic polarization on charge-transport parameters in molecular organic semiconductors. *J. Am. Chem. Soc.* **2006**, *128* (30), 9882–9886.

(11) Lu, T.; Chen, F. Multiwfn: A multifunctional wavefunction analyzer. *J. Comput. Chem.* **2012**, *33* (5), 580–592.

Appendix:

Optimized geometry at the PBE0/def2-TZVP level.

TRI-TRI

C	10.03151574	2.49552918	14.79159305
C	5.18853737	6.29361245	14.38740101
C	7.94095059	-1.16376408	15.36282010
C	6.92515850	9.74272070	16.16971518
C	12.15953685	-1.19216309	14.78742107
C	3.06726022	9.98534873	14.38486705
C	7.05619951	7.37087550	15.57033511
C	7.92765755	1.27825709	15.16551007
C	3.20133623	7.59522554	13.78790701

C	12.14227588	1.26741109	14.59520606
C	4.92363936	11.05586081	15.57297813
C	10.06493371	-2.40427117	15.17092011
C	9.29985564	3.70111826	14.79780305
C	5.91513345	5.08493937	14.40345704
C	7.27404655	-2.39434317	15.55340911
C	7.47529556	10.90382178	16.75673822
C	13.55855497	-1.16774508	14.59307607
C	1.78976313	10.03314074	13.78375397
C	7.73855056	6.13680945	15.55417313
C	7.24696950	2.51347318	15.15890509
C	1.92091114	7.70075656	13.20071796
C	13.54157897	1.23255109	14.40511506
C	5.52220939	12.18551088	16.17400315
C	9.34673366	-3.60504426	15.36558410
C	9.33730668	1.26872409	14.97866208
C	5.76555240	7.45091954	14.97885507
C	9.35131369	-1.17294408	15.17025308
C	5.63541443	9.82349770	15.57278610
C	11.44746981	0.04005600	14.78777408
C	3.77731727	8.75198764	14.38493801
C	7.91921955	3.71953227	14.97838807
C	7.18034952	4.99826036	14.97865805
C	7.97532959	-3.58790526	15.55278214
C	6.77662051	12.09887785	16.75283220
C	14.22704803	0.02984300	14.40603101
C	1.23655109	8.90408964	13.20470394
C	11.42022480	2.46885318	14.60064008
C	3.91866128	6.39098048	13.80113501
C	7.25895351	0.06090700	15.35688612
C	7.60807952	8.51868964	16.15653819
C	11.45331685	-2.38760717	14.97919210
C	3.65524726	11.11049878	14.97897408
C	10.04541971	0.04516000	14.97888809
C	5.05936936	8.67557662	14.97887010
H	9.83183870	4.63046933	14.62656505
H	5.48105739	4.21338630	13.92592903
H	6.19968544	-2.39063617	15.70095314
H	8.45750558	10.84527478	17.21266422
H	14.10383101	-2.10514015	14.59234104
H	1.24623809	10.97155377	13.78219099
H	8.71030763	6.07623146	16.03171316
H	6.17607943	2.51550918	15.32992512
H	1.47960410	6.82140648	12.74462094

H	14.07367600	2.16591516	14.25768803
H	4.98220836	13.12595395	16.17556915
H	9.88781274	-4.54486933	15.36654609
H	8.59146061	8.45841058	16.61205719
H	6.18352643	0.06639900	15.50384409
H	3.11298122	12.05079289	14.97898006
H	11.99699689	-3.32708424	14.97929809
H	3.47823325	5.50972938	13.34551298
H	11.95125888	3.40403925	14.45367602
H	7.44360451	-4.52112333	15.70085313
H	7.21754052	12.97813691	17.20894722
H	15.30116111	0.02589200	14.25832902
H	0.25475802	8.96292362	12.74844292

TRI-CC-TRI

C	-4.12938330	-1.21656709	0.00288600
C	4.12861830	1.21812109	-0.00073500
C	-6.24076743	2.47931118	0.00859100
C	6.24005045	-2.47773018	0.00210300
C	-8.38621160	-1.19637208	0.00492300
C	8.38544958	1.19797808	-0.00280500
C	4.12208830	-1.23916309	0.00180900
C	-4.12280330	1.24071609	0.00598300
C	6.25360844	2.44518217	-0.00306800
C	-6.25437147	-2.44360118	0.00231300
C	8.37892858	-1.24217709	-0.00022900
C	-8.37964562	1.24378109	0.00804700
C	-2.72991319	-1.19814109	0.00226400
C	2.72916120	1.19967009	-0.00002900
C	-6.97380450	3.68615026	0.01049300
C	6.97311652	-3.68457926	0.00302800
C	-9.07709864	-2.43984618	0.00365600
C	9.07632366	2.44144618	-0.00445700
C	2.72253020	-1.21321609	0.00243800
C	-2.72325820	1.21474409	0.00527400
C	6.99299850	3.64770126	-0.00470000
C	-6.99378951	-3.64612826	0.00111800
C	9.06296765	-2.48961918	0.00075800
C	-9.06367267	2.49121818	0.00997700
C	-4.83668835	0.01409700	0.00477600
C	4.83594835	-0.01254600	0.00019600
C	-6.96043849	1.24184609	0.00735900
C	6.95970748	-1.24025709	0.00045500
C	-6.96723852	-1.20222609	0.00423900
C	6.96649048	1.20381608	-0.00211500

C	-2.02466015	0.00648700	0.00343700
C	-8.36283662	3.67783926	0.01115800
C	8.36214059	-3.67625027	0.00235200
C	-8.38286863	-3.63024626	0.00179600
C	8.38208460	3.63183726	-0.00537000
C	-4.86067935	-2.42705017	0.00168400
C	4.85989735	2.42861117	-0.00235900
C	-4.84750135	2.45519418	0.00787900
C	4.84680335	-2.45363418	0.00273800
C	-9.06164767	0.02572700	0.00681400
C	9.06090867	-0.02413500	-0.00184700
C	-6.25783546	0.01796700	0.00545900
C	6.25709546	-0.01639600	-0.00048900
H	-2.18307916	-2.13366215	0.00082600
H	2.18230716	2.13518116	-0.00073400
H	-6.43336149	4.62621633	0.01143400
H	6.43268044	-4.62464933	0.00428100
H	-10.16134874	-2.43474617	0.00417900
H	10.16057371	2.43635617	-0.00498500
H	2.17072516	-2.14581315	0.00366700
H	-2.17143216	2.14733016	0.00619000
H	6.45775349	4.59074333	-0.00542900
H	-6.45855244	-4.58917433	-0.00034600
H	10.14722474	-2.49049118	0.00023600
H	-10.14792971	2.49210018	0.01050400
H	4.29961631	-3.39071425	0.00398200
H	-4.30030431	3.39226924	0.00880800
H	10.14651172	-0.02705300	-0.00237300
H	-10.14725173	0.02866300	0.00733900
H	4.31785231	3.36867024	-0.00307600
H	-4.31864331	-3.36711324	0.00023000
H	-8.90149265	4.61880833	0.01262500
H	8.90080964	-4.61721333	0.00308800
H	-8.92665064	-4.56825933	0.00084900
H	8.92585465	4.56985733	-0.00662700
C	2.02392015	-0.00498000	0.00153700
C	0.60377204	-0.00101800	0.00220600
C	-0.60451204	0.00251400	0.00277100

TRI-CCCC-TRI

C	-3.70435627	-1.20467509	-0.12056701
C	7.11224150	1.23061409	-0.06200900
C	-5.82529344	2.46465218	0.28040202
C	9.22813869	-2.44199318	0.33556202
C	-7.96057655	-1.19286108	-0.13972201

C	11.36845782	1.21727409	-0.04152400
C	7.10866453	-1.21443609	0.19520401
C	-3.70409327	1.23870709	0.15209501
C	9.23540164	2.45405318	-0.18207201
C	-5.82580139	-2.42801718	-0.26818602
C	11.36489180	-1.20936408	0.21544502
C	-7.96032959	1.23210909	0.13260001
C	-2.30512916	-1.18448709	-0.11132701
C	5.71299742	1.21089509	-0.06599900
C	-6.56168147	3.66205026	0.41171903
C	9.96289473	-3.64050526	0.46617003
C	-8.64855963	-2.42985717	-0.28194902
C	12.05815189	2.45486518	-0.16949601
C	5.70944939	-1.19201709	0.18582201
C	-2.30485817	1.21678609	0.15567501
C	9.97366169	3.65108026	-0.30562302
C	-6.56242846	-3.62450826	-0.40619403
C	12.05091185	-2.44833117	0.35001003
C	-8.64806062	2.46994918	0.26858902
C	-4.41433432	0.01745500	0.01252900
C	7.82056054	0.00738300	0.06999400
C	-6.54131545	1.23324409	0.13909401
C	9.94586073	-1.20998909	0.20871001
C	-6.54156749	-1.19573209	-0.13336001
C	9.94947570	1.22065909	-0.04837300
C	-1.60455511	0.01571400	0.02540200
C	-7.95071856	3.65074926	0.40438003
C	11.35193781	-3.62968826	0.47183903
C	-7.95146158	-3.61150726	-0.41144103
C	11.36269381	3.63757226	-0.29799502
C	-4.43243132	-2.40921917	-0.25910002
C	7.84197455	2.43572717	-0.18611301
C	-4.43192432	2.44414618	0.28397902
C	7.83483557	-2.42097018	0.32627202
C	-8.63905360	0.02004100	-0.00663200
C	12.04527489	0.00326400	0.09021101
C	-5.83549343	0.01832500	0.00606900
C	9.24171769	0.00600400	0.07679301
H	-1.75504413	-2.11250415	-0.21226102
H	5.16417437	2.13973615	-0.16622001
H	-6.02386942	4.59745733	0.51918004
H	9.42379668	-4.57636133	0.56270004
H	-9.73278772	-2.42674317	-0.28675602
H	13.14237892	2.45134618	-0.16420601

H	5.15791237	-2.11980215	0.28079002
H	-1.75457313	2.14412216	0.26166602
H	9.43734468	4.58799033	-0.40729903
H	-6.02480845	-4.56057133	-0.50879104
H	13.13514295	-2.44693018	0.35510303
H	-9.73229072	2.46816118	0.26357402
H	7.28875953	-3.35370624	0.42216703
H	-3.88714228	3.37644524	0.39085503
H	13.13086492	0.00221700	0.09540801
H	-9.72464570	0.02070500	-0.01154800
H	7.29866551	3.36952824	-0.28721702
H	-3.88783928	-3.34218324	-0.36102703
H	-8.49191459	4.58460433	0.50696404
H	11.89184587	-4.56438533	0.57355504
H	-8.49284859	-4.54469833	-0.51894104
H	11.90536286	4.57121933	-0.39452503
C	5.01078136	0.01009900	0.05656900
C	3.59406026	0.01146400	0.04981800
C	2.38096417	0.01258600	0.04406300
C	-0.18782801	0.01475500	0.03197900
C	1.02526807	0.01377000	0.03766500

TRI-Ph-TRI

C	-5.84713740	-1.15513408	-0.36159203
C	4.16813030	1.18022009	0.36695403
C	-7.96289456	2.36772817	0.73466805
C	6.28350146	-2.34297717	-0.72871805
C	-10.10489871	-1.14772008	-0.35241403
C	8.42592360	1.17246108	0.35764003
C	4.16485430	-1.16629808	-0.36891003
C	-5.84413241	1.19126508	0.37476703
C	6.29003944	2.35197917	0.72357005
C	-7.96897158	-2.32695117	-0.71847405
C	8.42266762	-1.16842509	-0.36596703
C	-10.10198373	1.19306209	0.37160303
C	-4.43731732	-1.12751808	-0.35428003
C	2.75830320	1.15281508	0.35970803
C	-8.69261464	3.52361525	1.09140108
C	7.01310850	-3.49895325	-1.08529708
C	-10.78613276	-2.33026217	-0.71736205
C	9.10721766	2.35503517	0.72237605
C	2.75510820	-1.13563308	-0.35952703
C	-4.43436632	1.16078208	0.36546303
C	7.02286150	3.50626025	1.07904908
C	-8.70165664	-3.48124825	-1.07417308

C	9.10066364	-2.35257917	-0.73172005
C	-10.78003876	2.37712717	0.73753105
C	-6.55978850	0.01886600	0.00710500
C	4.88065635	0.00613100	-0.00151000
C	-8.67875660	1.19214008	0.37132503
C	6.99949050	-1.16738008	-0.36562103
C	-8.68176662	-1.14983308	-0.35413303
C	7.00275150	1.17472008	0.35941603
C	-3.72853627	0.01591900	0.00508200
C	-10.07684670	3.51642925	1.08924308
C	8.39736761	-3.49188625	-1.08321408
C	-10.08591971	-3.47110425	-1.07006907
C	8.40709759	3.49597725	1.07490608
C	-6.56755449	-2.30535417	-0.71409405
C	4.88866035	2.33049617	0.71921805
C	-6.56156849	2.34311317	0.72831005
C	4.88218235	-2.31823917	-0.72227205
C	-10.78441976	0.02342700	0.01008000
C	9.10525865	0.00122700	-0.00466900
C	-7.97359559	0.02042300	0.00810800
C	6.29447045	0.00449000	-0.00257300
H	-3.90015128	-2.03475215	-0.60835705
H	2.22134316	2.06022315	0.61360104
H	-8.15004561	4.42081332	1.36835710
H	6.47055249	-4.39620632	-1.36210710
H	-11.87058983	-2.33152417	-0.71705405
H	10.19167774	2.35621517	0.72203705
H	2.21566816	-2.04179115	-0.61263804
H	-3.89511228	2.06698015	0.61883105
H	6.48281046	4.40477632	1.35665810
H	-8.16157458	-4.37969532	-1.35193110
H	10.18511674	-2.35628217	-0.73299705
H	-11.86449286	2.38075917	0.73876705
H	4.33749831	-3.21528523	-0.99943607
H	-6.01690942	3.24011023	1.00565607
H	10.19071972	-0.00002800	-0.00547600
H	-11.86987687	0.02439200	0.01077800
H	4.34648031	3.22880723	0.99719107
H	-6.02540144	-3.20362423	-0.99225607
H	-10.61718975	4.41459632	1.36658010
H	8.93757365	-4.39016131	-1.36044510
H	-10.62849975	-4.36814331	-1.34665509
H	8.94979965	4.39299732	1.35132410
C	2.04937915	0.00940100	0.00064800

C	-2.25290116	0.01427000	0.00393300
C	-1.52954311	0.70168505	0.97897407
C	-1.53258011	-0.67478205	-0.97221607
C	-0.14658501	0.70013705	0.97791507
H	-2.05990015	1.21763309	1.77161013
C	-0.14963101	-0.67635405	-0.97326307
H	-2.06531115	-1.18954708	-1.76403113
C	0.57374104	0.01110500	0.00175800
H	0.38615103	1.21489409	1.76972713
H	0.38073903	-1.19235308	-1.76585413

TRI(N)-TRI(N) (head-head)

C	10.026340	2.528635	14.732628
C	5.128384	-10.696507	17.447997
C	7.966135	-1.105797	15.352279
C	7.192713	-7.068374	16.805126
C	12.161103	-1.121596	14.853053
C	3.338847	-7.618077	15.164044
C	7.117198	-9.286535	17.783677
C	7.923772	1.307470	15.111630
C	3.258744	-9.871107	16.150602
C	12.123471	1.326336	14.603973
C	5.298874	-6.229992	15.503647
C	10.086451	-2.319670	15.232916
C	9.269994	3.724734	14.700683
C	5.766729	-11.697124	18.219379
C	7.307382	-2.340830	15.559467
C	7.855740	-5.840317	16.571838
C	13.549318	-1.102434	14.682510
C	2.063288	-7.823939	14.628138
C	7.697830	-10.311462	18.537960
C	7.229271	2.520968	15.070692
C	1.970418	-10.021379	15.583723
C	13.528636	1.283073	14.438479
C	6.009694	-5.022408	15.306952
C	9.373073	-3.522852	15.446472
C	9.338919	1.302398	14.941745
C	5.817533	-9.472980	17.228958
C	9.373230	-1.096258	15.186643
C	5.898284	-7.264837	16.263466
C	11.432065	0.102564	14.814310
C	3.951072	-8.648720	15.935113
C	7.907465	3.713298	14.866367
C	7.018657	-11.502343	18.747407
C	14.216598	0.095955	14.477657

C	1.394430	-9.019705	14.842824
C	11.408546	2.520302	14.566847
C	3.859305	-10.875626	16.904476
C	7.259982	0.084883	15.316566
C	7.784789	-8.070078	17.555908
C	11.460936	-2.322696	15.064010
C	4.037562	-6.414497	14.962583
H	9.801613	4.655157	14.540702
H	5.234683	-12.626891	18.381720
H	6.234926	-2.317155	15.713174
H	8.831936	-5.704374	17.022294
H	14.086721	-2.042205	14.714653
H	1.613846	-7.029873	14.044640
H	8.685850	-10.152039	18.951977
H	6.154311	2.505592	15.201952
H	1.449355	-10.956155	15.752782
H	14.048190	2.220336	14.278805
H	5.540918	-4.258384	14.697962
H	9.938597	-4.447272	15.448732
H	8.773287	-7.919403	17.973940
H	6.184091	0.079063	15.446966
H	3.579763	-5.625141	14.377839
H	12.007055	-3.258303	15.095253
H	3.332833	-11.807799	17.070097
H	11.934062	3.454021	14.407004
H	15.293178	0.094842	14.347242
H	0.405960	-9.164516	14.420935
N	5.222725	-8.461477	16.475656
N	10.047664	0.102162	14.980950
H	7.355279	4.646177	14.837170
H	7.481764	-12.287731	19.334541
C	7.994393	-3.546325	15.609499
C	7.278833	-4.817268	15.831420

TRI(N)-TRI(N) (head-tail)

C	12.071209	-1.194389	14.769603
C	5.169386	6.314660	14.376700
C	9.986921	2.479925	14.775446
C	6.927029	9.717652	16.148599
C	7.883711	-1.134981	15.369289
C	3.101652	10.005675	14.371131
C	7.047930	7.370429	15.554378
C	12.072814	1.258819	14.569694
C	3.221081	7.620508	13.780394
C	9.997683	-2.379101	15.166655

C	4.949824	11.041929	15.552448
C	7.890559	1.282260	15.164686
C	13.471243	-1.164377	14.564140
C	5.896454	5.103673	14.400768
C	9.257692	3.692494	14.783411
C	7.485186	10.879792	16.740263
C	7.208579	-2.344692	15.567344
C	1.837725	10.075538	13.775681
C	7.722803	6.145895	15.540544
C	13.457806	1.226200	14.371262
C	1.939754	7.752262	13.192180
C	9.260837	-3.570151	15.371660
C	5.557777	12.168597	16.157402
C	7.215968	2.525911	15.158017
C	11.364005	0.038899	14.771454
C	5.755001	7.469015	14.965502
C	9.293838	1.259498	14.969548
C	5.644515	9.805374	15.550326
C	9.294438	-1.143744	15.166421
C	3.807984	8.768252	14.377243
C	14.139804	0.019147	14.370471
C	7.151372	5.011722	14.968626
C	7.883005	3.728496	14.969942
C	6.806777	12.080606	16.740829
C	7.902010	-3.545331	15.566499
C	1.271534	8.951194	13.193490
C	11.375404	-2.383772	14.967633
C	3.910449	6.411748	13.789940
C	11.357441	2.468750	14.576320
C	7.610111	8.514453	16.146060
C	7.204458	0.095600	15.364072
C	3.697013	11.129870	14.967757
H	14.002882	-2.108463	14.564000
H	5.450460	4.241741	13.919044
H	9.805332	4.610939	14.607577
H	8.465735	10.796519	17.193994
H	6.136667	-2.318736	15.720149
H	1.315874	11.024543	13.780872
H	8.689866	6.089664	16.024835
H	13.979787	2.162909	14.219112
H	1.499141	6.872690	12.738131
H	9.804129	-4.507591	15.370424
H	5.013196	13.105349	16.150010
H	6.146961	2.521061	15.335531

H	8.590335	8.450231	16.603843
H	11.888147	3.401368	14.423939
H	3.166847	12.075171	14.968890
H	6.131635	0.111839	15.517075
H	3.462288	5.536595	13.335208
H	11.912744	-3.324474	14.966935
H	7.253708	12.955255	17.198839
H	7.365058	-4.474819	15.721267
H	0.291242	9.020123	12.735050
N	5.069816	8.681472	14.964745
N	9.983671	0.052312	14.969164
H	15.213147	0.009794	14.215241

TRI(N)-TRI(N) (tail-tail)

C	10.03163073	2.50073418	14.79126306
C	5.18845637	6.28853346	14.38599202
C	7.95351558	-1.12410608	15.37677509
C	6.91019348	9.70150372	16.17197418
C	12.13908685	-1.16777808	14.77412306
C	3.09145322	9.96324473	14.37411205
C	7.05090150	7.35476153	15.57542013
C	7.93059655	1.29292709	15.17469510
C	3.23182223	7.57895154	13.78032501
C	12.11484990	1.28566309	14.58211802
C	4.92577135	11.01118777	15.56634712
C	10.06378672	-2.35909617	15.17186310
C	9.28731769	3.70151227	14.80451805
C	5.92479241	5.08295436	14.41329302
C	7.28700551	-2.35933017	15.57606112
C	7.45743853	10.86567080	16.76774823
C	13.52348497	-1.15505408	14.57185606
C	1.83000413	10.02362274	13.77183298
C	7.73440456	6.13669546	15.56435413
C	7.24763854	2.51147218	15.16969909
C	1.95303814	7.70138755	13.18563996
C	13.51530897	1.23559209	14.38174405
C	5.52396139	12.14180989	16.17675816
C	9.34332666	-3.56250426	15.37533710
C	9.34132869	1.27373309	14.98135808
C	5.76092940	7.44556956	14.97918707
C	9.35716967	-1.12968408	15.17382212
C	5.62997538	9.78041870	15.56675713
C	11.41901685	0.06182100	14.78026506
C	3.80728227	8.73120362	14.38238304
C	7.91939455	3.71918227	14.98680506

C	7.17733152	4.99695436	14.98649009
C	7.97724760	-3.55454326	15.57399310
C	6.76975550	12.06233186	16.76615821
C	14.19473604	0.04271800	14.37802103
C	1.27614409	8.89571562	13.18542093
C	11.40975381	2.48574518	14.59135206
C	3.93169028	6.37579246	13.79233998
C	7.25951052	0.07354101	15.37610213
C	7.60221354	8.50277559	16.17196815
C	11.43388080	-2.36742717	14.97338909
C	3.67620027	11.09120578	14.97578510
H	9.82394671	4.62625833	14.62918204
H	5.48732439	4.21682630	13.93125398
H	6.21461343	-2.33810117	15.73036312
H	8.43626362	10.78871478	17.22629222
H	14.05398003	-2.09929015	14.57052005
H	1.30066409	10.96850481	13.77527999
H	8.70039164	6.08591646	16.05125917
H	6.17975742	2.50203618	15.34958612
H	1.52159111	6.81916748	12.72794090
H	14.03832903	2.17234716	14.23045101
H	4.97270636	13.07471594	16.16773415
H	9.89815572	-4.49332232	15.37089111
H	8.58051961	8.44472558	16.63471519
H	6.18686443	0.07539501	15.53106910
H	3.13927523	12.03268185	14.97544007
H	11.97267384	-3.30783324	14.97132006
H	3.49358925	5.49735838	13.33405798
H	11.93935186	3.41872125	14.44075404
H	7.44662654	-4.48692232	15.72815211
H	7.20795749	12.93972891	17.22746424
H	15.26793708	0.03691000	14.22191300
H	0.29770102	8.95789162	12.72179392
N	5.06648936	8.65300065	14.97643609
N	10.03868371	0.06796301	14.97853507

TRI(N)-CC-TRI(N) (head-head)

C	-4.14695430	-1.21300509	0.00430600
C	4.14621430	1.21457609	0.00066800
C	-6.23276643	2.46414618	0.00571800
C	6.23202643	-2.46257618	-0.00075600
C	-8.36983959	-1.18641509	0.00634700
C	8.36910060	1.18798509	-0.00137300
C	4.13951930	-1.23581509	0.00038800
C	-4.14025930	1.23738609	0.00457800

C	6.24538445	2.42989017	-0.00019800
C	-6.24612344	-2.42831917	0.00517600
C	8.36249361	-1.23229009	-0.00164800
C	-8.36323361	1.23385909	0.00661400
C	-2.73432220	-1.19053809	0.00363500
C	2.73358220	1.19210909	0.00133900
C	-6.94484951	3.66791326	0.00619300
C	6.94410850	-3.66634326	-0.00123500
C	-9.04265963	-2.43199418	0.00653200
C	9.04192063	2.43356318	-0.00155300
C	2.72702519	-1.20562009	0.00106400
C	-2.72776520	1.20719109	0.00390200
C	6.96402753	3.62975026	-0.00040400
C	-6.96476652	-3.62818026	0.00538600
C	9.02850265	-2.48152218	-0.00211200
C	-9.02924265	2.48309218	0.00707400
C	-4.85780135	0.01414100	0.00478500
C	4.85706135	-0.01257100	0.00018500
C	-6.94224749	1.22789709	0.00592500
C	6.94150749	-1.22632709	-0.00095900
C	-6.94884353	-1.18821409	0.00565700
C	6.94810352	1.18978409	-0.00068300
C	-2.02272215	0.00638900	0.00343400
C	-8.33176058	3.66509726	0.00686400
C	8.33101957	-3.66352726	-0.00190700
C	-8.35164161	-3.61779026	0.00606000
C	8.35090262	3.61936026	-0.00107800
C	-4.84032335	-2.41231218	0.00450000
C	4.83958435	2.41388318	0.00047700
C	-4.82707235	2.44046018	0.00503900
C	4.82633135	-2.43889018	-0.00007700
C	-9.05467967	0.02560000	0.00681600
C	9.05393967	-0.02403100	-0.00184600
H	-2.20314916	-2.13408616	0.00327600
H	2.20241016	2.13565716	0.00170100
H	-6.38841545	4.59707933	0.00602500
H	6.38767444	-4.59550933	-0.00107100
H	-10.12607273	-2.42129518	0.00705900
H	10.12533273	2.42286418	-0.00208000
H	2.19070216	-2.14625015	0.00121100
H	-2.19144216	2.14782115	0.00375200
H	6.41267447	4.56194033	-0.00002700
H	-6.41341346	-4.56037033	0.00501200
H	10.11195672	-2.47674118	-0.00263700

H	-10.11269772	2.47831018	0.00759800
H	4.27779631	-3.37344825	0.00008100
H	-4.27853631	3.37501925	0.00487900
H	10.13720673	-0.02698800	-0.00237200
H	-10.13794673	0.02855700	0.00734200
H	4.29615831	3.35142224	0.00084900
H	-4.29689731	-3.34985124	0.00413200
H	-8.87018266	4.60641233	0.00722800
H	8.86944166	-4.60484233	-0.00227300
H	-8.89519766	-4.55615033	0.00621600
H	8.89445862	4.55772033	-0.00123100
C	2.02198215	-0.00481800	0.00153600
C	0.60389204	-0.00090800	0.00220300
C	-0.60463105	0.00247900	0.00276700
N	6.24730044	-0.01636700	-0.00048500
N	-6.24803944	0.01793700	0.00545500

TRI(N)-CC-TRI(N) (head-tail)

C	11.974243	-1.033035	15.173140
C	3.697292	8.638472	14.746231
C	9.894187	2.642747	15.184126
C	5.797092	12.234986	15.443127
C	7.799988	-0.962791	14.490349
C	1.625459	12.326018	14.760712
C	5.802970	9.821967	15.209294
C	11.974799	1.416223	15.408909
C	1.630943	9.878046	14.524726
C	9.908690	-2.210000	14.718443
C	3.702865	13.492022	15.218061
C	7.803682	1.451603	14.724442
C	13.370244	-1.007760	15.406185
C	4.425144	7.429968	14.748926
C	9.171307	3.856601	15.182487
C	6.469640	13.462490	15.673944
C	7.128549	-2.168953	14.262896
C	0.245271	12.330821	14.532528
C	6.477756	8.599455	15.201740
C	13.355312	1.379291	15.633200
C	0.234622	9.944981	14.300087
C	9.175730	-3.397886	14.482662
C	4.427438	14.685428	15.454059
C	7.126618	2.691516	14.732977
C	11.268300	0.200560	15.175283
C	4.396741	9.855598	14.980006
C	9.203011	1.426392	14.952763

C	4.398037	12.255874	15.213350
C	9.206407	-0.974429	14.721548
C	2.333873	11.089994	14.758384
C	14.036436	0.171507	15.629559
C	5.790317	14.662698	15.677896
C	7.821264	-3.370180	14.261413
C	-0.434338	11.143490	14.305179
C	11.282210	-2.219094	14.944625
C	2.323190	8.671015	14.522355
C	11.261342	2.627182	15.407900
C	6.480425	11.032325	15.439301
C	7.120767	0.267679	14.497184
C	2.336001	13.515627	14.993701
H	13.900582	-1.952564	15.402928
H	3.889541	6.506440	14.570577
H	9.710726	4.778461	15.360560
H	7.538685	13.429580	15.848042
H	6.059945	-2.140105	14.089059
H	-0.275771	13.280271	14.538073
H	7.545384	8.588022	15.377028
H	13.874946	2.313152	15.808873
H	-0.293821	9.015902	14.122692
H	9.718884	-4.335393	14.481958
H	3.880254	15.620682	15.454135
H	6.058124	2.697230	14.557530
H	7.549608	11.018452	15.615039
H	11.789469	3.556788	15.584673
H	1.805332	14.460612	14.997330
H	6.051441	0.286945	14.322553
H	1.787763	7.746476	14.344154
H	11.818854	-3.160141	14.942652
H	6.325709	15.587864	15.856601
H	7.287256	-4.297175	14.083612
H	-1.504254	11.162308	14.129781
N	3.710640	11.067223	14.984115
N	9.891838	0.218807	14.949841
H	15.106551	0.158575	15.805059
C	5.790927	7.405343	14.971926
C	6.494015	6.171113	14.967426
C	7.797528	3.890200	14.959698
C	7.094404	5.122698	14.963873

TRI(N)-CC-TRI(N) (tail-tail)

C	10.346213	1.975129	15.195617
C	4.104893	7.915402	14.765739

C	8.263970	-1.640574	14.550369
C	6.187115	11.530989	15.411687
C	12.442990	-1.698685	15.192695
C	2.008221	11.589272	14.768563
C	6.206132	9.115470	15.198300
C	8.244918	0.774955	14.763618
C	2.027500	9.138979	14.553389
C	12.423690	0.751631	15.407611
C	4.082320	12.772302	15.195901
C	10.368838	-2.881811	14.765874
C	9.610178	3.178569	15.189696
C	4.840888	6.711937	14.771734
C	7.596945	-2.873884	14.336740
C	6.854139	12.764265	15.625523
C	13.825441	-1.692127	15.407397
C	0.625814	11.582773	14.553568
C	6.888410	7.898074	15.194679
C	7.562598	1.992329	14.767320
C	0.628787	9.194698	14.341615
C	13.822452	0.695972	15.619080
C	4.802179	13.972543	15.414847
C	9.648974	-4.082090	14.547158
C	9.653316	0.751718	14.979297
C	4.797781	9.138771	14.982316
C	9.665495	-1.650548	14.766643
C	4.785632	11.541021	15.195149
C	11.726365	-0.467370	15.191261
C	2.724813	10.357939	14.770015
C	8.284202	-4.070046	14.336520
C	6.166914	13.960446	15.625718
C	14.499028	-0.498335	15.617727
C	-0.047758	10.389023	14.342955
C	11.722897	1.953878	15.406302
C	2.728260	7.936711	14.554736
C	7.572735	-0.442305	14.550512
C	6.878313	10.332698	15.411604
C	11.737556	-2.895015	14.976927
C	2.713642	12.785562	14.984603
H	10.141572	4.107051	15.354958
H	4.309492	5.783485	14.606309
H	6.526061	-2.849185	14.172843
H	7.924993	12.739523	15.789609
H	14.352849	-2.638090	15.404995
H	0.098429	12.528749	14.555968

H	7.957782	7.894939	15.359799
H	6.493192	1.995415	14.602413
H	0.104866	8.260668	14.177231
H	14.346385	1.630034	15.783241
H	4.249013	14.904311	15.412187
H	10.202165	-5.013843	14.549799
H	7.949210	10.326970	15.577164
H	6.501807	-0.436619	14.385149
H	2.177000	13.727178	14.985278
H	12.274219	-3.836620	14.976259
H	2.197652	7.006952	14.389581
H	12.253516	2.883669	15.571241
H	7.753085	-5.000166	14.171048
H	6.698029	14.890538	15.791353
H	15.570779	-0.508627	15.782769
H	-1.119472	10.399363	14.177677
N	4.103709	10.345954	14.982672
N	10.347425	-0.455444	14.978889
C	6.208790	6.695864	14.981652
C	8.242222	3.194575	14.980103
C	7.530722	4.423900	14.980970
C	6.920239	5.466510	14.980844

TRI(N)-CCCC-TRI(N) (head-head)

C	-3.72232727	-1.20561909	-0.07287201
C	7.12855752	1.22980309	0.15815401
C	-5.81646041	2.45842218	0.18352301
C	9.22267768	-2.43374018	-0.10533901
C	-7.94475156	-1.18691008	-0.09145401
C	11.35098180	1.21106209	0.17646201
C	7.12748350	-1.21456009	-0.02452200
C	-3.72126227	1.23909209	0.10510401
C	9.22481767	2.44567818	0.25861202
C	-5.81858342	-2.42168517	-0.17108601
C	11.34992381	-1.20248409	-0.00318900
C	-7.94370157	1.22696509	0.08373401
C	-2.31012416	-1.18143908	-0.06448300
C	5.71635442	1.20564809	0.14967401
C	-6.53129347	3.65748127	0.26710102
C	9.93750571	-3.63264326	-0.19116501
C	-8.61486062	-2.43052518	-0.18484601
C	12.02109588	2.45450318	0.27210802
C	5.71529839	-1.18817309	-0.02925700
C	-2.30907817	1.21270809	0.11002401
C	9.94069771	3.64345326	0.35114703

C	-6.53445847	-3.61963026	-0.26142502
C	12.01894388	-2.44698218	-0.09254901
C	-8.61272661	2.47162318	0.17079001
C	-4.43563632	0.01729300	0.01274200
C	7.84186157	0.00705700	0.07016501
C	-6.52288145	1.22385109	0.09039101
C	9.92910269	-1.19935409	-0.00983100
C	-6.52392949	-1.18601409	-0.08464901
C	9.93015972	1.21017709	0.16974501
C	-1.60236611	0.01508300	0.02609300
C	-7.91825555	3.65193526	0.25986702
C	11.32446780	-3.62712026	-0.18387101
C	-7.92141659	-3.61192026	-0.26733502
C	11.32765683	3.63573826	0.35689403
C	-4.41284032	-2.40332817	-0.16319801
C	7.81907355	2.42732917	0.25084702
C	-4.41073432	2.43787418	0.18892801
C	7.81695155	-2.41317317	-0.11076301
C	-8.63247762	0.02056400	-0.00712400
C	12.03870388	0.00374500	0.08987601
H	-1.77589913	-2.12087315	-0.13031801
H	5.18213237	2.14495215	0.21736802
H	-5.97705645	4.58530433	0.33714902
H	9.38326567	-4.56032633	-0.26300802
H	-9.69827870	-2.42178517	-0.18926701
H	13.10451497	2.44575818	0.27641202
H	5.18024937	-2.12663215	-0.10195601
H	-1.77403313	2.15131016	0.18089501
H	9.38727266	4.57201133	0.41777603
H	-5.98103041	-4.54831633	-0.32621702
H	13.10236693	-2.43994818	-0.08666101
H	-9.69615072	2.46457218	0.16494101
H	7.27045454	-3.34607824	-0.18313301
H	-3.86423928	3.37092925	0.25935602
H	13.12194696	0.00289100	0.09497101
H	-9.71572070	0.02140800	-0.01225800
H	7.27339254	3.36109524	0.31808602
H	-3.86715728	-3.33723324	-0.22845702
H	-8.45889663	4.58967433	0.32524202
H	11.86510485	-4.56473833	-0.25097202
H	-8.46287663	-4.54881533	-0.33782902
H	11.86911983	4.57250233	0.42907403
C	5.00859136	0.00929700	0.05690400
C	3.59373226	0.01044700	0.05030300

C	2.38036317	0.01152600	0.04466100
C	-0.18750701	0.01395700	0.03272100
C	1.02586207	0.01285400	0.03837000
N	9.23199565	0.00596200	0.07668401
N	-5.82576943	0.01837500	0.00617000

TRI(N)-CCCC-TRI(N) (head-tail)

C	-5.787860	2.448718	-0.003733
C	7.137439	1.230371	0.143749
C	-7.928435	-1.185034	-0.301489
C	9.235035	-2.431920	-0.101779
C	-3.699947	-1.215333	-0.228451
C	11.359720	1.212151	0.216685
C	7.138877	-1.212893	-0.044892
C	-7.915364	1.234624	-0.128854
C	9.232110	2.446258	0.274329
C	-3.687949	1.238832	-0.052636
C	11.361099	-1.201053	0.030876
C	-5.798745	-2.412873	-0.351042
C	-6.522815	3.660120	0.069502
C	5.725410	1.205671	0.117014
C	-8.614905	-2.399946	-0.400241
C	9.951089	-3.630900	-0.181457
C	-2.290067	-1.183216	-0.201303
C	12.028289	2.455338	0.324167
C	5.726889	-1.186206	-0.067725
C	-8.594801	2.474215	-0.052736
C	9.946798	3.644147	0.379178
C	-2.291806	1.218950	-0.029272
C	12.031137	-2.445318	-0.053031
C	-6.546980	-3.610799	-0.449659
C	-6.497409	1.225217	-0.103941
C	7.852262	0.008192	0.061990
C	-6.504167	-1.182784	-0.275861
C	9.940331	-1.197925	0.005908
C	-4.411800	0.015293	-0.153065
C	9.938882	1.211187	0.191555
C	-7.901453	3.665203	0.044847
C	-7.919274	-3.597511	-0.473100
C	11.337648	-3.625266	-0.156296
C	-1.594933	0.010470	-0.103525
C	11.333337	3.636347	0.402912
C	-4.405065	2.445959	0.020970
C	7.826849	2.427618	0.248261
C	-8.611419	0.040453	-0.226512

C	7.829774	-2.411228	-0.125186
C	-4.407459	-2.410690	-0.326301
C	12.048380	0.004989	0.135916
H	-5.965973	4.586585	0.145718
H	5.190330	2.144842	0.180128
H	-9.697637	-2.381236	-0.418212
H	9.397727	-4.558326	-0.262769
H	-1.751340	-2.120120	-0.258887
H	13.111572	2.446794	0.342528
H	5.192932	-2.124564	-0.149584
H	-9.678083	2.462320	-0.073248
H	9.392353	4.572438	0.440936
H	-1.753244	2.154106	0.047171
H	13.114408	-2.438422	-0.033182
H	-6.000594	-4.544680	-0.506430
H	7.284223	-3.343938	-0.206839
H	-9.695019	0.048067	-0.245511
H	13.131494	0.004138	0.155014
H	-3.868651	-3.348518	-0.383679
H	7.280164	3.361153	0.310681
H	-3.864456	3.381902	0.097633
H	-8.464543	-4.531658	-0.549271
H	11.879158	-4.562746	-0.218833
H	11.873759	4.572998	0.484503
C	5.018213	0.010299	0.012260
C	3.602751	0.011169	-0.012474
C	2.389855	0.011207	-0.033680
C	-0.178218	0.008619	-0.078813
C	1.034661	0.010906	-0.057411
N	9.242171	0.007160	0.086452
N	-5.803786	0.020272	-0.177518
H	-8.441004	4.603339	0.101980

TRI(N)-CCCC-TRI(N) (tail-tail)

C	-5.512820	2.564205	0.053813
C	9.442374	-2.451529	-0.122999
C	-7.690482	-1.048010	-0.241526
C	11.619427	1.159062	0.195774
C	-3.461923	-1.119085	-0.187847
C	7.390832	1.229645	0.144077
C	11.582073	-1.258701	0.001829
C	-7.652710	1.370999	-0.063903
C	7.354159	-1.221608	-0.053129
C	-3.424824	1.333429	-0.007080
C	9.501520	2.406784	0.267100

C	-5.572805	-2.296619	-0.303563
C	-6.235146	3.782654	0.132904
C	10.164919	-3.669273	-0.210543
C	-8.389021	-2.256127	-0.339676
C	12.317760	2.366626	0.301850
C	-2.051764	-1.100570	-0.166801
C	5.980677	1.211002	0.123255
C	12.248989	-2.504625	-0.088696
C	-8.319405	2.617643	0.017970
C	5.958814	-1.188140	-0.069639
C	-2.029496	1.299803	0.010070
C	10.261517	3.596626	0.372650
C	-6.333002	-3.486996	-0.401296
C	-6.234986	1.347895	-0.045610
C	10.164336	-1.235767	-0.015717
C	-6.266129	-1.059591	-0.222509
C	10.195064	1.170478	0.177399
C	-4.161544	0.117688	-0.106760
C	8.090671	-0.006416	0.054449
C	-7.614029	3.801345	0.114694
C	-7.705211	-3.460082	-0.418328
C	11.633735	3.569880	0.388967
C	-1.343068	0.085075	-0.069648
C	5.272180	0.025894	0.018340
C	-4.129978	2.547927	0.072195
C	8.059523	-2.435409	-0.140747
C	-8.361009	0.184328	-0.160741
C	12.290171	-0.072560	0.106359
C	-4.181382	-2.307580	-0.285114
C	8.110093	2.417595	0.249188
H	-5.668736	4.703375	0.208402
H	9.598664	-4.589584	-0.291968
H	-9.471609	-2.226986	-0.352578
H	13.400359	2.337621	0.314125
H	-1.522732	-2.042790	-0.228645
H	5.451476	2.152683	0.191530
H	13.332460	-2.503390	-0.073700
H	-9.402870	2.616527	0.002515
H	5.411019	-2.117503	-0.151756
H	-1.481537	2.229588	0.086090
H	9.724307	4.535083	0.440537
H	-5.795960	-4.426004	-0.462586
H	13.373730	-0.090812	0.119659
H	-9.444558	0.202710	-0.174635

H	7.580248	3.359809	0.317548
H	-3.651703	-3.250341	-0.346905
H	7.509424	-3.365361	-0.222824
H	-3.579722	3.478297	0.148264
H	-8.259925	-4.388758	-0.493972
H	12.188290	4.498132	0.470722
C	11.543811	-3.687800	-0.193048
C	3.855319	0.041463	-0.000083
C	2.642382	0.049871	-0.016386
C	0.073788	0.069409	-0.050938
C	1.286724	0.060972	-0.034543
N	9.482697	-0.024828	0.071969
N	-5.553558	0.136258	-0.124891
H	-8.144056	4.744646	0.176252
H	12.074004	-4.630558	-0.261151

TRI(N)-Ph-TRI(N) (head-head)

C	-5.85603541	-1.14848608	-0.36054403
C	4.17689330	1.17342809	0.36608303
C	-7.94649060	2.35625117	0.73208905
C	6.26732943	-2.33132417	-0.72653505
C	-10.07982872	-1.13352108	-0.34828703
C	8.40068661	1.15844508	0.35381703
C	4.17374330	-1.15964808	-0.36812103
C	-5.85289744	1.18458608	0.37367503
C	6.27362045	2.34041517	0.72142705
C	-7.95275659	-2.31547917	-0.71590005
C	8.39757261	-1.15413108	-0.36200303
C	-10.07672775	1.17905109	0.36754503
C	-4.44155832	-1.11930708	-0.35266003
C	2.76241620	1.14425508	0.35820103
C	-8.66024963	3.50593925	1.08725608
C	6.98108349	-3.48101625	-1.08169908
C	-10.75014178	-2.32470117	-0.71621905
C	9.07100564	2.34962417	0.72174005
C	2.75934720	-1.12731808	-0.35814303
C	-4.43850032	1.15226208	0.36370003
C	6.99046649	3.48850625	1.07554407
C	-8.66959564	-3.46357125	-1.07002608
C	9.06468467	-2.34680817	-0.73090105
C	-10.74384478	2.37172317	0.73644506
C	-6.56992446	0.01884800	0.00708700
C	4.89077635	0.00608900	-0.00154200
C	-8.65533262	1.17497208	0.36631303
C	6.97617749	-1.15004708	-0.36076703

C	-8.65842762	-1.13261908	-0.34910703
C	6.97928450	1.15754809	0.35464103
C	-3.72623227	0.01568600	0.00498600
C	-10.04692570	3.50165925	1.08566908
C	8.36775860	-3.47674225	-1.08011708
C	-10.05625472	-3.45619325	-1.06643808
C	8.37712462	3.48112225	1.07195408
C	-6.54698548	-2.29604917	-0.71268305
C	4.86784935	2.32099117	0.71821205
C	-6.54077246	2.33368417	0.72682805
C	4.86161235	-2.30875117	-0.72126905
C	-10.76589475	0.02353400	0.01012300
C	9.08674665	0.00138500	-0.00458900
H	-3.91954428	-2.03396615	-0.60851104
H	2.24040616	2.05891915	0.61404505
H	-8.10483260	4.39417231	1.36213210
H	6.42566048	-4.36924831	-1.35656910
H	-11.83357684	-2.31700617	-0.71310405
H	10.15444171	2.34192417	0.71862205
H	2.23492116	-2.04081615	-0.61320905
H	-3.91407928	2.06576215	0.61877004
H	6.43743647	4.37798332	1.35121310
H	-8.11656160	-4.35304431	-1.34569910
H	10.14813773	-2.34154017	-0.72935405
H	-11.82729784	2.36645217	0.73489305
H	4.31419831	-3.20218723	-0.99823407
H	-5.99336241	3.22712123	1.00380007
H	10.17007371	0.00017000	-0.00537400
H	-11.84922086	0.02474500	0.01090400
H	4.32284231	3.21565123	0.99597407
H	-6.00197344	-3.19070423	-0.99045107
H	-10.58639074	4.40049832	1.36346110
H	8.90721867	-4.37558432	-1.35790810
H	-10.59812778	-4.35382531	-1.34344509
H	8.91900265	4.37875332	1.34895409
C	2.04708515	0.00926400	0.00056500
C	-2.25131216	0.01404800	0.00386100
C	-1.52945411	0.71002305	0.97363707
C	-1.53249511	-0.68353105	-0.96702207
C	-0.14665201	0.70848505	0.97257207
H	-2.05976615	1.23278609	1.76170413
C	-0.14969401	-0.68507005	-0.96808607
H	-2.06518015	-1.20511709	-1.75426913
C	0.57216404	0.01090700	0.00168800

H	0.38603303	1.23006809	1.75982113
H	0.38061803	-1.20783609	-1.75615112
N	6.28130548	0.00453100	-0.00255500
N	-7.96045357	0.02040000	0.00809900

TRI(N)-Ph-TRI(N) (head-tail)

C	11.957536	-1.237420	14.444414
C	3.016594	10.066681	15.495001
C	9.881531	2.441478	14.431387
C	4.836371	13.500627	13.850926
C	8.056933	-1.000433	16.064969
C	0.949444	13.758246	15.495087
C	4.935668	11.142184	14.405652
C	11.867767	1.158707	13.889108
C	1.047618	11.362333	16.045244
C	10.071491	-2.305914	15.522225
C	2.838778	14.814926	14.400102
C	7.972118	1.359597	15.510240
C	13.259733	-1.268083	13.890526
C	3.744095	8.856379	15.475261
C	9.157269	3.656765	14.441473
C	5.415306	14.673187	13.301155
C	7.473852	-2.152922	16.603413
C	-0.335053	13.817667	16.046040
C	5.608650	9.917302	14.420160
C	13.157976	1.067463	13.354998
C	-0.254065	11.483800	16.589407
C	9.430150	-3.437692	16.080176
C	3.468101	15.952293	13.838032
C	7.298171	2.603299	15.491827
C	11.253339	-0.002575	14.441729
C	3.622583	11.231443	14.949262
C	9.280611	1.278220	14.972893
C	3.533062	13.578344	14.404267
C	9.370659	-1.069247	15.516431
C	1.655648	12.520738	15.491328
C	13.837894	-0.140870	13.361321
C	4.737181	15.874269	13.298296
C	8.164345	-3.355287	16.605347
C	-0.921759	12.682991	16.586452
C	11.354293	-2.369398	14.985363
C	1.737107	10.153612	16.037893
C	11.157187	2.371520	13.896345
C	5.518999	12.297293	13.855751
C	7.378223	0.230398	16.049490

C	1.565874	14.893027	14.940983
H	13.789844	-2.213005	13.896422
H	3.280603	7.986957	15.926013
H	9.627336	4.524568	13.993682
H	6.411527	14.597722	12.881446
H	6.475013	-2.081726	17.016043
H	-0.856497	14.766905	16.039745
H	6.591971	9.869961	13.968733
H	13.609025	1.960034	12.939196
H	-0.710659	10.596136	17.010767
H	9.971021	-4.376518	16.080696
H	2.923517	16.889073	13.843088
H	6.310765	2.645439	15.936573
H	6.515112	12.240940	13.432512
H	11.616675	3.259699	13.478438
H	1.036064	15.838523	14.938232
H	6.378849	0.292051	16.464314
H	1.272968	9.270286	16.459657
H	11.889696	-3.311187	14.988584
H	5.200193	16.756929	12.872725
H	7.699310	-4.240073	17.026149
H	-1.917915	12.744058	17.010590
N	2.937736	12.444034	14.948037
N	9.967765	0.069547	14.977002
H	14.837305	-0.196003	12.943784
C	5.018712	8.771290	14.950546
C	7.874522	3.751274	14.964490
C	5.748018	7.488561	14.952178
C	7.125163	7.442816	15.170481
C	5.084855	6.280382	14.735867
C	7.809369	6.240922	15.175342
H	7.662069	8.362564	15.373043
C	5.768547	5.078336	14.740073
H	4.020814	6.288165	14.528716
C	7.145254	5.033398	14.960227
H	8.873634	6.235810	15.383237
H	5.230004	4.159861	14.534535

TRI(N)-Ph-TRI(N) (tail-tail)

C	10.523462	1.500488	14.447526
C	3.561385	9.027844	15.506239
C	8.733981	-1.960684	16.066473
C	5.350716	12.488621	13.886248
C	12.635977	-2.165538	14.449766
C	1.449179	12.694049	15.503974

C	5.474082	10.129164	14.433785
C	8.610629	0.398883	15.519407
C	1.572336	10.297129	16.047140
C	12.512786	0.231478	13.907008
C	3.333429	13.776714	14.425931
C	10.751459	-3.248571	15.526980
C	9.780631	2.701604	14.465800
C	4.304120	7.826668	15.487976
C	8.164767	-3.141378	16.607762
C	5.919851	13.669156	13.344538
C	13.925228	-2.207087	13.908081
C	0.160118	12.735816	16.046097
C	6.161251	8.912941	14.448185
C	7.923401	1.615072	15.505093
C	0.265635	10.400927	16.582395
C	13.819698	0.127903	13.372217
C	3.953933	14.923523	13.871677
C	10.130864	-4.395541	16.080795
C	9.928439	0.326935	14.984744
C	4.156385	10.201261	14.968697
C	10.042197	-2.020688	15.522240
C	4.042627	12.548793	14.430763
C	11.914687	-0.936704	14.452638
C	2.170375	11.465159	15.501120
C	8.857427	-4.334696	16.611299
C	5.227234	14.862500	13.340869
C	14.501676	-1.063337	13.376169
C	-0.416239	11.592229	16.578457
C	11.807957	1.431484	13.913577
C	2.277032	9.097044	16.040494
C	8.036950	-0.765282	16.061136
C	6.047708	11.293196	13.891728
C	12.028737	-3.309706	14.995495
C	2.056309	13.838049	14.957771
H	10.236782	3.578136	14.021288
H	3.848019	6.950194	15.932650
H	7.164690	-3.079148	17.020406
H	6.919827	13.606783	12.931672
H	14.458275	-3.149865	13.914844
H	-0.372857	13.678636	16.039307
H	7.148206	8.879014	14.003488
H	6.936362	1.648806	15.949619
H	-0.183165	9.506420	16.997610
H	14.268574	1.022533	12.957352

H	3.398240	15.853802	13.875915
H	10.686598	-5.325795	16.076484
H	7.047343	11.249962	13.475261
H	7.037228	-0.722187	16.477405
H	1.515346	14.777209	14.954299
H	12.569746	-4.248840	14.998891
H	1.821058	8.206520	16.456020
H	12.263978	2.322115	13.498337
H	8.401903	-5.224383	17.030335
H	5.682687	15.752065	12.921498
H	15.501689	-1.110751	12.959251
H	-1.416087	11.639822	16.995747
N	3.457020	11.405620	14.966671
N	10.627879	-0.877369	14.986720
C	5.582874	7.757007	14.971318
C	8.501773	2.771126	14.982220
C	6.327931	6.483422	14.973030
C	7.704784	6.454099	15.196178
C	5.680862	5.267418	14.752524
C	8.403747	5.260695	15.201074
H	8.229822	7.379928	15.402862
C	6.379834	4.074015	14.757425
H	4.617312	5.261939	14.542026
C	7.756680	4.044690	14.980577
H	9.467290	5.266198	15.411614
H	5.854771	3.148217	14.550668

TRI(B)-TRI(B) (head-head)

C	10.042567	2.635561	14.768309
C	5.094893	-10.773801	17.524615
C	7.946074	-1.104001	15.405708
C	7.195561	-7.041853	16.856654
C	12.221660	-1.090179	14.741447
C	3.310757	-7.704315	15.069999
C	7.073586	-9.326965	17.900875
C	7.934614	1.402968	15.200758
C	3.201955	-9.957582	16.099948
C	12.199416	1.381174	14.536903
C	5.311119	-6.227274	15.449852
C	10.089706	-2.349025	15.185584
C	9.314817	3.847753	14.781043
C	5.692063	-11.769035	18.332131
C	7.286956	-2.338317	15.613135
C	7.858609	-5.814199	16.623773
C	13.603735	-1.062213	14.522538

C	2.054465	-7.940975	14.500303
C	7.623184	-10.341910	18.692470
C	7.258348	2.628310	15.204657
C	1.934643	-10.149130	15.502927
C	13.596483	1.357449	14.320537
C	6.020976	-5.019459	15.254012
C	9.376713	-3.551755	15.400371
C	9.330580	1.434278	14.979690
C	5.804460	-9.568742	17.326874
C	9.351399	-1.134165	15.193201
C	5.919661	-7.226492	16.257491
C	11.540996	0.148884	14.743471
C	3.864151	-8.731920	15.867707
C	7.949557	3.826730	14.996491
C	6.932701	-11.541274	18.897585
C	14.270257	0.150462	14.316420
C	1.385938	-9.149967	14.721025
C	11.446471	2.586336	14.553307
C	3.822684	-10.942987	16.914342
C	7.279070	0.144040	15.407612
C	7.735095	-8.078246	17.654919
C	11.485965	-2.301939	14.960204
C	4.041622	-6.484785	14.880104
H	9.823849	4.793594	14.622668
H	5.180251	-12.709977	18.509574
H	6.219466	-2.352212	15.807544
H	8.821056	-5.625917	17.088277
H	14.172762	-1.987478	14.511070
H	1.586177	-7.183094	13.878721
H	8.595842	-10.205193	19.156451
H	6.185199	2.658247	15.370460
H	1.391874	-11.076891	15.656144
H	14.141051	2.282507	14.157670
H	5.600837	-4.245098	14.620469
H	9.902037	-4.500883	15.373348
H	8.709236	-7.922709	18.110698
H	6.205464	0.153516	15.575414
H	3.589437	-5.714390	14.261311
H	12.037864	-3.238009	14.952472
H	3.294594	-11.878717	17.079916
H	11.974074	3.523166	14.392467
H	15.341432	0.139565	14.149628
H	0.413949	-9.303595	14.266063
B	5.196115	-8.509281	16.483806

B	10.074352	0.149777	14.971813
H	7.397791	4.759912	15.005202
H	7.384610	-12.308608	19.515915
C	7.994767	-3.543752	15.610846
C	7.277017	-4.818403	15.833709

TRI(B)-TRI(B) (head-tail)

C	-0.666683	2.617090	0.514515
C	7.169428	0.780883	1.077810
C	-2.893609	-1.028867	-0.098345
C	9.303397	-1.566395	-1.842106
C	1.438694	-1.111744	-0.106699
C	11.501331	0.719285	1.112314
C	7.146501	-0.754208	-0.858999
C	-2.840904	1.442038	0.313154
C	9.348837	1.528702	2.086386
C	1.490970	1.341942	0.311959
C	11.461987	-0.837026	-0.863438
C	-0.771376	-2.294723	-0.304348
C	-1.368928	3.812032	0.711445
C	5.771883	0.782992	1.049197
C	-3.609074	-2.231650	-0.298883
C	10.015072	-2.330555	-2.795002
C	2.853290	-1.110806	-0.102059
C	12.192841	1.475908	2.090858
C	5.734996	-0.719525	-0.845848
C	-3.504456	2.673721	0.516681
C	10.094083	2.264620	3.040556
C	2.904415	1.281049	0.306603
C	12.121580	-1.608748	-1.827205
C	-1.523922	-3.459132	-0.498198
C	-1.428478	1.442866	0.316770
C	7.840096	0.001416	0.109445
C	-1.482491	-1.089163	-0.106016
C	10.049054	-0.832784	-0.892679
C	0.778545	0.129730	0.102764
C	10.079042	0.764285	1.133437
C	-2.767583	3.827339	0.710464
C	-2.921967	-3.415422	-0.493565
C	11.397523	-2.340617	-2.774098
C	3.570706	0.070615	0.100857
C	11.482932	2.222988	3.022404
C	0.765107	2.541010	0.507140
C	7.934574	1.520119	2.039583
C	-3.537819	0.220777	0.108269

C	7.883557	-1.513430	-1.808560
C	0.662348	-2.279151	-0.299716
C	12.150341	-0.066455	0.131393
H	-0.829633	4.741954	0.867573
H	5.216605	1.351516	1.789853
H	-4.694777	-2.229058	-0.300196
H	9.480492	-2.908801	-3.542478
H	3.393474	-2.043814	-0.227462
H	13.277818	1.472958	2.114559
H	5.171383	-1.271204	-1.592155
H	-4.589301	2.716864	0.521249
H	9.581694	2.862139	3.787675
H	3.482791	2.190597	0.434374
H	13.207110	-1.645057	-1.847244
H	-1.024735	-4.411183	-0.654411
H	7.330821	-2.086038	-2.549125
H	-4.624689	0.243676	0.109909
H	13.236806	-0.090365	0.128269
H	1.180068	-3.222011	-0.453959
H	7.392471	2.108544	2.774964
H	1.322533	3.460878	0.661716
H	-3.477060	-4.333984	-0.646369
H	11.936514	-2.928882	-3.508122
H	12.032693	2.794492	3.762739
C	5.051079	0.039558	0.096505
B	-0.711092	0.161197	0.104221
B	9.323168	-0.022077	0.116839
H	-3.283043	4.768287	0.865746

TRI(B)-TRI(B) (tail-tail)

C	10.02868470	2.50354318	14.74774108
C	5.20436337	6.29302744	14.34609005
C	7.94177960	-1.20244709	15.45650612
C	6.89539048	9.76484771	16.26437017
C	12.21621687	-1.23604809	14.74058608
C	3.02595822	10.03801473	14.33136701
C	7.04552648	7.34998253	15.61352512
C	7.92446259	1.29118409	15.21226912
C	3.17775423	7.63030153	13.68690596
C	12.19569887	1.24940309	14.50112205
C	4.90381335	11.09675579	15.62721811
C	10.06877971	-2.45429318	15.22104510
C	9.30243565	3.69684726	14.75167007
C	5.92631041	5.09710437	14.36028502
C	7.27047650	-2.42440717	15.68954313

C	7.43740954	10.91020080	16.89088524
C	13.61340599	-1.21667809	14.50263105
C	1.76308312	10.09816075	13.69055797
C	7.73587557	6.11865946	15.60100913
C	7.23522653	2.52330818	15.20648209
C	1.91216714	7.75068057	13.06204193
C	13.59242697	1.20670108	14.26888700
C	5.48172040	12.20325287	16.26080616
C	9.36001066	-3.63829026	15.45664910
C	9.31627469	1.30625309	14.98163605
C	5.78545041	7.41273253	14.98205109
C	9.33479669	-1.24714809	15.22635312
C	5.63251639	9.88653970	15.64323615
C	11.53242181	0.01185000	14.73427506
C	3.71129427	8.79106161	14.31446504
C	7.91385655	3.71461027	14.98054007
C	7.18479451	5.00261136	14.98329608
C	7.98071260	-3.61081726	15.68633515
C	6.73124748	12.09897485	16.88031323
C	14.26316104	-0.01059700	14.27468102
C	1.24010409	8.96715863	13.07727192
C	11.44310981	2.44749718	14.51147006
C	3.92269928	6.42768744	13.71454399
C	7.27056451	0.05008300	15.44593109
C	7.57008253	8.51445860	16.23907116
C	11.48235883	-2.42098917	14.97982510
C	3.62651826	11.14409678	14.97623008
H	9.80740669	4.63764633	14.55120106
H	5.52705840	4.21857331	13.86118001
H	6.20006846	-2.43479218	15.87102812
H	8.40541962	10.85990880	17.38001525
H	14.17749100	-2.14371615	14.49783406
H	1.20312809	11.02762180	13.67763700
H	8.69661162	6.03152043	16.09965714
H	6.16790443	2.54766318	15.40525609
H	1.46472511	6.89320848	12.56987491
H	14.14170804	2.12452015	14.08561800
H	4.96096936	13.15644493	16.27625618
H	9.87897071	-4.59256333	15.46312009
H	8.54080163	8.44477861	16.72336322
H	6.19845643	0.05971100	15.62532213
H	3.09073922	12.08953485	14.98067306
H	12.01863986	-3.36615124	14.98163108
H	3.50171725	5.55101140	13.22976196

H	11.96443687	3.38361324	14.33062506
H	7.45881852	-4.54386833	15.86599712
H	7.15348653	12.97302892	17.36299024
H	15.33284713	-0.01869700	14.09432503
H	0.27215302	9.03549562	12.59227192
B	10.06163572	0.02324000	14.98062506
B	5.04259836	8.69718965	14.97986410

TRI(B)-CC-TRI(B) (head-head)

C	10.060421	2.224970	13.973084
C	3.751298	-13.166588	17.936946
C	7.963028	-1.509480	14.628796
C	5.848574	-9.432020	17.281529
C	11.852869	-0.850921	16.414138
C	1.960627	-10.092219	15.492671
C	5.730020	-11.721262	18.315325
C	8.081020	0.780238	13.595992
C	1.855882	-12.347425	16.517486
C	11.957076	1.404740	15.390270
C	3.961832	-8.616584	15.868743
C	9.850982	-2.325959	16.039359
C	9.461647	3.223693	13.171258
C	4.349446	-14.164773	18.739907
C	7.297840	-2.733945	14.855860
C	6.513732	-8.207525	17.054547
C	13.111100	-0.614734	16.980458
C	0.702977	-10.328908	14.925272
C	6.280876	-12.739536	19.102268
C	7.529555	1.799035	12.810152
C	0.587633	-12.539027	15.922713
C	13.225924	1.595824	15.983931
C	4.665968	-7.408902	15.670986
C	9.146775	-3.533574	16.237273
C	9.351838	1.018971	14.168028
C	4.459847	-11.960554	17.742091
C	9.239414	-1.327299	15.230914
C	4.572832	-9.614758	16.678219
C	11.295898	0.178264	15.620604
C	2.517052	-11.120937	16.287193
C	8.219404	2.998774	12.608059
C	5.591062	-13.939311	19.304270
C	13.778013	0.594940	16.761337
C	0.036090	-11.538610	15.144318
C	11.333911	2.392313	14.580725
C	2.478444	-13.334481	17.328125

C	7.420571	-0.468422	13.837297
C	6.390452	-10.472583	18.074077
C	11.123822	-2.069955	16.604134
C	2.689600	-8.873115	15.302839
H	9.973547	4.165093	12.996760
H	3.837552	-15.106182	18.914375
H	6.323247	-2.922496	14.419749
H	7.487861	-8.018571	17.491521
H	13.581567	-1.374380	17.598266
H	0.232943	-9.569633	14.306679
H	7.254467	-12.604209	19.564747
H	6.555454	1.664149	12.348618
H	0.046485	-13.467814	16.075171
H	13.767102	2.524581	15.831398
H	4.244241	-6.616616	15.062695
H	9.568924	-4.326221	16.844801
H	7.365486	-10.317477	18.527824
H	6.445044	-0.623101	13.384464
H	2.236605	-8.100804	14.687228
H	11.577251	-2.842638	17.218958
H	1.951113	-14.270991	17.491826
H	11.861262	3.328801	14.416965
H	14.751276	0.747857	17.213697
H	-0.936716	-11.691922	14.691111
B	3.850040	-10.898592	16.902367
B	9.962272	-0.043528	15.006616
H	7.765889	3.768725	11.994255
H	6.044097	-14.708850	19.918944
C	5.924116	-7.214503	16.259065
C	7.888025	-3.727453	15.650312
C	6.612544	-5.991944	16.045608
C	7.199578	-4.949989	15.863839

TRI(B)-CC-TRI(B) (head-tail)

C	-3.267187	2.653994	0.377940
C	7.125988	1.240636	0.215810
C	-5.489306	-1.023202	-0.032447
C	9.260810	-2.485382	-0.207073
C	-1.157800	-1.103821	-0.045081
C	11.458373	1.226630	0.212090
C	7.103153	-1.220216	-0.062955
C	-5.439055	1.465877	0.246464
C	9.307204	2.485826	0.355768
C	-1.107705	1.374603	0.232936
C	11.418063	-1.271853	-0.070672

C	-3.367101	-2.296518	-0.177048
C	-3.971372	3.856497	0.513392
C	5.731235	1.225032	0.214602
C	-6.204401	-2.235297	-0.167563
C	9.973281	-3.698654	-0.344773
C	0.254307	-1.106131	-0.046242
C	12.151302	2.455318	0.350776
C	5.694139	-1.194268	-0.059511
C	-6.104692	2.705356	0.386024
C	10.053351	3.682386	0.490841
C	0.303264	1.320899	0.226091
C	12.078696	-2.498148	-0.209815
C	-4.119225	-3.470110	-0.307886
C	-4.026564	1.468979	0.245585
C	7.795279	0.002653	0.075289
C	-4.078070	-1.082278	-0.040357
C	10.005301	-1.292247	-0.072288
C	-1.817584	0.148984	0.095877
C	10.035936	1.270996	0.217853
C	-5.369885	3.869165	0.515939
C	-5.517126	-3.427315	-0.301777
C	11.355781	-3.687926	-0.344179
C	0.966169	0.093623	0.088008
C	11.442534	3.642004	0.485500
C	-1.835989	2.581069	0.368763
C	7.893198	2.445608	0.351948
C	-6.134521	0.234658	0.109085
C	7.841789	-2.428408	-0.200065
C	-1.934094	-2.280389	-0.176435
C	12.106915	-0.021835	0.070457
H	-3.433271	4.794217	0.618164
H	5.167042	2.146286	0.319223
H	-7.290090	-2.233172	-0.166277
H	9.438948	-4.637810	-0.450876
H	0.805099	-2.034188	-0.150625
H	13.236381	2.470785	0.351918
H	5.121792	-2.109946	-0.163033
H	-7.189591	2.746243	0.391512
H	9.541437	4.633147	0.598746
H	0.891265	2.226176	0.327393
H	13.164357	-2.534980	-0.214487
H	-3.619427	-4.428490	-0.415723
H	7.289142	-3.358459	-0.305213
H	-7.221421	0.256244	0.112488

H	13.193480	-0.039362	0.067914
H	-1.415523	-3.229151	-0.283215
H	7.351459	3.381418	0.458211
H	-1.279550	3.508470	0.472454
H	-6.071937	-4.352912	-0.404943
H	11.895426	-4.622154	-0.450178
H	11.992834	4.570993	0.590342
C	5.014921	0.015759	0.077824
C	2.386568	0.065408	0.084381
C	3.595098	0.039686	0.081105
B	-3.307366	0.178582	0.100290
B	9.278931	-0.005944	0.073677
H	-5.886867	4.816015	0.622540

TRI(B)-CC-TRI(B) (tail-tail)

C	-4.12600530	-1.22068809	0.00329400
C	4.12529229	1.22230709	-0.00032700
C	-6.28545645	2.51486018	0.00781200
C	6.28466247	-2.51329118	0.00127300
C	-8.45807159	-1.23524809	0.00532400
C	8.45735958	1.23676909	-0.00245700
C	4.11843530	-1.25497209	0.00141900
C	-4.11920230	1.25659009	0.00559600
C	6.29821047	2.49008118	-0.00229200
C	-6.29889543	-2.48851118	0.00312400
C	8.43327260	-1.27792709	-0.00065000
C	-8.43403959	1.27944809	0.00767900
C	-2.73187420	-1.19645209	0.00267100
C	2.73116120	1.19810209	0.00037000
C	-7.00625952	3.73118127	0.00930500
C	7.00543951	-3.72962827	0.00179400
C	-9.14320664	-2.47620418	0.00448200
C	9.14252066	2.47771018	-0.00368100
C	2.70964919	-1.23794209	0.00208200
C	-2.71041619	1.23959209	0.00491600
C	7.03670150	3.69917327	-0.00352000
C	-7.03736049	-3.69762026	0.00233400
C	9.10254667	-2.50724718	-0.00009500
C	-9.10334065	2.50875318	0.00916200
C	-4.80307735	0.02086700	0.00476700
C	4.80233735	-0.01926300	0.00021900
C	-7.02158651	1.30929710	0.00702800
C	7.02081952	-1.30774409	0.00005700
C	-7.03542449	-1.27051409	0.00461600
C	7.03471248	1.27206709	-0.00178400

C	-2.02469515	0.02577000	0.00347400
C	-8.38849262	3.71122727	0.00995200
C	8.38767260	-3.70970527	0.00111100
C	-8.42658258	-3.66592326	0.00302100
C	8.42592259	3.66744527	-0.00418100
C	-4.88563835	-2.43915318	0.00250700
C	4.88495135	2.44075518	-0.00156300
C	-4.86607135	2.46771318	0.00708700
C	4.86527835	-2.46611218	0.00192100
C	-9.11454264	0.01684000	0.00681600
C	9.11380264	-0.01533400	-0.00188300
H	-2.16178015	-2.11997715	0.00154400
H	2.16108615	2.12164115	-0.00000600
H	-6.47809946	4.67974934	0.00994700
H	6.47725849	-4.67818534	0.00272700
H	-10.22806275	-2.49932718	0.00497400
H	10.22737773	2.50080818	-0.00423200
H	2.14289615	-2.16285016	0.00300800
H	-2.14368316	2.16451315	0.00551400
H	6.51829248	4.65251433	-0.00394700
H	-6.51893146	-4.65094833	0.00119100
H	10.18837076	-2.53762218	-0.00060000
H	-10.18916473	2.53910418	0.00971400
H	4.31914731	-3.40579725	0.00285200
H	-4.31996131	3.40741024	0.00770900
H	10.20043976	-0.02612100	-0.00240600
H	-10.20117976	0.02760200	0.00734500
H	4.33686531	3.37882724	-0.00196200
H	-4.33753231	-3.37721324	0.00137000
H	-8.93496264	4.64745334	0.01109900
H	8.93412262	-4.64594433	0.00151600
H	-8.97120567	-4.60410933	0.00239500
H	8.97056663	4.60561933	-0.00512100
C	2.02395515	-0.02410400	0.00156400
C	0.60381204	-0.00903200	0.00223400
C	-0.60455204	0.01073000	0.00280200
B	-6.28701144	0.01971400	0.00546800
B	6.28627143	-0.01814500	-0.00050200

TRI(B)-CCCC-TRI(B) (head-head)

C	10.186981	2.449551	13.933893
C	2.635916	-15.149425	18.281546
C	8.090030	-1.284218	14.589054
C	4.733662	-11.416084	17.626487
C	11.980349	-0.625718	16.374279

C	0.842074	-12.072705	15.843339
C	4.615776	-13.705769	18.658047
C	8.207807	1.005128	13.556729
C	0.738707	-14.328400	16.866365
C	12.083604	1.629645	15.350507
C	2.843936	-10.598281	16.217034
C	9.979183	-2.100916	15.999916
C	9.587986	3.448238	13.132223
C	3.235057	-16.148578	19.082525
C	7.424307	-2.508344	14.815576
C	5.399618	-10.192070	17.400050
C	13.238758	-0.389584	16.940609
C	-0.416769	-12.308215	15.277712
C	5.167876	-14.724957	19.443210
C	7.655838	2.023870	12.770895
C	-0.530613	-14.519007	16.273608
C	13.352489	1.820888	15.943990
C	3.547375	-9.390457	16.018569
C	9.275996	-3.308879	16.198425
C	9.478444	1.243235	14.128866
C	3.344733	-13.943275	18.086562
C	9.366592	-1.102670	15.191415
C	3.456697	-11.597021	17.024795
C	11.422547	0.402825	15.580901
C	1.400032	-13.101720	16.635991
C	8.345651	3.223642	12.568916
C	4.477795	-15.924577	19.645181
C	13.905289	0.820272	16.721369
C	-1.083565	-13.517928	15.496934
C	11.460360	2.617117	14.541245
C	1.362117	-15.316362	17.674902
C	7.546994	-0.243496	13.797668
C	5.276838	-12.457265	18.417174
C	11.251985	-1.845186	16.564680
C	1.570714	-10.853400	15.652939
H	10.099701	4.389731	12.957702
H	2.723137	-17.089963	19.257029
H	6.449748	-2.697940	14.380129
H	6.374481	-10.002928	17.835014
H	13.709493	-1.149050	17.558252
H	-0.887640	-11.548368	14.660642
H	6.142224	-14.590573	19.904126
H	6.681799	1.889017	12.309463
H	-1.071870	-15.447716	16.426131

H	13.893530	2.749724	15.791476
H	3.125792	-8.596969	15.411957
H	9.697462	-4.102006	16.805588
H	6.252579	-12.303311	18.869465
H	6.571564	-0.397915	13.344866
H	1.116716	-10.080684	15.038819
H	11.705855	-2.617531	17.179361
H	0.834737	-16.252784	17.838649
H	11.987530	3.553658	14.377492
H	14.878591	0.973721	17.173408
H	-2.057201	-13.670889	15.045451
B	2.733857	-12.880662	17.249021
B	10.089168	0.181112	14.967138
H	7.892326	3.993823	11.955302
H	4.931228	-16.695113	20.258268
C	4.807010	-9.199027	16.605804
C	8.016756	-3.500908	15.610540
C	5.494092	-7.979319	16.392771
C	7.329907	-4.720728	15.823680
C	6.083557	-6.933514	16.210265
C	6.740628	-5.766618	16.006298

TRI(B)-CCCC-TRI(B) (head-tail)

C	-5.836726	2.672587	0.366610
C	7.122156	1.234046	0.212074
C	-8.050051	-1.009771	-0.045097
C	9.263294	-2.489446	-0.209089
C	-3.719075	-1.081407	-0.053910
C	11.454418	1.226200	0.212968
C	7.103685	-1.228228	-0.067209
C	-8.005540	1.479364	0.233381
C	9.301561	2.482369	0.354514
C	-3.674695	1.398249	0.223803
C	11.417931	-1.272101	-0.070132
C	-5.925228	-2.278695	-0.187594
C	-6.543906	3.873577	0.501231
C	5.728100	1.217354	0.209454
C	-8.762479	-2.223327	-0.180595
C	9.978173	-3.701429	-0.346168
C	-2.307336	-1.081738	-0.053910
C	12.145730	2.455886	0.352536
C	5.695114	-1.205515	-0.065434
C	-8.674166	2.717253	0.372117
C	10.045910	3.679778	0.490495
C	-2.263882	1.348771	0.218373

C	12.081054	-2.497149	-0.208706
C	-6.674862	-3.453999	-0.318871
C	-6.592989	1.485657	0.233761
C	7.793075	-0.003604	0.072051
C	-6.638610	-1.065809	-0.051755
C	10.005362	-1.294839	-0.073289
C	-4.381146	0.170500	0.086270
C	10.031937	1.268386	0.217211
C	-7.942315	3.882950	0.502510
C	-8.072721	-3.414083	-0.313989
C	11.360617	-3.688282	-0.344045
C	-1.599478	0.121330	0.080955
C	11.435286	3.641390	0.486627
C	-4.405612	2.603292	0.358776
C	7.887743	2.440402	0.349209
C	-8.698065	0.246634	0.095618
C	7.844534	-2.435462	-0.203712
C	-4.492530	-2.259883	-0.185755
C	12.104744	-0.021139	0.071890
H	-6.008047	4.812526	0.606324
H	5.161435	2.137043	0.313474
H	-9.848162	-2.223402	-0.180249
H	9.445607	-4.641479	-0.452992
H	-1.753377	-2.007870	-0.157537
H	13.230755	2.472680	0.354803
H	5.123462	-2.121443	-0.169673
H	-9.759158	2.755556	0.376615
H	9.532557	4.629773	0.597962
H	-1.677021	2.254635	0.319967
H	13.166774	-2.531880	-0.212166
H	-6.172907	-4.411294	-0.426073
H	7.293439	-3.366269	-0.309582
H	-9.785008	0.265812	0.098053
H	13.191324	-0.037140	0.070494
H	-3.971554	-3.207331	-0.291866
H	7.344373	3.375233	0.454983
H	-3.851104	3.531746	0.462778
H	-8.625576	-4.340788	-0.417450
H	11.902067	-4.621478	-0.449571
H	11.984056	4.571167	0.592160
C	5.015473	0.004979	0.071490
C	3.598996	0.026109	0.072985
C	2.385784	0.049731	0.074854
C	-0.182831	0.096152	0.078651

C	1.030638	0.074206	0.076811
B	-5.870974	0.196805	0.089340
B	9.277032	-0.009779	0.072058
H	-8.461684	4.828536	0.608473

TRI(B)-CCCC-TRI(B) (tail-tail)

C	-3.69892027	-1.20435609	-0.11904001
C	7.10516450	1.22854209	0.20356801
C	-5.87262040	2.50278218	0.28031002
C	9.27881069	-2.47805218	-0.20109401
C	-8.03085256	-1.23207709	-0.14242501
C	11.43709783	1.25616609	0.22698902
C	7.10815451	-1.23475609	-0.07230500
C	-3.70194627	1.25933709	0.15328701
C	9.27367465	2.49513718	0.35551603
C	-5.86741145	-2.47119918	-0.26917602
C	11.42235384	-1.24275009	-0.05265300
C	-8.01614657	1.26723609	0.13364701
C	-2.30545216	-1.17654708	-0.10936501
C	5.71169543	1.20076609	0.19386201
C	-6.59836250	3.70908527	0.41012603
C	10.00453373	-3.68417926	-0.33262302
C	-8.71163163	-2.46772618	-0.28206402
C	12.11789388	2.49160518	0.36839103
C	5.69965840	-1.22354109	-0.07779401
C	-2.29344916	1.24815209	0.15877801
C	10.00765371	3.69865027	0.49363004
C	-6.60137347	-3.67491926	-0.40557103
C	12.09655688	-2.46236718	-0.18591301
C	-8.69036765	2.48703218	0.26517402
C	-4.38034932	0.02833000	0.01398800
C	7.78657556	-0.00396000	0.06876500
C	-6.60397850	1.30164609	0.14411901
C	10.01018572	-1.27712209	-0.06318500
C	-6.60815546	-1.26264009	-0.13912801
C	10.01440072	1.28675409	0.22373502
C	-1.60427212	0.04257700	0.02877300
C	-7.98063560	3.68446427	0.40081503
C	11.38680683	-3.65959426	-0.32326502
C	-7.99105156	-3.64776526	-0.40904103
C	11.39733180	3.67147226	0.49705704
C	-4.45421932	-2.41773117	-0.25668402
C	7.86048056	2.44170818	0.34295302
C	-4.45351332	2.46083118	0.28247902
C	7.85970357	-2.43607518	-0.20321401

C	-8.69189962	0.01028700	-0.00834000
C	12.09812589	0.01398500	0.09113001
H	-1.73069612	-2.09179515	-0.20781701
H	5.13695337	2.11587915	0.29364802
H	-6.07427641	4.65386733	0.51699404
H	9.48043368	-4.62880034	-0.44084203
H	-9.79643473	-2.49318918	-0.28992302
H	13.20269693	2.51704118	0.37628503
H	5.13589237	-2.14414215	-0.18362401
H	-1.72969813	2.16891416	0.26326802
H	9.48614270	4.64462333	0.59705504
H	-6.07984844	-4.62103133	-0.50764004
H	13.18250993	-2.48797618	-0.18359801
H	-9.77632070	2.51262018	0.26283202
H	7.31695155	-3.37156624	-0.31057602
H	-3.91077528	3.39648325	0.38849603
H	13.18478195	0.00681800	0.09544201
H	-9.77855568	0.01743200	-0.01265900
H	7.30887451	3.37214224	0.44456203
H	-3.90259928	-3.34830324	-0.35695203
H	-8.53032360	4.61341933	0.50079204
H	11.93648187	-4.58841433	-0.42456103
H	-8.53172163	-4.58222533	-0.51475204
H	11.93801585	4.60577233	0.60410204
C	5.01049736	-0.01814500	0.05395400
C	3.59396426	-0.00832500	0.04823800
C	2.38077817	0.00551500	0.04390600
C	-0.18773801	0.03278900	0.03449500
C	1.02544807	0.01895500	0.03883400
B	-5.86450240	0.02222400	0.00629000
B	9.27072867	0.00211200	0.07647301

TRI(B)-Ph-TRI(B) (head-head)

C	-5.84127740	-1.16980508	-0.36444903
C	4.16212930	1.19473909	0.36999103
C	-8.02147560	2.40008017	0.74225405
C	6.34232644	-2.37514717	-0.73671305
C	-10.17533273	-1.17434109	-0.35943003
C	8.49618564	1.19927309	0.36497103
C	4.15893130	-1.18092708	-0.37202403
C	-5.83807942	1.20586108	0.37756603
C	6.34871948	2.38407517	0.73148705
C	-8.02786658	-2.35914217	-0.72594605
C	8.49296961	-1.19515409	-0.37331803
C	-10.17211771	1.22008709	0.37885803

C	-4.42724032	-1.14142808	-0.35605503
C	2.74809320	1.16636308	0.36159703
C	-8.74726765	3.54394125	1.09438208
C	7.06811748	-3.51900825	-1.08884108
C	-10.86648881	-2.35325317	-0.72215905
C	9.18734166	2.37818617	0.72769905
C	2.74497420	-1.14940308	-0.36152703
C	-4.42412332	1.17433809	0.36706903
C	7.07757949	3.52630325	1.08257808
C	-8.75672664	-3.50137025	-1.07703708
C	9.18095767	-2.37561617	-0.73702705
C	-10.86010677	2.40054817	0.74256705
C	-6.52693246	0.01879400	0.00706900
C	4.84778435	0.00614000	-0.00152700
C	-8.76006564	1.24803409	0.38741803
C	7.08091654	-1.22310109	-0.38187703
C	-8.76336061	-1.20545109	-0.37002503
C	7.08421252	1.23038409	0.37556603
C	-3.73130627	0.01568300	0.00498500
C	-10.14615273	3.53189825	1.09084908
C	8.46700262	-3.50696525	-1.08530908
C	-10.15557373	-3.48619525	-1.07148208
C	8.47642759	3.51112725	1.07702308
C	-6.59445850	-2.31577017	-0.71461305
C	4.91531136	2.34070417	0.72015405
C	-6.58818648	2.35350617	0.72882705
C	4.90903736	-2.32857217	-0.72328605
C	-10.84446979	0.02362400	0.01019900
C	9.16532164	0.00130800	-0.00465800
H	-3.86980328	-2.03862615	-0.60483004
H	2.19065616	2.06356115	0.61037204
H	-8.22658359	4.45513932	1.37479510
H	6.54743247	-4.43020632	-1.36925410
H	-11.95204487	-2.37185617	-0.72714905
H	10.27289772	2.39678817	0.73268905
H	2.18517415	-2.04536115	-0.60946304
H	-3.86432328	2.07029615	0.61500405
H	6.55934346	4.43865932	1.36375810
H	-8.23848960	-4.41372632	-1.35821710
H	10.26646076	-2.39665217	-0.74358105
H	-11.94560986	2.42158418	0.74912105
H	4.37018331	-3.23051423	-1.00076207
H	-6.04933243	3.25544823	1.00630307
H	10.25245476	0.00008900	-0.00544000

H	-11.93160285	0.02484200	0.01098100
H	4.37888031	3.24384423	0.99842807
H	-6.05802744	-3.21891023	-0.99288707
H	-10.68013978	4.43360932	1.36851010
H	9.00098967	-4.40867732	-1.36297010
H	-10.69198075	-4.38670432	-1.34838210
H	9.01283467	4.41163632	1.35392210
C	2.05215815	0.00925200	0.00055600
C	-2.25419816	0.01404100	0.00385900
C	-1.52963511	0.68441505	0.99004007
C	-1.53264311	-0.65794205	-0.98343507
C	-0.14650501	0.68287705	0.98897707
H	-2.06015115	1.18652909	1.79148313
C	-0.14951301	-0.65948005	-0.98449807
H	-2.06550515	-1.15887608	-1.78405913
C	0.57505004	0.01089400	0.00168300
H	0.38635703	1.18381108	1.78960113
H	0.38100303	-1.16159408	-1.78594013
B	-8.01694060	0.02045600	0.00816100
B	6.33779245	0.00447700	-0.00262000

TRI(B)-Ph-TRI(B) (head-tail)

C	12.034995	-1.292228	14.419289
C	3.011979	10.065790	15.504615
C	9.881796	2.468461	14.417779
C	4.864313	13.596698	13.817386
C	8.062424	-1.081883	16.093387
C	0.881163	13.819645	15.505520
C	4.938152	11.150400	14.399198
C	11.930807	1.121361	13.859975
C	0.971035	11.406231	16.067669
C	10.089101	-2.400518	15.543145
C	2.831117	14.935554	14.373815
C	7.945124	1.365371	15.526226
C	13.335317	-1.345276	13.866771
C	3.720113	8.844843	15.498594
C	9.156445	3.682632	14.428921
C	5.446369	14.766843	13.270004
C	7.494630	-2.242474	16.632123
C	-0.404347	13.866620	16.057562
C	5.597853	9.918893	14.415682
C	13.220103	1.018460	13.324453
C	-0.329307	11.504312	16.613412
C	9.472725	-3.545233	16.098716
C	3.464226	16.072541	13.812756

C	7.268339	2.607150	15.509104
C	11.359161	-0.052133	14.402700
C	3.639317	11.202042	14.951357
C	9.254040	1.320405	14.973418
C	3.554558	13.710238	14.362793
C	9.364690	-1.188123	15.553656
C	1.549052	12.574434	15.523009
C	13.902288	-0.202680	13.333625
C	4.742464	15.964851	13.279456
C	8.199483	-3.450710	16.628927
C	-0.990416	12.718615	16.600651
C	11.394634	-2.428849	14.982635
C	1.704308	10.188811	16.049297
C	11.184778	2.345755	13.878963
C	5.516318	12.341516	13.845739
C	7.387338	0.182903	16.068295
C	1.533043	14.963013	14.935193
H	13.889411	-2.278928	13.859550
H	3.274926	7.958395	15.940066
H	9.591310	4.573169	13.986803
H	6.443048	14.729697	12.842252
H	6.496300	-2.213350	17.059320
H	-0.959420	14.800327	16.068636
H	6.588759	9.831684	13.979247
H	13.703463	1.891430	12.895174
H	-0.810237	10.630443	17.042251
H	9.995129	-4.496931	16.110554
H	2.951842	17.029081	13.798479
H	6.279453	2.686920	15.948872
H	6.515734	12.271700	13.424832
H	11.651484	3.230249	13.453920
H	0.993492	15.906331	14.936274
H	6.387547	0.232229	16.491157
H	1.239053	9.302865	16.474033
H	11.933687	-3.372902	14.984946
H	5.207990	16.847986	12.854891
H	7.733023	-4.332451	17.053445
H	-1.987382	12.786026	17.021200
B	2.914220	12.496041	14.945462
B	9.992724	0.026480	14.976567
H	14.899652	-0.250723	12.911469
C	4.999682	8.766866	14.960460
C	7.868516	3.747164	14.967546
C	5.733097	7.485212	14.958994

C	7.104254	7.437876	15.211578
C	5.078537	6.279321	14.707075
C	7.790063	6.236668	15.214751
H	7.636913	8.353320	15.445006
C	5.764655	5.078408	14.710667
H	4.020483	6.286122	14.468966
C	7.135793	5.030020	14.964678
H	8.848613	6.229480	15.449357
H	5.234121	4.162169	14.476844

TRI(B)-Ph-TRI(B) (tail-tail)

C	-3.794060	-1.202438	-0.129483
C	6.197439	1.241119	0.209314
C	-5.963917	2.499392	0.292786
C	8.367307	-2.460598	-0.213847
C	-8.126277	-1.230680	-0.149472
C	10.529656	1.269379	0.229275
C	6.197804	-1.213589	-0.083178
C	-3.794417	1.252344	0.162432
C	8.365561	2.508142	0.362730
C	-5.962188	-2.469484	-0.282614
C	10.513853	-1.229241	-0.060641
C	-8.110465	1.268009	0.139847
C	-2.397614	-1.169810	-0.117819
C	4.800993	1.208489	0.197646
C	-6.687657	3.706104	0.428075
C	9.091051	-3.667274	-0.349438
C	-8.806613	-2.465469	-0.295189
C	11.209987	2.504136	0.375278
C	4.786513	-1.194322	-0.088665
C	-2.383126	1.233075	0.167923
C	9.099641	3.711461	0.505021
C	-6.696272	-3.672833	-0.424625
C	11.185909	-2.449516	-0.198025
C	-8.782517	2.488319	0.276935
C	-4.476285	0.026186	0.012874
C	6.879671	0.012534	0.066664
C	-6.697984	1.300850	0.150903
C	9.101372	-1.262087	-0.071689
C	-6.703654	-1.262148	-0.146583
C	9.107033	1.300842	0.226407
C	-1.688128	0.037134	0.030021
C	-8.070137	3.683581	0.418336
C	10.473532	-3.644747	-0.339704
C	-8.085703	-3.645269	-0.427888

C	10.489072	3.683902	0.508265
C	-4.548519	-2.415188	-0.270502
C	6.951893	2.453841	0.350616
C	-4.543503	2.453845	0.295245
C	6.946893	-2.415057	-0.216272
C	-8.786960	0.011911	-0.008781
C	11.190343	0.026825	0.088280
H	-1.835532	-2.095646	-0.200798
H	4.238923	2.134314	0.280830
H	-6.162374	4.649778	0.539364
H	8.565772	-4.610924	-0.460944
H	-9.891464	-2.491418	-0.303161
H	12.294838	2.530090	0.383245
H	4.232634	-2.124036	-0.176537
H	-1.829227	2.162798	0.255596
H	8.578390	4.657299	0.611726
H	-6.175024	-4.618699	-0.531099
H	12.271825	-2.477469	-0.195331
H	-9.868434	2.516275	0.274223
H	6.403422	-3.349969	-0.327213
H	-4.000029	3.388782	0.405962
H	12.277018	0.019209	0.092569
H	-9.873635	0.019531	-0.013083
H	6.400802	3.384516	0.455405
H	-3.997433	-3.345890	-0.375064
H	-8.618162	4.613153	0.522435
H	11.021560	-4.574291	-0.444027
H	-8.626691	-4.579113	-0.537964
H	11.030057	4.617722	0.618557
C	4.091503	0.001581	0.049521
B	-5.959836	0.021391	0.005863
B	8.363221	0.017334	0.073665
C	-0.211494	0.026450	0.036481
C	0.512377	0.856366	0.892830
C	0.507933	-0.811735	-0.815705
C	1.895435	0.850445	0.895252
H	-0.017325	1.491927	1.593835
C	1.890989	-0.817654	-0.813284
H	-0.023894	-1.443494	-1.518582
C	2.614865	0.012261	0.043067
H	2.427249	1.482198	1.598142
H	2.420680	-1.453204	-1.514305

TAM-TAM

C	10.01028970	2.53306418	14.93332005

C	5.17181437	6.23230245	14.52085004
C	7.90925959	-1.07153007	15.08899109
C	7.03751850	9.70109170	15.90601314
C	12.08116189	-1.09428808	14.92301009
C	3.10281722	9.86070770	14.51848102
C	7.11985652	7.35795654	15.43853012
C	7.90795157	1.31838110	15.02627707
C	3.18337523	7.47405353	14.05205403
C	12.08006284	1.33827310	14.86919308
C	5.01066736	10.96311678	15.43792109
C	10.01603371	-2.28747417	15.03327010
C	9.32931468	3.72276627	14.93436708
C	5.85277742	5.04258836	14.52003802
C	7.21549552	-2.26408216	15.14724609
C	7.65141856	10.84754479	16.37037819
C	13.46197695	-1.10169308	14.86550705
C	1.80129113	9.91389371	14.05671402
C	7.81024857	6.17370543	15.44012109
C	7.21738653	2.50254018	15.02588310
C	1.88367414	7.51480854	13.58728797
C	13.45969397	1.34366810	14.81044806
C	5.61470740	12.11738085	15.89924713
C	9.33277465	-3.48745025	15.09024810
C	9.30806768	1.32174910	14.97958210
C	5.79695044	7.39911452	14.97943108
C	9.30328069	-1.08944708	15.03285110
C	5.72267242	9.76466472	15.44350312
C	11.39930780	0.12159601	14.92467906
C	3.78512627	8.64509564	14.51392602
C	7.91664156	3.73004027	14.98023310
C	7.18820653	4.99077736	14.98019008
C	7.94110156	-3.45517325	15.14654409
C	6.92763050	12.03968286	16.35877719
C	14.12912103	0.12011401	14.80993306
C	1.21270809	8.73746464	13.59785998
C	9.99939473	0.12516401	14.97904208
C	5.10574837	8.59576462	14.97896810
H	9.88636173	4.64706533	14.85894006
H	5.36743240	4.15962630	14.12616203
H	6.13384944	-2.26554116	15.19149309
H	8.67100463	10.81316880	16.73257022
H	14.00402903	-2.03874415	14.86393809
H	1.25973609	10.85123178	14.05499700
H	8.81748164	6.15322744	15.83429814

H	6.13841942	2.48159018	15.10149406
H	1.40408410	6.61421549	13.22556696
H	14.00122100	2.28002016	14.76672706
H	5.07335036	13.05483395	15.90013514
H	9.87381970	-4.42508432	15.09084607
H	7.40050255	-4.39297431	15.19121210
H	7.40595456	12.94154795	16.72148120
H	15.21156309	0.12005601	14.76485108
H	0.19259301	8.77352262	13.23484693
O	3.87591228	6.28894246	14.05913702
O	3.71657727	11.00085077	14.97788408
O	7.71804854	8.50903962	15.89991617
O	11.38578880	2.52239518	14.87346508
O	7.23020252	0.12137301	15.08583111
O	11.38882385	-2.27978416	14.97776607

TAM-CC-TAM

C	10.72278257	1.27495730	15.18961901
C	4.56109484	7.13607924	14.76829602
C	8.66258346	-2.30871715	14.55954819
C	6.62183966	10.71946887	15.39820174
C	12.78901547	-2.35410021	15.18656042
C	2.49538124	10.76543188	14.77140674
C	6.64220822	8.33860972	15.19189696
C	8.64187125	0.07214123	14.76583503
C	2.50870873	8.34265052	14.56081261
C	12.77533528	0.06867861	15.39716063
C	4.52963552	11.94096072	15.18568331
C	10.75495274	-3.52991233	14.77214743
C	10.04623084	2.46547814	15.19136313
C	5.23748279	5.94546594	14.76648724
C	7.98440260	-3.49345772	14.35175299
C	7.30020404	11.90411712	15.60592318
C	14.15543892	-2.37114044	15.39421513
C	1.12894985	10.78266438	14.56382061
C	7.33597341	7.15799268	15.19364123
C	7.94793366	1.25265772	14.76408933
C	1.14370204	8.34648272	14.35234699
C	14.14032969	0.06503699	15.60571029
C	5.19712058	13.13352970	15.39241809
C	10.08764975	-4.72257325	14.56535512
C	10.03002976	0.07199458	14.97780703
C	5.25403353	8.33894792	14.98003402
C	10.04169928	-2.33223739	14.76825215
C	5.24271817	10.74318410	15.18955763

C	12.10734966	-1.13816703	15.18897474
C	3.17687334	9.54940140	14.76897332
C	8.71088735	-4.68376584	14.35823345
C	6.57389070	13.09452987	15.59945335
C	14.80901875	-1.15867420	15.60064368
C	0.47518555	9.57028808	14.35744715
C	10.72119726	-1.12386004	14.97822127
C	4.56303521	9.53490025	14.97964963
H	10.56954554	3.39842294	15.35357156
H	4.71404194	5.01260199	14.60422134
H	6.91428635	-3.48901120	14.18850133
H	8.37033056	11.89952133	15.76910387
H	14.69621658	-3.30889034	15.39470753
H	0.58830457	11.72049061	14.56335271
H	8.40560991	7.14563301	15.35577617
H	6.87829070	1.26485860	14.60198450
H	0.61329182	7.41697568	14.18957334
H	14.67059810	0.99461709	15.76852892
H	4.65472886	14.07034589	15.39176498
H	10.63017650	-5.65931123	14.56601574
H	8.18287471	-5.61568628	14.19556346
H	7.10204764	14.02637862	15.76206565
H	15.88000466	-1.16629074	15.76393053
H	-0.59580943	9.57805423	14.19422681
O	3.20081263	7.15869628	14.56264647
O	3.17224822	11.94210369	14.97824090
O	7.30184841	9.52850096	15.39746089
O	12.08306037	1.25253279	15.39532010
O	7.98240718	-1.11784468	14.56025316
O	12.11232747	-3.53086554	14.97967296
C	6.63720686	5.94518865	14.97992967
C	8.64651616	2.46556205	14.97784386
C	7.33765106	4.73241479	14.97931371
C	7.94588823	3.67824841	14.97842490

TAM-CCCC-TAM

C	11.18511182	0.47480203	15.18656211
C	3.74139827	8.55546259	14.77097308
C	9.12538665	-3.10883623	14.55436802
C	5.80102643	12.13914686	15.40324712
C	13.25154496	-3.15365623	15.18225112
C	1.67487812	12.18387287	14.77529206
C	5.82253843	9.75902370	15.19589309
C	9.10394364	-0.72870505	14.76163606
C	1.68915612	9.76101171	14.56354306

C	13.23733195	-0.73079105	15.39396012
C	3.70814127	13.35984394	15.19066408
C	11.21823880	-4.32958631	14.76696805
C	10.50851775	1.66556412	15.18885811
C	4.41801532	7.36471752	14.76870709
C	8.44748059	-4.29350831	14.34595506
C	6.47889148	13.32382798	15.61173910
C	14.61788803	-3.17033823	15.39020211
C	0.30854202	12.20052687	14.56728804
C	6.51732149	8.57871961	15.19727409
C	8.40919858	0.45161903	14.76018606
C	0.32431602	9.76423472	14.35464801
C	14.60218305	-0.73404105	15.60278611
C	4.37513332	14.55251003	15.39815909
C	10.55120876	-5.52224241	14.55953507
C	10.49200573	-0.72776805	14.97397309
C	4.43447632	9.75805070	14.98357110
C	10.50450474	-3.13205823	14.76339505
C	4.42190832	12.16233488	15.19421910
C	12.56963488	-1.93772414	15.18506110
C	2.35681917	10.96795881	14.77246806
C	9.17451768	-5.48344239	14.35216405
C	5.75182040	14.51374203	15.60556212
C	15.27088508	-1.95770514	15.59726811
C	-0.34441702	10.98788180	14.36016606
C	11.18366378	-1.92376814	14.97401505
C	3.74278627	10.95403176	14.98355109
H	11.03053079	2.59910319	15.35158313
H	3.89603828	6.43116546	14.60593703
H	7.37742555	-4.28934131	14.18248204
H	7.54894254	13.31968394	15.77524312
H	15.15905109	-4.10784329	15.39043712
H	-0.23264302	13.13801792	14.56705404
H	7.58689153	8.56558660	15.35953211
H	7.33962552	0.46478003	14.59795503
H	-0.20570201	8.83464365	14.19126104
H	15.13223111	0.19554101	15.76612413
H	3.83251428	15.48917212	15.39781613
H	11.09380181	-6.45891847	14.55990105
H	8.64678662	-6.41543148	14.18901500
H	6.27951844	15.44573810	15.76877811
H	16.34183415	-1.96513514	15.76077012
H	-1.41535610	10.99529176	14.19660504
O	2.38185617	8.57757463	14.56505407

O	2.35115117	13.36036698	14.98284909
O	6.48129447	10.94848979	15.40210310
O	12.54465793	0.45266103	15.39246313
O	8.44515459	-1.91815914	14.55546305
O	12.57523490	-4.33013831	14.97475109
C	5.81754041	7.36682555	14.98263207
C	9.10900364	1.66349412	14.97487507
C	6.51766345	6.15535642	14.98139305
C	7.12746450	5.09766636	14.97967408
C	7.79913558	3.93268828	14.97777406
C	8.40891161	2.87498020	14.97608609

TAM-Ph-TAM

C	11.31296279	0.26308102	15.11456411
C	4.25732230	7.65310155	14.84478208
C	9.23090168	-3.32909224	14.69468106
C	6.33875343	11.24571781	15.26393412
C	13.38548094	-3.36322124	15.11920711
C	2.18440116	11.27916080	14.83706806
C	6.34556347	8.86026763	15.11722010
C	9.22441264	-0.94371507	14.84266008
C	2.19379716	8.84952162	14.69582705
C	13.37642799	-0.93366407	15.26176709
C	4.23826030	12.46651792	15.11956610
C	11.33133784	-4.55019733	14.83720405
C	10.62936279	1.45694911	15.11499406
C	4.94115436	6.45936748	14.84429508
C	8.54288563	-4.51747333	14.55029706
C	7.02666449	12.43431891	15.40698809
C	14.75937706	-3.37284424	15.26069212
C	0.81068706	11.28862080	14.69377003
C	7.03872751	7.67190756	15.11784707
C	8.53149562	0.24479402	14.84193109
C	0.82064806	8.84660961	14.55123704
C	14.74976804	-0.93091407	15.40455811
C	4.91574535	13.66177999	15.26122812
C	10.65375278	-5.74523543	14.69419708
C	10.61467976	-0.93917307	14.97889105
C	4.95530936	8.85547063	14.98090605
C	10.61928974	-3.35032824	14.83702107
C	4.95036336	11.26669281	15.12150411
C	12.70140293	-2.14719616	15.12033510
C	2.86853521	10.06317372	14.83767407
C	9.26734368	-5.70921243	14.55264703
C	6.30212246	13.62600697	15.40312213

C	15.42057609	-2.15365116	15.40153709
C	0.14975101	10.06930572	14.55278203
C	11.31032183	-2.14314915	14.97919506
C	4.25946431	10.05931871	14.98032205
H	11.17151081	2.38342817	15.25361310
H	4.39919532	5.53280738	14.70545908
H	7.46606952	-4.51537633	14.43870903
H	8.10346960	12.43244187	15.51868211
H	15.30218612	-4.30950331	15.26123611
H	0.26783202	12.22524987	14.69193008
H	8.11211556	7.67913157	15.25669911
H	7.45808355	0.23780302	14.70320405
H	0.28487902	7.91249355	14.44004505
H	15.28574109	0.00311900	15.51546112
H	4.37518731	14.59973703	15.26113208
H	11.19425479	-6.68322549	14.69297007
H	8.73135661	-6.64389646	14.44015903
H	6.83804048	14.56085404	15.51458813
H	16.49815618	-2.15596915	15.51282010
H	-0.92768607	10.07148074	14.44010701
O	2.88954421	7.66452056	14.70009206
O	2.87122621	12.46123091	14.97788106
O	7.01823253	10.05130173	15.26172809
O	12.68076189	0.25139702	15.25891609
O	8.55149863	-2.13462615	14.69841504
O	12.69840291	-4.54515833	14.97856307
C	6.34079945	6.45870347	14.98088007
C	9.22968367	1.45789911	14.97854105
C	7.07557349	5.18720237	14.98054007
C	6.50438845	4.01526929	15.48652110
C	8.37581559	5.09681537	14.47367003
C	7.19480352	2.81985520	15.48581613
H	5.51475037	4.04991529	15.92748117
C	9.06622167	3.90139428	14.47295405
H	8.84003961	5.97166445	14.03304599
C	8.49508759	2.72946620	14.97903509
H	6.73053150	1.94497514	15.92634016
H	10.05577571	3.86673628	14.03181501

TOT-TOT

C	10.01711572	2.50769118	14.89611905
C	5.17493637	6.26389845	14.48825807
C	7.92696655	-1.21108809	15.16171507
C	6.99549151	9.82631271	15.99284917
C	12.19484890	-1.16897009	14.88560007

C	3.00337122	9.94410270	14.46251804
C	7.09049549	7.37061451	15.47045814
C	7.92639959	1.29945209	15.06302806
C	3.09534122	7.57311853	13.96502999
C	12.19201389	1.25374909	14.79646609
C	4.98780836	11.09057277	15.49452713
C	10.02462173	-2.42318818	15.07127407
C	9.31486168	3.69737827	14.90186306
C	5.87461043	5.07270937	14.49815606
C	7.24827254	-2.41836517	15.25083810
C	7.58566352	10.98223680	16.48438318
C	13.58007698	-1.15698308	14.79548107
C	1.70783813	9.98415173	13.96537803
C	7.77275054	6.16937046	15.46099211
C	7.24624050	2.50192818	15.05758008
C	1.79929613	7.63934353	13.47312698
C	13.57687096	1.23904309	14.70689908
C	5.60042242	12.23297189	15.99127116
C	9.32261069	-3.61745126	15.16095110
C	9.33591865	1.27341009	14.97944806
C	5.76868941	7.44746656	14.97921110
C	9.33435266	-1.19411008	15.07002109
C	5.67925840	9.86218373	15.48714410
C	11.47460782	0.04278200	14.88775906
C	3.72183627	8.73133962	14.47046104
C	7.91880955	3.72561927	14.97972906
C	7.18532252	4.99503836	14.97966106
C	7.93952557	-3.62008826	15.25049609
C	6.89441849	12.18395388	16.48537618
C	14.27292103	0.04009100	14.70635905
C	1.10382408	8.83862565	13.47152400
C	10.04668672	0.04348600	14.97909706
C	5.05820736	8.67753865	14.97896709
H	9.89162773	4.61099033	14.81668907
H	5.37285239	4.20251330	14.09123299
H	6.16720946	-2.38770317	15.31961712
H	8.59827562	10.91185977	16.86389619
H	14.09541101	-2.11028115	14.79731907
H	1.19049908	10.93628776	13.97757202
H	8.77717563	6.16936644	15.86806912
H	6.16669145	2.45837618	15.14285609
H	1.35430810	6.72692648	13.09385093
H	14.09005801	2.19104916	14.63839507
H	5.03416836	13.15684795	15.97861514

H	9.89125271	-4.53994133	15.15840607
H	7.40058854	-4.55745833	15.31983710
H	7.36398652	13.08110094	16.87080423
H	15.35415313	0.03903700	14.63664805
H	0.09217701	8.88007362	13.08580892
C	3.60814226	11.18804982	14.97844109
C	3.79886128	6.27611647	13.94779003
C	7.76737357	8.56882262	16.01074213
O	3.27515524	5.26686838	13.51202595
O	8.90319062	8.51828662	16.44676820
O	2.99886621	12.24292587	14.97814708
C	7.15163853	0.04443000	15.16532808
C	11.49131982	2.55239818	14.79361207
C	11.49729984	-2.46674318	14.97817206
O	12.10682588	-3.52147525	14.97775607
O	5.93674743	0.04905200	15.24756609
O	12.09390088	3.60736226	14.71183207

TOT-CC-TOT

C	10.73295378	1.24537509	15.18800110
C	4.55108333	7.16572650	14.76987604
C	8.67919963	-2.45373718	14.54903503
C	6.60487847	10.86481481	15.40884608
C	12.90652092	-2.43235818	15.19794207
C	2.37755717	10.84348378	14.75994105
C	6.62118449	8.36212461	15.19110007
C	8.66286562	0.04895300	14.76677908
C	2.39095417	8.42934060	14.55078803
C	12.89309691	-0.01821400	15.40709213
C	4.52019933	12.08184887	15.19639412
C	10.76389277	-3.67074726	14.76148805
C	10.03575170	2.43585118	15.18661111
C	5.24827138	5.97524243	14.77126307
C	8.01421957	-3.65420326	14.34180202
C	7.26987250	12.06527387	15.61608010
C	14.27880601	-2.42919917	15.40823213
C	1.00527107	10.84034078	14.54965305
C	7.30465553	7.16366753	15.18959409
C	7.97938156	1.24740209	14.76828306
C	1.01890107	8.45241760	14.34296505
C	14.26514900	-0.04127600	15.61491612
C	5.20807338	13.26941295	15.40590709
C	10.07603272	-4.85831935	14.55197503
C	10.06138875	0.01819100	14.97761007
C	5.22266238	8.39290262	14.98026908

C	10.07309674	-2.44205718	14.76345109
C	5.21098137	10.85315179	15.19443008
C	12.18699586	-1.22028409	15.19390508
C	3.09706822	9.63140170	14.76397508
C	8.70614163	-4.85534635	14.34236702
C	6.57796446	13.26642394	15.61551515
C	14.96025205	-1.24057509	15.61640713
C	0.32381202	9.65172470	14.34147705
C	10.77152176	-1.21073009	14.97825509
C	4.51254233	9.62183169	14.97962507
H	10.58527274	3.35472924	15.35138511
H	4.69874034	5.05637136	14.60648807
H	6.94315249	-3.61833626	14.18088800
H	8.34093858	12.02939386	15.77699512
H	14.79304707	-3.38308124	15.40353113
H	0.49104104	11.79422886	14.55435506
H	8.37516862	7.18108450	15.35440412
H	6.90886852	1.22997309	14.60347207
H	0.51516304	7.50633652	14.18315701
H	14.76887706	0.90481106	15.77472416
H	4.63852433	14.19132904	15.39953209
H	10.64559175	-5.78022844	14.55835103
H	8.17828161	-5.78743641	14.17967201
H	7.10583449	14.19850802	15.77821011
H	16.03132316	-1.24851109	15.77961714
H	-0.74725905	9.65967268	14.17826805
C	6.63435148	5.95026441	14.98018410
C	8.64967164	2.46081318	14.97769008
C	7.33919954	4.73020834	14.97943907
C	7.94480956	3.68086126	14.97843210
C	12.22273586	-3.72226727	14.98040307
C	12.19411890	1.28068509	15.41400413
C	7.90289957	-1.19950708	14.54050204
C	7.38116451	9.61057567	15.41738113
C	3.08991722	7.13043350	14.54387206
C	3.06135622	12.13338587	14.97748005
O	12.78623591	2.32872817	15.59607212
O	6.69940149	-1.18925308	14.35710302
O	12.83213590	-4.77701034	14.98144909
O	2.45196918	13.18813593	14.97643509
O	2.49778918	6.08239743	14.36180301
O	8.58466164	9.60030767	15.60078211

TOT-CCCC-TOT

C	11.19495680	0.44475603	15.18715611

C	3.73161527	8.58564660	14.77029705
C	9.14171764	-3.25449223	14.54782702
C	5.78429241	12.28512189	15.41010912
C	13.36889996	-3.23277923	15.19788407
C	1.55720911	12.26290586	14.75942204
C	5.80123942	9.78248969	15.19241211
C	9.12511565	-0.75185206	14.76544206
C	1.57132111	9.84867669	14.55028505
C	13.35512098	-0.81854006	15.40692909
C	3.69937226	13.50177298	15.19675110
C	11.22652481	-4.47139132	14.76087606
C	10.49816678	1.63553512	15.18566008
C	4.42855832	7.39495556	14.77181706
C	8.47687961	-4.45503832	14.34047701
C	6.44895147	13.48574399	15.61759312
C	14.74105408	-3.22941923	15.40858308
C	0.18508101	12.25937990	14.54855004
C	6.48558245	8.58445362	15.19128110
C	8.44091958	0.44627103	14.76662604
C	0.19933601	9.87140570	14.34185801
C	14.72713307	-0.84143406	15.61516610
C	4.38690332	14.68941305	15.40650511
C	10.53881876	-5.65895138	14.55123905
C	10.52337673	-0.78214906	14.97663707
C	4.40300631	9.81262268	14.98098908
C	10.53562475	-3.24266123	14.76261504
C	4.39042132	12.27312586	15.19510007
C	12.64915392	-2.02073715	15.19357308
C	2.27710717	11.05095481	14.76378505
C	9.16892068	-5.65608240	14.34126903
C	5.75676842	14.68670605	15.61668913
C	15.42232211	-2.04065115	15.61692212
C	-0.49600904	11.07053278	14.34007801
C	11.23393980	-2.01152914	14.97753207
C	3.69229026	11.04191482	14.98003407
H	11.04725380	2.55463018	15.35055012
H	3.87960628	6.47580246	14.60679404
H	7.40585555	-4.41931232	14.17928401
H	7.51995453	13.45014396	15.77895313
H	15.25545707	-4.18321530	15.40408709
H	-0.32944102	13.21311098	14.55301206
H	7.55601854	8.60177560	15.35648611
H	7.37045555	0.42906703	14.60159207
H	-0.30413002	8.92522165	14.18183200

H	15.23073809	0.10469301	15.77509013
H	3.81720927	15.61123712	15.39987611
H	11.10840181	-6.58084446	14.55779307
H	8.64120163	-6.58822648	14.17846702
H	6.28435444	15.61891411	15.77955512
H	16.49333818	-2.04847915	15.78046912
H	-1.56700911	11.07822982	14.17642101
C	5.81482642	7.37107453	14.98141507
C	9.11186267	1.65957612	14.97629210
C	6.51879446	6.15281145	14.98072407
C	7.12622554	5.09961337	14.97951606
C	7.80063855	3.93113928	14.97820810
C	8.40806863	2.87794021	14.97701305
C	8.36526459	-2.00048315	14.53899405
C	12.68531393	-4.52276633	14.98018510
C	12.65615790	0.48024103	15.41363413
C	6.56090649	11.03121279	15.41898110
C	2.27044116	8.54998063	14.54368102
C	2.24060716	13.55297300	14.97723610
O	13.24788596	1.52843811	15.59560210
O	13.29480896	-5.57742242	14.98140808
O	7.16183451	-1.99012714	14.35562302
O	1.67894412	7.50175755	14.36110402
O	7.76422454	11.02095178	15.60308414
O	1.63098612	14.60755505	14.97593607

TOT-Ph-TOT

C	11.31991581	0.24104002	15.12213610
C	4.24923231	7.67656554	14.83572008
C	9.24721367	-3.46766925	14.67075807
C	6.32203048	11.38531985	15.28647310
C	13.49856495	-3.43631925	15.13427310
C	2.07066115	11.35396281	14.82323605
C	6.32829546	8.87826664	15.12627210
C	9.24088064	-0.96066307	14.83166807
C	2.08006415	8.93339066	14.67461006
C	13.48909697	-1.01576207	15.28327412
C	4.22938531	12.60175292	15.13614210
C	11.33992681	-4.68407134	14.82050306
C	10.61558375	1.43115410	15.12111310
C	4.95354835	6.48644045	14.83687507
C	8.57381562	-4.67190334	14.52069907
C	6.99551251	12.58958693	15.43586111
C	14.87830308	-3.42657824	15.28609908
C	0.69088005	11.34419880	14.67183606

C	7.00799353	7.67388356	15.12502711
C	8.56115363	0.24370802	14.83308208
C	0.70042705	8.95087965	14.52480606
C	14.86869105	-1.03328007	15.43350310
C	4.92657935	13.79253001	15.28702509
C	10.64281177	-5.87481342	14.66894707
C	10.64433575	-0.98944207	14.97688208
C	4.92486535	8.90701165	14.98090605
C	10.64964779	-3.45456325	14.82338009
C	4.91960735	11.37219884	15.13377811
C	12.77773590	-2.22448016	15.13087610
C	2.79148120	10.14210474	14.82674206
C	9.26485067	-5.87377041	14.51899607
C	6.30455647	13.79149901	15.43686210
C	15.56519314	-2.23198216	15.43572109
C	0.00392100	10.14958074	14.52268703
C	11.35623679	-2.22109016	14.97700909
C	4.21298130	10.13871271	14.98052208
H	11.18457079	2.34272417	15.26152510
H	4.38467831	5.57480839	14.69642904
H	7.49670052	-4.63876734	14.40616304
H	8.07261560	12.55643090	15.55049413
H	15.39431509	-4.37952532	15.28331910
H	0.17488701	12.29715691	14.67455407
H	8.08192060	7.71173958	15.26527310
H	7.48721152	0.20569401	14.69297007
H	0.19151101	8.00095357	14.41063306
H	15.37758912	-0.08336701	15.54786714
H	4.35847231	14.71536507	15.28339911
H	11.21096383	-6.79762250	14.67214605
H	8.72975961	-6.80852847	14.40121402
H	6.83970649	14.72629708	15.55405714
H	16.64224919	-2.23487216	15.55366512
H	-1.07317608	10.15246174	14.40513305
C	6.34270544	6.45398845	14.98081509
C	9.22641669	1.46361510	14.97730707
C	7.07804051	5.18158537	14.98008810
C	6.48862945	4.00675629	15.45391611
C	8.38991758	5.10612037	14.50531107
C	7.17923153	2.81144020	15.45292009
H	5.48458240	4.03419129	15.86086913
C	9.08052564	3.91080828	14.50432204
H	8.86730965	5.99020142	14.09914800
C	8.49108363	2.73598220	14.97808707

H	6.70182650	1.92740314	15.85915216
H	10.08459973	3.88342828	14.09743601
C	2.76237520	12.64857490	14.97938107
C	2.78140120	7.63494755	14.67231004
C	7.09702252	10.12943671	15.28948010
C	8.47211363	-2.21185916	14.66871106
C	12.78779990	0.28269202	15.28523610
C	12.80692193	-4.73089934	14.97747407
O	13.41668694	-5.78582740	14.97756610
O	13.38757897	1.33560810	15.41228013
O	8.30870258	10.12357374	15.41720312
O	7.26032953	-2.20606416	14.54198205
O	2.15265115	13.70353097	14.97894106
O	2.18166116	6.58205850	14.54485003

PLY-PLY

C	10.03138371	2.49866818	14.78713108
C	5.19121637	6.29097847	14.38275404
C	7.04795149	7.36709252	15.57406512
C	7.93460358	1.28227309	15.17241011
C	9.30088066	3.70216127	14.79265807
C	5.91776944	5.08513937	14.39823401
C	7.73261356	6.13700646	15.55921011
C	7.25238653	2.51386218	15.16601908
C	9.34144569	1.27240809	14.97998708
C	5.76151742	7.44731055	14.97829006
C	7.92139558	3.72032127	14.97914908
C	7.18012251	4.99860136	14.97881908
C	11.42724682	2.46800818	14.58994805
C	3.91615328	6.39233644	13.78927800
C	7.26786454	0.05538400	15.37038109
C	7.59468557	8.52372460	16.16759217
C	10.05384072	0.04444900	14.98051806
C	5.04993737	8.67572362	14.97813110
H	9.83106869	4.63191633	14.61725206
H	5.48728941	4.21351930	13.91729799
H	8.70274765	6.07712746	16.04046014
H	6.18199944	2.51525718	15.34103911
H	8.57537564	8.46498963	16.62699020
H	6.19392445	0.05948300	15.52165710
H	3.47834425	5.51285941	13.32979795
H	11.95773987	3.40175024	14.43835501
C	12.11574685	1.26651809	14.58970505
H	13.18937097	1.26552409	14.43716405
C	11.45015882	0.06871200	14.78199007

H	11.99634187	-0.86838306	14.78108306
C	9.33961569	-1.15508308	15.17957009
H	9.88125269	-2.09483515	15.18142010
C	7.96903757	-1.13837608	15.37155412
H	7.43727852	-2.07098715	15.52453714
C	3.22826523	7.59417555	13.79093697
H	2.24838716	7.65002354	13.32978694
C	3.77620727	8.72316664	14.37392102
H	3.23108023	9.66087772	14.37176605
C	5.64234242	9.80398169	15.58208914
H	5.10070437	10.74373175	15.58431814
C	6.89450549	9.71803569	16.16546715
H	7.33314353	10.59619978	16.62629121

PLY-CC-PLY

C	7.95568955	1.53480411	14.37008205
C	3.81033427	8.68349263	14.36797703
C	5.65783741	9.75472868	15.58701213
C	9.80281772	2.60579119	15.58990413
C	7.26810955	2.76066020	14.38127901
C	4.53248432	7.47765056	14.37949606
C	6.34548044	8.52890863	15.57571911
C	9.08070964	3.81165927	15.57832912
C	9.23666066	1.45384511	14.98002307
C	4.37665031	9.83554770	14.97750307
C	7.41300653	0.37848003	13.77065398
C	2.53707718	8.78698563	13.76877900
C	11.07588578	2.50217418	16.18947815
C	6.20065145	10.91114581	16.18614216
C	9.94872469	0.22559202	14.98002206
C	3.66453226	11.06376878	14.97758107
H	6.29099244	2.82812421	13.91761698
H	4.10540329	6.59601650	13.91623101
H	7.32274255	8.46154162	16.03908814
H	9.50768869	4.69322134	16.04182314
H	7.17821752	10.85128978	16.65168320
H	11.50951584	3.38016124	16.65536818
H	2.10333715	7.90891859	13.30314196
H	6.43559446	0.43844003	13.30480194
C	8.11353259	-0.81496206	13.77494698
H	7.67963455	-1.69257812	13.30912294
C	9.36190469	-0.90090606	14.36723703
H	9.90445272	-1.83996413	14.36630705
C	11.21790483	0.17515901	15.59281810
H	11.76332687	-0.76223405	15.59378014

C	11.76350885	1.30123209	16.18518716
H	12.74068592	1.24174309	16.65108518
C	1.84942313	9.98790672	13.77310301
H	0.87211706	10.04731375	13.30746497
C	2.39516717	11.11407782	14.36516303
H	1.84970513	12.05144785	14.36424803
C	4.25148830	12.19036386	15.59006310
H	3.70889227	13.12939296	15.59106012
C	5.50005041	12.10454586	16.18196616
H	5.93403841	12.98222195	16.64759219
C	5.79146742	7.39526454	14.97773506
C	7.82195154	3.89419128	14.97963809
C	7.11002753	5.12197137	14.97902006
C	6.50345049	6.16752046	14.97825710

PLY-CCCC-PLY

C	7.91937656	1.59665711	14.37086904
C	2.48910218	10.96329379	14.36631303
C	4.33733331	12.03387588	15.58700313
C	9.76827869	2.66771719	15.59012311
C	7.23147752	2.82192320	14.38199102
C	3.20998023	9.75708273	14.37693103
C	5.02519436	10.80859078	15.57586210
C	9.04737867	3.87391228	15.57945812
C	9.20080065	1.51618411	14.98030009
C	3.05625622	12.11459489	14.97687007
C	7.37597254	0.44045503	13.77165201
C	1.21596709	11.06734782	13.76675299
C	11.04174379	2.56389618	16.18902015
C	4.88043235	13.18985893	16.18691518
C	9.91259173	0.28779802	14.97983209
C	2.34449117	13.34299594	14.97737210
H	6.25414948	2.89063621	13.91920202
H	2.78370020	8.87531864	13.91341600
H	6.00224846	10.73968079	16.03920118
H	9.47390866	4.75585934	16.04239513
H	5.85780445	13.12940196	16.65265218
H	11.47573983	3.44177625	16.65463421
H	0.78221006	10.18963374	13.30060297
H	6.39834647	0.50073604	13.30642498
C	8.07647960	-0.75282405	13.77565698
H	7.64244054	-1.63047512	13.31014097
C	9.32518868	-0.83854006	14.36735101
H	9.86768770	-1.77760313	14.36615206
C	11.18210382	0.23707202	15.59187114

H	11.72716782	-0.70050805	15.59237814
C	11.72872984	1.36276010	16.18410515
H	12.70613991	1.30270409	16.64933318
C	0.52899904	12.26848990	13.77171900
H	-0.44815503	12.32873286	13.30597497
C	1.07532208	13.39396296	14.36464703
H	0.53027104	14.33155006	14.36417204
C	2.93158021	14.46911005	15.59057312
H	2.38910117	15.40818509	15.59178912
C	4.17995830	14.38316003	16.18292519
H	4.61376433	15.26063909	16.64898519
C	7.78785056	3.95477928	14.98073809
C	7.07777349	5.17983037	14.98030210
C	4.46914832	9.67596769	14.97636709
C	6.46862549	6.22937144	14.97919210
C	5.78839544	7.40139353	14.97792107
C	5.17920337	8.45090960	14.97678408

PLY-Ph-PLY

C	9.93995072	2.49131418	14.50158603
C	2.96702022	10.01968672	15.45666910
C	4.92294336	11.15261479	14.50135404
C	7.98387559	1.35828910	15.45654813
C	9.21184966	3.69586426	14.51196807
C	3.69515127	8.81516061	15.44637211
C	5.60552639	9.92169871	14.51184604
C	7.30126753	2.58919819	15.44602110
C	9.31995565	1.30670909	14.97908907
C	3.58692626	11.20425783	14.97897609
C	11.27057882	2.42187618	14.03821801
C	1.63642412	10.08913875	15.92013915
C	7.38206252	0.16953101	15.91998416
C	5.52472542	12.34134189	14.03778599
C	10.03170572	0.07790001	14.97917210
C	2.87517221	12.43305788	14.97886110
H	9.70233971	4.60162933	14.17216002
H	3.20468123	7.90948857	15.78646115
H	6.63520747	9.89653670	14.17187103
H	6.27150046	2.61438819	15.78573316
H	6.54647847	12.30634189	13.67573101
H	6.36028248	0.20451101	16.28196616
H	1.15840108	9.18545467	16.28226816
H	11.74863384	3.32558124	13.67618499
C	11.95924787	1.22118409	14.04247503
H	12.98100293	1.18845308	13.68114396

C	11.35863984	0.06160100	14.50242007
H	11.90517585	-0.87518106	14.50173907
C	9.38563967	-1.08120508	15.45597413
H	9.92634970	-2.02136215	15.45674409
C	8.08092458	-1.02524907	15.91586113
H	7.60094156	-1.92781114	16.27725918
C	0.94773207	11.28981780	15.91582414
H	-0.07398901	11.32255979	16.27724918
C	1.54828111	12.44937792	15.45573812
H	1.00172307	13.38614696	15.45637213
C	3.52119525	13.59212497	14.50190502
H	2.98049522	14.53228805	14.50113406
C	4.82587235	13.53612796	14.04190902
H	5.30582537	14.43865903	13.68039396
C	7.90154657	3.75540827	14.97897710
C	5.00537436	8.75551763	14.97913908
C	5.74510541	7.47846952	14.97915209
C	6.61738547	7.14960353	13.94123500
C	5.59639538	6.55811450	16.01698616
C	7.31052152	5.95280341	13.94112700
H	6.72829250	7.82826758	13.10286695
C	6.28955546	5.36132839	16.01691117
H	4.95253636	6.79958151	16.85537220
C	7.16180953	5.03246836	14.97897408
H	7.95432856	5.71125241	13.10272592
H	6.17875944	4.68267634	16.85530320

Planar TRI(N)-TRI(N)

C	9.729694	2.314639	14.118053
C	5.377604	6.407609	15.842417
C	8.540165	-0.801517	16.706289
C	6.567393	9.523753	13.254291
C	11.837791	-1.352884	14.124642
C	3.269473	10.075109	15.835567
C	6.870189	7.280556	14.113116
C	8.237301	1.441694	15.847523
C	3.584281	7.799388	16.700122
C	11.522915	0.922851	13.260143
C	4.750270	10.941204	14.120019
C	10.357141	-2.218996	15.840305
C	9.018482	3.515515	14.133941
C	6.088833	5.206738	15.826618
C	8.175202	-1.871545	17.557175
C	6.932413	10.593767	12.403404

C	12.939160	-1.500669	13.244351
C	2.168020	10.222899	16.715750
C	7.548693	6.041605	14.144602
C	7.558796	2.680650	15.816109
C	2.475072	8.005691	17.557723
C	12.632034	0.716547	12.402425
C	5.157751	11.963904	13.255979
C	9.949719	-3.241714	16.704354
C	9.344948	1.252576	14.982327
C	5.762444	7.469672	14.978185
C	9.649646	-0.982537	15.835452
C	5.457797	9.704764	14.124984
C	11.126318	-0.126542	14.128199
C	3.980966	8.848777	15.832104
C	7.925084	3.718042	14.980068
C	7.182342	5.004225	14.980630
C	8.867111	-3.057734	17.550396
C	6.240458	11.779927	12.410061
C	13.323603	-0.478484	12.400513
C	1.783500	9.200721	17.559561
C	10.829423	2.121499	13.261862
C	4.277772	6.600737	16.698475
C	7.851294	0.408954	16.698210
C	7.256302	8.313309	13.262487
C	11.451298	-2.377518	14.971521
C	3.656028	11.099726	14.988697
H	9.354392	4.287780	13.456854
H	5.752776	4.434441	16.503593
H	7.328919	-1.726961	18.218095
H	7.778772	10.449186	11.742581
H	13.473095	-2.443621	13.253031
H	1.634079	11.165847	16.707009
H	8.386324	5.942834	13.468354
H	6.721283	2.779437	16.492497
H	2.183625	7.195101	18.215170
H	12.923415	1.527139	11.744951
H	4.608578	12.897360	13.265241
H	10.498868	-4.175184	16.695006
H	8.101769	8.175115	12.599294
H	7.005934	0.547165	17.361535
H	3.111323	12.036712	14.992288
H	11.995986	-3.314512	14.967856
H	3.972046	5.800919	17.362941
H	11.135064	2.921312	12.597352

H	8.563250	-3.859649	18.214326
H	6.544360	12.581826	11.746129
H	14.168947	-0.613820	11.735870
H	0.938090	9.336059	18.224121
N	5.066360	8.674412	14.979018
N	10.041013	0.047827	14.981400
